# Red pigments in autumn leaves of Norway maple do not offer significant photoprotection but coincide with stress symptoms

**DOI:** 10.1093/treephys/tpad010

**Published:** 2023-01-30

**Authors:** Heta Mattila, Esa Tyystjärvi

**Affiliations:** Department of Life Technologies/Molecular Plant Biology, University of Turku, 20014 Turku, Finland; Centre for Environmental and Marine Studies (CESAM), Department of Biology, University of Aveiro, Portugal; Department of Life Technologies/Molecular Plant Biology, University of Turku, 20014 Turku, Finland

**Keywords:** *Acer platanoides*, autumnal, chlorophyll fluorescence, deciduous, nutrient resorption

## Abstract

The reasons behind autumn colors, a striking manifestation of anthocyanin synthesis in plants, are poorly understood. Usually, not all leaves of an anthocyanic plant turn red or only a part of the leaf blade turns red. In the present study, we compared green, red and yellow sections of senescing Norway maple leaves, asking if red pigments offer photoprotection, and if so, whether the protection benefits the senescing tree. Green and senescing maple leaves were illuminated with strong white, green or red light in the absence or presence of lincomycin which blocks photosystem II (PSII) repair. Irrespective of the presence of anthocyanins, senescing leaves showed weaker capacity to repair PSII than green leaves. Furthermore, the rate of photoinhibition of PSII did not significantly differ between red and yellow sections of senescing maple leaves. We also followed pigment contents and photosynthetic reactions in individual leaves, from the end of summer until abscission of the leaf. In maple, red pigments accumulated only during late senescence, but light reactions stayed active until most of the chlorophyll had been degraded. PSII activity was found to be lower and non-photochemical quenching higher in red leaf sections, compared with yellow sections of senescing leaves. Red leaf sections were also thicker. We suggest that the primary function of anthocyanin synthesis is not to protect senescing leaves from excess light but to dispose of carbohydrates. This would relieve photosynthetic control, allowing the light reactions to produce energy for nutrient translocation at the last phase of autumn senescence when carbon skeletons are no longer needed.

## Introduction

During autumn senescence, deciduous plants degrade photosynthetic protein complexes and other macromolecules in senescing leaves, to remobilize nutrients for winter storage. Protective mechanisms, such as accumulation of tocopherols and xanthophylls, are often upregulated (e.g., Junker and Ensminger 2016), possibly to enable efficient nutrient resorption while photosynthesis declines (Krieger-Liszkay et al. 2019, Mattila et al. 2021). Many plant species synthetize anthocyanins during autumn, and it has been proposed that anthocyanins protect senescing leaves from (excess) light (Hoch et al. 2003, Renner and Zohner 2019).

In non-senescing plants, anthocyanin synthesis is often upregulated in high light (e.g., Viola et al. 2016), possibly via chloroplast retrograde signaling (Richter et al. 2020). Also, in senescing leaves of, e.g., red-osier dogwood (*Cornus sericea* L.) and maple (*Acer*), anthocyanin accumulation further increases in high light (Feild et al. 2001, Lee et al. 2003, Duan et al. 2014). Although it has often been concluded that anthocyanins protect photosystem II (PSII) of young or mature leaves from light-induced damage (photoinhibition) (see the summary of Gould et al. 2018 and the review of Agati et al. 2021), only a small number of these studies (Feild et al. 2001, Hoch et al. 2003, Manetas and Buschmann 2011) have been conducted on senescing autumn leaves. In addition, several investigations on senescing leaves have failed to find a protective effect or have found only a minor effect (Lee et al. 2003, van den Berg and Perkins 2007, van den Berg et al. 2009, Duan et al. 2014, Lo Piccolo et al. 2018; see also the literature analysis by Gould et al. 2018). Whether the distribution of yellow and redsenescing species in northern America and Europe supports the photoprotection hypothesis is also disputed (Renner and Zohner 2019, 2020, Pena-Novas and Archetti, 2020*a*, 2020*b*). On the other hand, photoprotection may not be the (primary) function of anthocyanins (see Manetas, 2006, Renner and Zohner 2019, Pena-Novas and Archetti, 2021*b*). A competing (but not mutually exclusive) hypothesis, suggesting that anthocyanins are involved in defense against insect herbivores (the co-evolution hypothesis), is also commonly used to explain autumn colors (Furuta, 1986; for a review, see Lev-Yadun and Gould 2007).

Anthocyanins could protect leaves by screening light and/or by functioning as antioxidants. Anthocyanin accumulation has been shown to attenuate light in senescing leaves of red-osier dogwood and Norway maple (*Acer platanoides* L.) (Feild et al. 2001, Merzlyak et al. 2008). In vitro, anthocyanins are able to quench superoxide, hydrogen peroxide and singlet oxygen (Sondheimer and Kertesz 1952, Neill and Gould 2003, De Rosso et al. 2008). Some studies have suggested that this is the case also in vivo (e.g., Gould et al. 2002); however, evidence from senescing leaves is scarce (van den Berg and Perkins 2007). The efficacy of anthocyanins as antioxidants depends on their localization within leaves (Kytridis and Manetas 2006). In senescing maple leaves, anthocyanins are mainly localized in the vacuoles of palisade mesophyll cells (Merzlyak et al. 2008, Junker and Ensminger 2016) and therefore their impact on chloroplast-localized reactive oxygen species is expected to be low. Screening of light by anthocyanins, however, may indirectly decrease the production of reactive oxygen species (e.g., Zhang et al. 2016).

Anthocyanins absorb green light efficiently. Indeed, vacuole-located anthocyanins protected PSII in green but not in red light and only when the light was shone on the anthocyanic adaxial (upper) side of beetleweed (*Galax urceolata* (Poir.) Brummitt) leaves (Hughes et al. 2005). It is often mentioned that green light is not well absorbed by chlorophylls and therefore does not necessarily contribute to ‘excess’ light (Manetas, 2006, Pena-Novas and Archetti, 2021*b*). However, the contribution of green light to photosynthetic processes is not insignificant (e.g., Nichelmann and Bilger 2017, Hughes et al. 2022). The extent of PSII photoinhibition is also not (at least not solely; see Tyystjärvi, 2013) determined by the absorption profiles of chlorophylls, and green light has a relatively high capacity to damage PSII (Hakala et al. 2005). In addition, some anthocyanins absorb blue (or even red) light (e.g., Feild et al. 2001, Merzlyak et al. 2008; see also Agati et al. 2021); blue light is highly efficient in damaging PSII (Jones and Kok 1966, Hakala et al. 2005, Zavafer et al. 2015).

The benefit (or lack of it) obtained by protecting senescing leaves should also be considered. Nutrient resorption efficiencies greatly vary among species and environmental conditions; a high resorption efficiency may improve plant fitness, especially if the nutrient content of the soil is low (May and Killingbeck 1992; for a review, see Brant and Chen 2015). It has been suggested that plants suffering from nitrogen deficiency benefit more from anthocyanins than well fertilized plants (Hughes et al. 2022). In fact, anthocyanin accumulation may be a common response to nitrogen limitation (Schaberg et al. 2003, Hughes et al. 2022). Earlier research has also shown that high leaf sugar content induces anthocyanin accumulation in maple and other species (Murakami et al. 2008, K.M. Zhang et al. 2013, Schaberg et al. 2017). In aspen, during late phases of senescence, starch content decreases and glucose and fructose become the dominant soluble sugars (Keskitalo et al. 2005).

Nutrient resorption efficiencies of anthocyanic and non-anthocyanic leaves have rarely been compared, and the results are contradictory (Feild et al. 2001, Hoch et al. 2003, Duan et al. 2014, Pena-Novas and Archetti, 2021*a*). Schaberg et al. (2008) observed that in sugar maple (*Acer saccharum* Marsh.) red senescing leaves contained slightly more chlorophyll and were more firmly attached, due to a less formed abscission layer, than yellow senescing leaves. The authors suggested that delayed senescence in red leaves could promote nutrient resorption. However, the duration of senescence in individual leaves was not evaluated. On the other hand, Y.J. Zhang et al. (2013) suggested that in *Lyonia ovalifolia* Drude, which retains red senescing leaves for several months during winter, delayed senescence offers a competitive benefit by increasing carbon gain.

Some leaves of a senescing Norway maple tree turn red (mostly due to anthocyanin accumulation) while other leaves remain yellow. Furthermore, a senescing maple leaf may contain dark green, pale green, yellow and red sections at the same time. Therefore, maple is an excellent model plant for studying the roles of anthocyanins in autumn senescence. In the present study, we addressed the question of whether red pigments protect senescing leaves and whether such protection offers a fitness advantage for the senescing tree. To test the photoprotection hypothesis, green, yellow and red sections of senescing leaves of Norway maple were exposed to high light; a pair of red and yellow sections was always obtained from a single senescing leaf, to minimize differences other than anthocyanin content. To accurately probe the damaging reaction of photoinhibition of PSII, we repeated the treatments in the presence of lincomycin, an antibiotic that inhibits PSII repair, because in the absence of lincomycin, damage and repair occur simultaneously (for reviews, see Nath et al. 2013, Tyystjärvi, 2013). To our knowledge, lincomycin has not been previously tested with senescing leaves. In addition, pigment contents and photosynthetic reactions were followed in individual leaves thorough the autumn, until abscission. The question whether photoprotection offers fitness advantage was approached by measurements of leaf thickness and protein content to find out whether anthocyanins enhance dislocation of valuable substances from the senescing leaf. Leaf protein content also correlates well with leaf nitrogen content in senescing leaves (Yasumura et al. 2007). The protein measurements were done on aspen leaves because an aspen tree typically has leaves that turn red and others that remain yellow, which allows testing of the relationship between redness and transport of nutrients at a leaf level.

## Materials and methods

### Plant material

Norway maple (*A. platanoides*) and European aspen (*Populus tremula* L.) leaves were collected during the autumn of 2020 and 2021 from trees growing in small city parks (Turku, Finland). Laboratory measurements were conducted on the same day as the leaves were collected, if not otherwise mentioned.

The course of senescence in vivo was followed in attached leaves, in their natural environment. For that reason, four to eight leaves per tree across four maple trees were selected. All the trees, growing at 60°26′31.7″N 22°14′38.7″E, had started to senesce at the beginning of the measurements but only leaves that did not yet show any visible signs of senescence were selected for the measurements. Due to a different timing of senescence in each tree, measurements were started between 3 September 2021 and 2 October 2021; the last measurement was conducted on 26 October 2021. Several parameters were recorded from the selected leaves, almost on a daily basis, with a handheld device (MultispeQ v1, PhotosynQ Inc., East Lansing, MI, USA; see below) from six sites on each leaf, until the leaf dropped. The measurements were conducted between 9:00 and 12:00h.

### MultispeQ measurements

A leaf was gently placed between the measuring heads of the MultispeQ. The MultispeQ measurements covered (i) absorbance at several wavelengths, (ii) pulse amplitude modulated chlorophyll *a* fluorescence, (iii) oxidation of the primary donor (P700) of photosystem I (PSI), assayed by near-infrared absorbance changes, (iv) electrochromic shift, assayed by 530-nm absorbance changes during a light-to-dark transition, (v) leaf thickness, (vi) leaf temperature, (vii) ambient light, (viii) temperature, (ix) pressure and (x) humidity. For the analysis, the last temperature recording of the day was used, as this choice allowed the device temperature to equilibrate with ambient temperature. The measuring protocol (Photosynthesis RIDES) illuminated the leaves with photosynthetic photon flux density (PPFD) matching that of the incident light. The fluorescence parameter *F*_0_′ was measured during far-red illumination, *F*_S_ during a period of simulated incident light and *F*_M_′ during a saturating pulse. Calculations of non-photochemical quenching (NPQt and ΦNPQ) and non-regulated quenching (ΦNO) of fluorescence are based on Tietz et al. (2017) and Kuhlgert et al. (2016). qL was calculated as (*F*_M_′ − *F*_S_)/(*F*_M_′ − *F*_0_′)×(*F*_0_′/*F*_S_), NPQt as (4.88/((*F*_M_′/*F*_0_′) − 1)) − 1 and ΦNO as 1/(NPQt +1 + qL×4.88). The PSI parameters are described by Kanazawa et al. (2017). Parameters (vH+, gH+) derived from the measurements of electrochromic shift are based on Cruz et al. (2001), Kanazawa and Kramer (2002) and Avenson et al. (2005). Chlorophyll content was estimated from the absorbance of leaves at 650 and 940 nm, employing the so-called Minolta SPAD method (see e.g., Hunt and Daughtry 2014).

### Calibration of pigment measurements

Amounts of red pigments were estimated by measuring absorbance at 530 nm with MultispeQ. Also chlorophylls absorb at 530 nm and therefore absorbance at 530 nm was measured from dark and pale green maple leaves (with no visually detectable redness), collected on 17 September 2021 and 21 September 2021. 530-nm absorbance increased linearly with chlorophyll values measured with the same device (Figure S1a available as Supplementary data at *Tree Physiology* Online) and thus the ‘excess’ (chlorophyll-related) 530-nm absorbance can be subtracted from the absorbance signal (A530) to get a redness index (*R*) = A530 − (0.0113 × SPAD + 0.495), where SPAD refers to the chlorophyll content measured with MultispeQ (Figure S1a available as Supplementary data at *Tree Physiology* Online). Leaf sections with *R* < 0.05 were considered to contain low amounts of red pigments, those with *R* between 0.05 and 0.1 medium amounts and those with *R* > 0.1 high amounts. In some cases, leaf classes with very high (*R* > 0.2) or very low (*R* < 0.025) amounts of red pigments were additionally used. In photoinhibition experiments (see below), leaves were considered ‘red’ if the index was higher than 0.1. The redness index correlated well with visual estimation when leaves were collected from trees (personal observation). However, redness indexes of fallen leaves (colored yellow or brown, collected on 26 October 2021) were unrealistically high (*R* = 0.34 ± 0.02). Therefore, the method should be used with caution if dead leaves or dead leaf parts are used.

To verify that a high concentration of red pigments did not disturb the optical chlorophyll measurements, chlorophyll contents of selected maple leaves (collected 12–14 October 2020, 15 October 2020, 20–21 October 2020 and 21 September 2021) were measured with MultispeQ, after which chlorophylls *a* and *b* were extracted by incubating the leaf discs in dimethylformamide in darkness at 4 °C for 4–10 days, and quantified spectroscopically (Porra et al. 1989). Carotenoids were quantified according to Wellburn (1994) from maple leaves collected on 12 October 2020, 15 October 2020, 20 October 2020 and 21 September 2021. The optical SPAD method utilized by MultispeQ responded non-linearly to actual leaf chlorophyll content but seemed indifferent to the presence of red pigments (Figure S1b available as Supplementary data at *Tree Physiology* Online). However, MultispeQ measurements on brown, fallen leaves produced unrealistically high chlorophyll readings (Figure S1b available as Supplementary data at *Tree Physiology* Online). As parts of senescing maple leaves may appear brown and dead while the leaf is still attached to the tree, leaves with very low chlorophyll content (<0.5 μg cm^−2^) were excluded from some analyses, as indicated.

Chlorophyll values measured with MultispeQ were converted according to the following empirical equation, obtained from the calibration:

Chlorophyll content (*a* + *b*) in maple leaves, μg cm^−2^ = MultispeQ value^2^ × 0.0226 + MultispeQ value × 0.0345.

Absorptance of green, yellow and red pieces (2 cm × 2 cm) of maple leaves, collected on 13 September 2021, was measured with an integrating sphere (Labsphere, North Sutton, NH, USA) coupled with an STS-VIS spectrometer (Ocean Insight, Orlando, FL, USA), as previously described (Mattila et al. 2021).

### Photoinhibition treatments

Maple leaves were collected on 15 September 2021, 22 September 2021, 29 September 2021 and 13 October 2021. Leaves were incubated over-night under low light (PPFD 5–10 μmol m^−2^ s^−1^) with petioles in lincomycin solution (0.6 mg ml^−1^) prepared in tap water. When lincomycin was not used, illumination treatments were conducted on the collection day. Leaves were cut to pieces (~2 cm × 3 cm); fully green and senescing leaves were collected from the same trees. Pieces with pale green color (‘yellow’) and reddish color (‘red’) were cut from a single senescing leaf. Leaf pieces were placed on a wet paper and dark-acclimated for at least 30 min. The fluorescence parameter (*F*_M_ − *F*_0_)/*F*_M_ = *F*_V_/*F*_M_ was measured with a FluorPen (Photon Systems Instruments, Brno, Czech Republic), after which pigments were measured with MultispeQ. Leaf pieces were illuminated for 45 min to 2 h with white (sunlight simulator SLHolland; for the spectrum, see Havurinne et al. 2022), green (500–600 nm) or red (λ > 600 nm) light, PPFD 2000 μmol m^−2^ s^−1^, or incubated in darkness, as indicated. Green and red light were defined with long-pass and short-pass edge filters (LL-500 and LS-600 for green and LL600 for red; Corion, USA). To observe clear photoinhibition but avoid a situation where the *F*_V_/*F*_M_ values would decrease to zero, different illumination times were used for different treatments. During illumination, leaf pieces were kept on wet paper on a temperature-controlled metal block set to 20 °C. After illumination and subsequent 30-min dark-acclimation, *F*_V_/*F*_M_ and MultispeQ measurements were repeated, and leaf pieces were let to recover over-night on a wet paper in low light (PPFD 5–10 μmol m^−2^ s^−1^). After the recovery, leaves were dark-acclimated for 30 min and *F*_V_/*F*_M_ and MultispeQ measurements were repeated. Photoinhibition was quantified via its rate constant *k*_PI_, calculated from the lincomycin-treated samples as *k*_PI_ = ln(*F*_V_/*F*_M__control/*F*_V_/*F*_M__illuminated)/(duration of illumination, min).

### Protein content

To test the efficiency of nutrient resorption, protein content was quantified from senescing red and yellow aspen leaves, collected on 12–22 October 2021. When attached leaves were used, leaves that were loose (about to fall) were selected. Recently fallen leaves (distinguishable by their bright color, as opposed to the brown color of leaves fallen a long time ago) were also used, as indicated. Pigments (with MultispeQ) and fresh weight were measured, after which leaf pieces were ground to fine powder in liquid nitrogen and dissolved in a buffer (50-mM Tris–HCl (pH 8), 2% sodium dodecyl sulfate, 10-mM ethylenediaminetetra-acetic acid) and stored at −20 °C. Protein content was measured from the supernatant after centrifugation with the DCTM Protein assay kit (Bio-Rad, Hercules, CA, USA), which is based on the Lowry method.

### Weather data

Global radiation, temperature and precipitation data were provided by Finnish meteorological institute; all other variables except UV-radiation were measured at a weather station located at about 8 km from the measurement sites (Artukainen, Turku); UV-radiation was measured in Jokioinen, 85 km from the measurement sites.

### Statistical analyses

#### Statistical models

Linear models were constructed for *F*_V_/*F*_M_, for the rate constant of photoinhibition (*k*_PI_) and for recovery from photoinhibition; linear mixed effect models for gH+, vH+, leaf thickness, NPQt and for relative number of active PSI centers; and a beta regression model was used for *F*_V_′/*F*_M_′.

#### Variable transformations

A logit transformation was applied to vH+ data, the leaf thickness was raised to the power 0.85, *k*_PI_ to the power 0.4, recovery from photoinhibition to the power 0.9 (the model for data obtained in the presence and absence of lincomycin) and to the power 0.8 (the model for data obtained in the absence of lincomycin). The exponent was chosen by testing different power transformations and by selecting the exponent so that the transformation led to as normal distribution as possible, as judged by plotting the quantiles of the transformed distribution against theoretical quantiles and by investigating histograms of the transformed distributions. A Yeo–Johnson transformation (Weisberg, 2001) was applied to gH+, *F*_V_/*F*_M_, *F*_V_′/*F*_M_′, NPQt and active PSI centers, with the respective values of the parameter λ of 0.6, 6.5, 1.8, 2.9 and 8.3. In addition, linear and log transformations were applied to independent variables to get them on a similar range. See Summary of statistics available as Supplementary data at *Tree Physiology* Online for detailed information.

#### Filtering

The data obtained by direct measurements from attached leaves were filtered so that only records with chlorophyll content above 0.5 μg cm^−2^ were used. Further filtering to records with chlorophyll content above 5.0 μg cm^−2^ was applied in the models for *F*_V_’/*F*_M_’ and NPQt. Random effects by sample ID, leaf, tree or date was added to models when their inclusion increased the explanatory power of the model. Physiologically impossible values occasionally produced by the handheld device were removed by limiting values of gH+ between 2.0 and 160.0, vH+ values between 0.005 and 0.2, NPQt values between 0 and 25 and active PSI centers between 0 and 5.0. In addition, records with PPFD less than 1 μmol m^−2^ s^−1^ were considered too noisy for the gH+ model, and only records with the *F*_M_’ value between 500 and 4000 were used in the NPQt model. In modeling recovery from photoinhibition, eight records were discarded because the absolute value of the recovery parameter, defined as fraction of the photoinhibitory loss of *F*_V_/*F*_M_ recovered, was larger than 2.0. Data used for the analysis of *F*_V_/*F*_M_ were not filtered.

#### Justification of model parameters

The redness index and chlorophyll content were included in all models, as these parameters are the main topics of the study. PPFD was included as a fixed effect in models of the functional photosynthetic parameters qH+, vH+, *F*_V_’/*F*_M_’ and NPQt. Chlorophyll content and the number of days passed after the start of the measurements on 2 September 2021, describing the advancement of autumn senescence, were included in all mixed effect models. Boxplots of the data also suggested that *F*_V_’/*F*_M_’, NPQt, and the number of active PSI centers would change during the progress of the autumn (Figures S1.5, S1.7 and S1.8 in Summary of statistics available as Supplementary data at *Tree Physiology* Online).

#### Model of gH+

The linear mixed effect model constructed for the analysis of gH+ tested the effects of PPFD, chlorophyll content, redness and number of days after 2 September 2021 as fixed effects, and used the sample ID, tree and days after 2 September 2021 as random effects.

#### Model of vH+

In the linear mixed effect model for vH+, the gH+ parameter was included as a fixed effect because thylakoid proton conductivity affects proton flux. Leaf temperature was also included as a fixed effect, as temperature may obviously affect a flux rate value. Thus, the model for vH+ had PPFD, gH+, chlorophyll content, redness, leaf temperature, number of days after 2 September 2021, and interaction between gH+ and PPFD, between gH+ and chlorophyll content, between gH+ and leaf temperature, and between gH+ and redness as fixed effects, and sample ID, tree, leaf and date as random effects. Leaf temperature was included, as it was clear that leaf temperature varied in the course of the autumn with only a weak decreasing trend and no clear connection to PPFD (Figures S1.1 and S1.2 in Summary of statistics available as Supplementary data at *Tree Physiology* Online).

#### Model for F_V_′/F_M_′

In the beta regression model for *F*_V_′/*F*_M_′, the lowest temperature of the previous night was included as a variable because inspection of the data suggested that this factor affects *F*_V_′/*F*_M_′. Effects of leaf temperature and total radiation of the day were tested because these environmental factors, alone or together with PPFD, may lower *F*_V_′/*F*_M_′ by causing photoinhibition. PSI may be important for the repair of PSII, and therefore the relative number of open PSI centers was tested in the model of *F*_V_′/*F*_M_′. Fluorescence measurements may depend on abiotic environmental factors, and therefore leaf thickness and humidity were also included in the model of *F*_V_′/*F*_M_′. The model for *F*_V_′/*F*_M_′ thus tested the effects of PPFD, chlorophyll content, redness, leaf temperature, lowest temperature of previous night, number of days after 2 September 2021, total radiation, leaf thickness, humidity, relative number of active PSI centers and interaction between PPFD and chlorophyll content, between leaf temperature and lowest temperature of previous night, and between total radiation and lowest temperature of the previous night.

#### Model for leaf thickness

According to descriptive statistics (Figure S1.9 of the Summary of statistics available as Supplementary data at *Tree Physiology* Online), average leaf thickness weakly decreased during the autumn. The linear mixed effect model for leaf thickness tested the effects of leaf temperature and humidity as additional potential fixed effects, as these factors might exert purely physical effects on leaf thickness. Thus, the model for leaf thickness tested chlorophyll content, redness, number of days after 2 September 2021, humidity and leaf temperature as fixed effects and tree, leaf and measurement site on leaf as random effects.

#### Model for NPQt

The linear mixed effect model for NPQt tested the effect of the lowest temperature of the previous night, and leaf temperature, as these factors that seemed to affect *F*_V_′/*F*_M_′ might also affect other fluorescence parameters. Also, like for *F*_V_′/*F*_M_′, leaf thickness and humidity were tested. Thus, the model for NPQt tested chlorophyll content, redness, PPFD, number of days after 2 September 2021, the lowest temperature of the previous night, humidity, leaf thickness and leaf temperature as fixed effects and tree, leaf and date as random effects.

#### Model for the relative number of active PSI centers

The linear mixed effect model for the relative number of active PSI centers tested the effect of leaf thickness, as the number of stacked cells and cell size may obviously affect the number of PSI centers. Leaf temperature was included because it might affect the measurement. The model for the number of active PSI centers thus tested the number of days after 2 September 2021, chlorophyll content, redness, leaf thickness and leaf temperature as fixed effects, and date as a random effect.

#### Model for k_PI_

The dependent variable, *k*_PI_, was calculated as ln(*C*/*I*)/*t*, where *C* and *I* are the *F*_V_/*F*_M_ values measured before and after the illumination treatment, respectively, and *t* is the duration of the treatment (Tyystjärvi and Aro 1996). The linear model for *k*_PI_ tested the effects of leaf type (green, green senescing, yellow, red), redness index, chlorophyll content, light color of the treatment, interactions between redness and light color and redness and chlorophyll content, as well as the interaction between all three.

#### Models for recovery from photoinhibition

The dependent variable, recovery, was calculated as (*R* − *I*)/(*C* − *I*), where *C*, *I* and *R* are *F*_V_/*F*_M_ values measured before the photoinhibition treatment, after the treatment and after the subsequent recovery period, respectively. Recovery from photoinhibition was tested with two different models. First, a linear model was constructed to test whether lincomycin actually prevents photoinhibition in senescent leaves. The model thus tested the effects of light color, chlorophyll content, redness, severity of photoinhibition (defined as *I*/*C*, where *C* and *I* are *F*_V_/*F*_M_ measured before and after the photoinhibition treatments), and the presence of lincomycin. The second model tested recovery from photoinhibition in the absence of lincomycin, with leaf type (green, green senescent, yellow, red), color of light, chlorophyll content and redness of the leaf, severity of photoinhibition and the interaction between chlorophyll content and redness.

A detailed description of each model is shown in Summary of statistics available as Supplementary data at *Tree Physiology* Online.

#### Modeling

In the case of the linear effect mixed models, statistical significance was tested a posteriori with a likelihood ratio test by using analysis of variance to compare a reduced model with the full model, both fitted without applying the residualized maximum likelihood criterion. The linear and linear mixed effect models were constructed in R version 4.2.2. (R Core Team, 2021), using the lme4 library (Bates et al. 2015) and the beta regression model was constructed with the betareg library (Cribari-Neto and Zeileis 2010). The Yeo–Johnson transformation was done with the VGAM library (Yee, 2020) and the optimization of the λ parameter of the transformation was done by testing the quantiles of the transformed variable with the EnvStats library (Millard, 2013).

Student’s *t*-tests (two-tailed, heteroscedastic) for protein data (aspen) and data fitting for pigment calibrations were performed in Microsoft Excel.

Figures were prepared with SigmaPlot (Systat Software Inc., USA) or with R. For the number of individual trees, leaves and leaf sections used in each figure and for descriptive statistics, see Table S1 Summary of statistics available as Supplementary data at *Tree Physiology* Online.

## Results

### Photoinhibition did not differ significantly between red and yellow senescing leaf pieces

**Figure 1:**
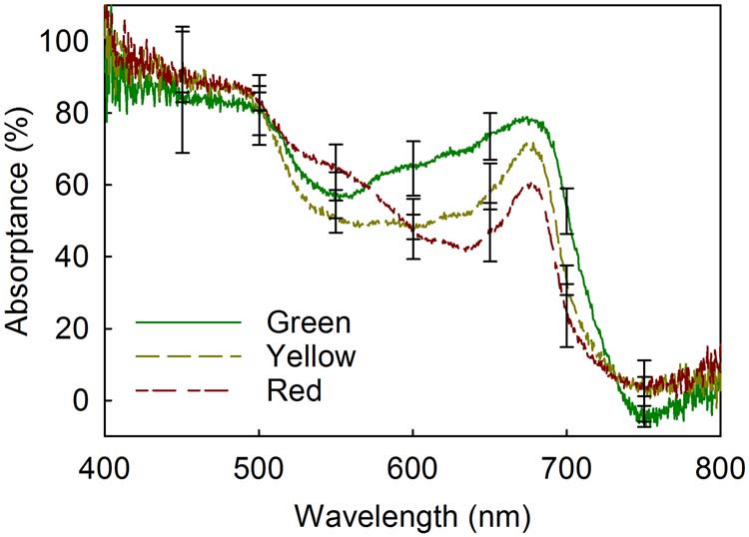
Absorptance of fully green and senescing (yellow or red) maple leaves. Average chlorophyll (*a* + *b*) contents were 31.6 ± 9.1, 8.1 ± 5.0 and 2.3 ± 1.6 μg cm^−2^, for green, yellow and red leaf pieces, respectively. The redness index *R* was 0.096 ± 0.07, 0.12 ± 0.03 and 0.78 ± 0.7 for green, yellow and red leaf pieces, respectively (see Materials and methods for details). Each line represents an average of four biological replicates, and error bars show the standard deviation (SD).

Absorptance was measured from fully green and senescing maple leaves, collected during the autumn from outdoors-grown trees. Red pieces (from senescing leaves) absorbed more green light (~500–600 nm) than yellow leaf pieces, or green pieces obtained from non-senescing leaves (Figure 1). The position of the red edge of the absorptance, in turn, decreased with chlorophyll content (Figure 1).

**Figure 2:**
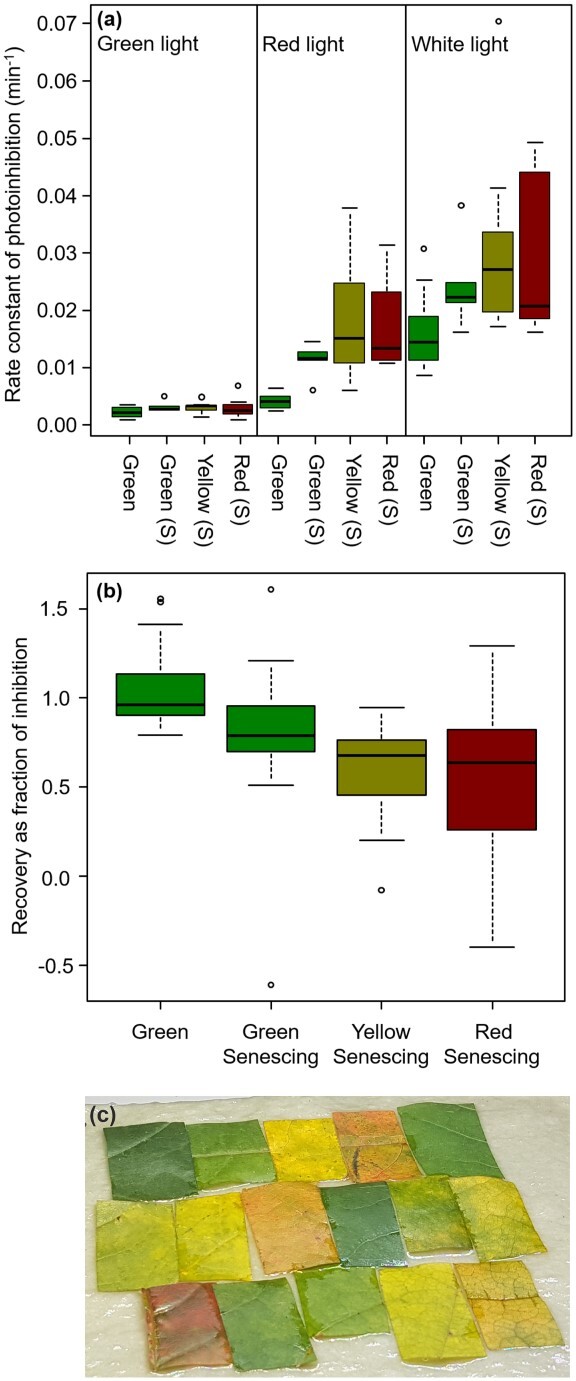
Photoinhibition of PSII in green and senescing maple leaves. (a) Leaf pieces were illuminated (PPFD 2000 μmol m^−2^ s^−1^) with green (500–600 nm), red (λ > 600 nm) or white light in the presence of lincomycin. Rate constants of photoinhibition were then calculated by assuming that the light-induced loss in the *F*_V_/*F*_M_ parameter followed first-order reaction kinetics. (b) Leaf pieces were illuminated in the absence of lincomycin and let to recover over-night under low light and the recovery in the *F*_V_/*F*_M_ parameter value was quantified. Leaf pieces were cut from fully green leaves (green), or from senescing (S) leaves containing green, yellow or pale green (yellow) and red sections (red). The thick line shows the median and the box represents data between the 25th and the 75th percentiles, and the error bars extend either to ±1.5 times the interquantile range or to the maximum or minimum of the data, whichever produces a shorter bar. For the original data, see Figure S2 available as Supplementary data at *Tree Physiology* Online, and for the average chlorophyll contents, see Table S3 available as Supplementary data at *Tree Physiology* Online. (c) An example of a set of leaf pieces used.

To test if red pigments (quantified by their 530-nm absorbance; see Figure S1 available as Supplementary data at *Tree Physiology* Online and Materials and methods for details) protect senescing maple leaves from photoinhibition of PSII, pieces of green and senescing leaves were illuminated with strong light (PPFD 2000 μmol m^−2^ s^−1^). As the absorptance measurements (Figure 1) suggest that maple anthocyanins did not significantly absorb red light, the effects of white, green (500–600 nm) and red (λ > 600 nm) light were compared. In all leaf types, illumination with white light damaged PSII most efficiently and illumination with green light least efficiently (Figure 2a; for the original values, see Figure S2 and Table S2 available as Supplementary data at *Tree Physiology* Online). A linear statistical model for the rate constant of photoinhibition (*k*_PI_) was highly significant (*F*(8,95) = 50.3, *P* < 2.2 × 10^−16^) and showed that *k*_PI_ was significantly affected by light color (coefficient 0.70 ± 0.0095, *P* = 6.75 × 10^−11^) and leaf group (coefficient 0.016 ± 0.0069, *P* = 0.0256) but not by the other variables (see Summary of statistics available as Supplementary data at *Tree Physiology* Online). In particular, illumination with green and white light (where anthocyanins can contribute to light absorption) caused a similar decrease in *F*_V_/*F*_M_ in yellow and red leaf pieces (Figure 2a; Figure S2 available as Supplementary data at *Tree Physiology* Online). The data show that redness did not protect against the damaging reaction of photoinhibition.

When the leaf pieces were allowed to recover in low light after the photoinhibition treatment, the *F*_V_/*F*_M_ value showed some increase even in lincomycin-treated leaves. The efficiency of lincomycin in preventing the recovery from photoinhibition was therefore tested statistically by measuring the extent of recovery as (*R* − *I*)/(*C* − *I*), where *C*, *I* and *R* are the *F*_V_/*F*_M_ values measured in the control, after the inhibition treatment and after the recovery, respectively. A linear statistical model of this parameter was highly significant (*F*(6190) = 36.62; *P* < 2.2 × 10^−16^) and showed that lincomycin inhibited the increase in *F*_V_/*F*_M_ during the recovery period strongly but not completely (effect size −0.32 ± 0.03, *P* < 2 × 10^−16^). Senescence-related parameters including chlorophyll content, redness and their interaction had additional significant effects, and the severity of photoinhibition affected recovery (see Summary of statistics available as Supplementary data at *Tree Physiology* Online for details).

Next, recovery from photoinhibition was studied in samples treated without lincomycin. When the PSII repair cycle was not blocked, green leaf pieces were able to completely restore their PSII activity if allowed to recover overnight under low light, but recovery was incomplete in senescent leaves (Figure 2b; Figure S2 and Table S2 available as Supplementary data at *Tree Physiology* Online). A linear statistical model testing the recovery from photoinhibition was highly significant (*F*(6,88) = 10.54; *P* = 8.65 × 10^−9^) and showed that recovery from photoinhibition was negatively affected by the leaf type, from green non-senescent to red-senescent leaves (effect size −0.11 ± 0.05, *P* = 0.033). The severity of the photoinhibition treatment also affected, with more relative recovery in samples that were less severely inhibited (effect size 0.23 ± 0.11, *P* = 0.0459). The effect of the severity of photoinhibition might be related to the finding that in the absence of lincomycin, similar light treatments caused more severe photoinhibition in senescent than in green leaves (Figure S2 available as Supplementary data at *Tree Physiology* Online). However, removing the severity of photoinhibition from the model did not significantly affect the effect of leaf type (data not shown).

No significant change in the *F*_V_/*F*_M_ parameter was observed in the dark (Figure S2g available as Supplementary data at *Tree Physiology* Online), indicating that the changes observed after light treatments and low-light recovery were caused by the high light in all leaf types.

### Red pigments increased during the last stages of senescence

**Figure 3:**
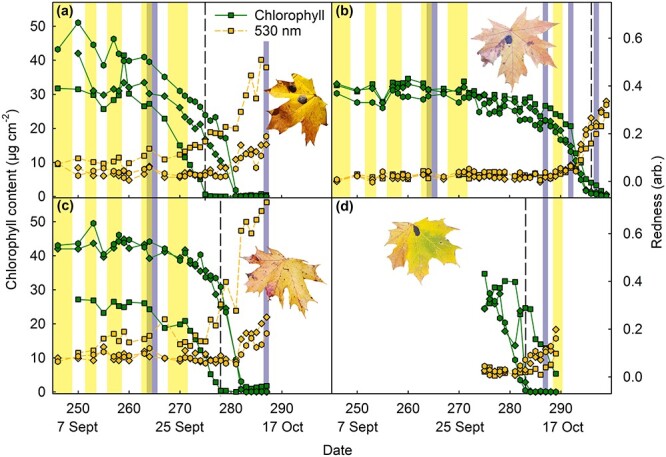
Chlorophyll contents (green symbols, solid line) and the redness index (orange symbols, dashed line), based on 530-nm absorbance, in four maple leaves (a–d) during autumn 2021, until abscission. Three measurement sites per leaf are marked with different symbols (see Figure S4h available as Supplementary data at *Tree Physiology* Online for the positions on the leaf blade). The vertical dashed line highlights the time point at which all chlorophyll was degraded in one of the measurement sites of the leaf. The vertical yellow bars indicate days with high irradiance (daily irradiance > 3000 Wm^−2^, except on 16–17 October ~ 2000 Wm^−2^) and the blue-gray bars indicate days when the previous night had been cold (temperature below 0 °C). The photographs show the leaf after natural abscission. Each leaf belonged to a different tree. For the whole data, see Figures S4–S7 available as Supplementary data at *Tree Physiology* Online.

Next, physiological parameters of several maple leaves were followed in vivo during the course of natural autumn senescence, until abscission (for a summary of the data, see Figure S3 and Boxplots in Summary of statistics available as Supplementary data at *Tree Physiology* Online). In most cases, maple leaves did not senesce uniformly, but a part of the leaf started to lose chlorophyll much, even a week, earlier than other parts (Figure 3; for the whole data, see Figures S4–S7 available as Supplementary data at *Tree Physiology* Online). Also, red pigments appeared unevenly, in both time and space. In general, red pigments appeared when little chlorophyll was left (Figure 3). In a few cases, small amounts of red pigments were present already in the beginning of senescence (see Figures S6 and S7 and Figure S1.4 in Summary of statistics available as Supplementary data at *Tree Physiology* Online), but also in these cases the strongest increase in 530-nm absorbance was observed when most of the chlorophyll had been degraded. In some cases, 530-nm absorbance increased after complete loss of chlorophyll, however, the observation should be treated with caution, as senescing leaves may develop brown (dead) areas while still attached, which may affect the absorbance measurements (Figure S1b available as Supplementary data at *Tree Physiology* Online).

In some cases, a combination of high irradiance with low (previous) night temperature (for the complete weather data, see Figure S8a available as Supplementary data at *Tree Physiology* Online) appeared to coincide with an increase in red pigments (on 21–22 September in Figure 3a and c). However, when all changes in red pigments in senescing leaves were plotted against daily irradiances or night temperatures, no general trend could be seen (Figure S9a and b available as Supplementary data at *Tree Physiology* Online). On the other hand, leaves most shadowed by their neighbors (Figures S4c and f andS7h available as Supplementary data at *Tree Physiology* Online) stayed yellow until all chlorophyll was degraded.

The rate of chlorophyll degradation was initially slow but then increased and all chlorophyll was lost within a week (Figure 3). Although the data obtained from individual leaves shows a biphasic loss of chlorophyll, a boxplot of the dependence of chlorophyll content as a function of Julian day fails to catch the biphasic nature of chlorophyll degradation (Figure 1.3 in Summary of statistics available as Supplementary data at *Tree Physiology* Online). Once initiated at a particular section of the leaf blade, the rate of chlorophyll degradation appeared not to correlate with the presence of red pigments (Figure 3; Figure S9c available as Supplementary data at *Tree Physiology* Online).

### PSII activity was low after cold nights in maple leaves

**Figure 4:**
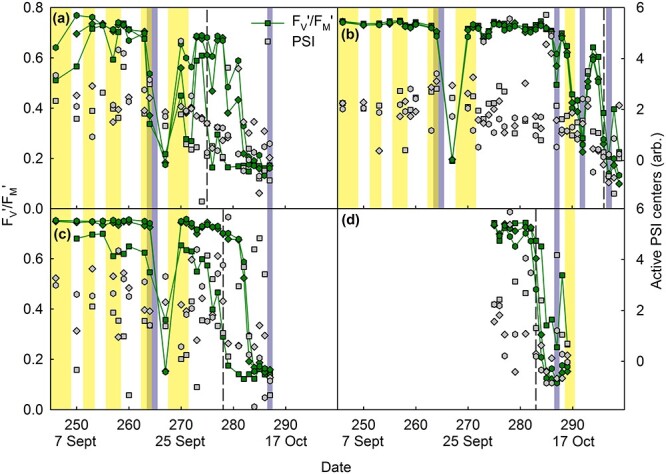
The fluorescence parameter (*F*_M_′ − *F*_0_′)/*F*_M_′ = *F*_V_′/*F*_M_′ (green symbols, solid line), reflecting functional PSII units, and oxidizable P700, measured during a saturating pulse, reflecting active PSI centers (gray symbols; arbitrary units), in four maple leaves (a–d) during autumn 2021, until abscission. Three measurement sites per leaf are marked with different symbols (see Figure S4h available as Supplementary data at *Tree Physiology* Online for the positions on the leaf blade). The vertical dashed line highlights the time point at which all chlorophyll was degraded in one of the measurement sites of the leaf. The vertical yellow bars indicate days with high irradiance (daily irradiance > 3000 Wm^−2^, except on 16–17 October ~ 2000 Wm^−2^) and the blue-gray bars indicate days when the previous night had been cold (temperature below 0 °C). The parameters have been measured from the same leaves as shown in Figure 3. For the whole data, see Figures S10–S13 available as Supplementary data at *Tree Physiology* Online.

In maple leaves the PSII parameter *F*_V_′/*F*_M_′ stayed high throughout the autumn until almost all chlorophyll was degraded (Figures S3a andS10–S13 available as Supplementary data at *Tree Physiology* Online). However, transient decreases in the *F*_V_′/*F*_M_′ value were observed; large drops almost always occurred after cold nights, in both senescing and green leaves (Figure 4; Figure S1.5 in Summary of statistics available as Supplementary data at *Tree Physiology* Online). A combination of low temperature and light was not needed for the decrease in *F*_V_′/*F*_M_′, as on these days, the temperature had already risen above zero by the time the sun rose; only on 19 October did sub-zero temperatures coincide with some amount of sunlight (Figure S8b–e available as Supplementary data at *Tree Physiology* Online). In most cases, *F*_V_′/*F*_M_′ fully recovered within a day (a notable exception was on 21–22 September when two consecutive cold nights occurred, after which the recovery of *F*_V_′/*F*_M_′ took several days).

On the other hand, a parameter reflecting active PSI units did not seem respond to weather conditions but showed a general decrease with leaf chlorophyll content (Figure 4; Figure S3b available as Supplementary data at *Tree Physiology* Online). A linear mixed effect model confirmed the significant dependence of the number of active PSI centers on chlorophyll content (effect size 15.12, *P* < 2.2 × 10^−16^, χ^2^ = 71.1).

### In maple, photochemical activity remained high almost until abscission

**Figure 5:**
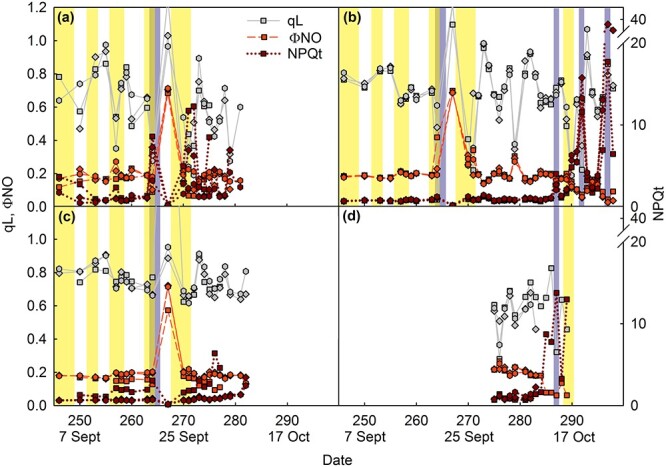
Photochemical quenching (qL; gray symbols, solid line), the yield of unregulated non-photochemical quenching (ΦNO; red symbols, dashed line) and regulated non-photochemical quenching (NPQt; dark red symbols, dotted line) of chlorophyll fluorescence in four maple leaves (a–d) during autumn 2021. Three measurements per leaf were conducted, marked with different symbols (see Figure S4h available as Supplementary data at *Tree Physiology* Online for the positions on the leaf blade). The vertical yellow bars indicate days with high irradiance (daily irradiance > 3000 Wm^−2^, except on 16–17 October ~ 2000 Wm^−2^), and the blue-gray bars indicate days when the previous night had been cold (temperature below 0 °C). Measurements conducted on leaf areas with (almost) no chlorophyll (chlorophyll content less than 0.5 μg cm^−2^) have been excluded. The parameters have been measured from the same leaves, as shown in Figure 3. For the whole data, see Figures S14–S17 available as Supplementary data at *Tree Physiology* Online.

In maple leaves, the qL parameter, reflecting photochemical quenching of chlorophyll fluorescence, stayed remarkably high until almost all chlorophyll was degraded (Figure 5; Figure S3d available as Supplementary data at *Tree Physiology* Online). In fact, qL stayed clearly higher than *F*_V_′/*F*_M_′ or (*F*_M_′ − *F*_S_)/*F*_M_′ (Figure S3a and c available as Supplementary data at *Tree Physiology* Online). Unsurprisingly, qL strongly depended on the incident PPFD (Figure S3d available as Supplementary data at *Tree Physiology* Online), but correlation with the total irradiance of the day was poor (Figure 5; Figures S14–S17 available as Supplementary data at *Tree Physiology* Online).

Two measured parameters of regulated non-photochemical quenching (NPQ) of chlorophyll fluorescence, NPQt (an NPQ parameter that can be measured without dark-acclimation; Tietz et al. 2017) and NPQ yield (ΦNPQ), increased in senescing leaves, especially during bright days (Figure 5; Figure S3e and f available as Supplementary data at *Tree Physiology* Online). A linear mixed effect model confirmed that NPQt significantly increased with decreasing chlorophyll content (size of the effect of chlorophyll content was −0.062, *P* < 2.2 × 10^−16^, χ^2^ = 454.93) and increased with redness (effect size 0.053, *P* = 5.3 × 10^−5^, χ^2^ = 16.4). The effect of PPFD was highly significant but relatively small (effect size 0.0044, *P* < 2.2 × 10^−16^, χ^2^ = 352.0). In addition, leaf temperature had a significant effect (effect size 0.0012, *P* = 0.0012, χ^2^ = 10.5). The yield of unregulated fluorescence quenching (ΦNO), on the other hand, stayed relatively constant during the whole measurement period, though a decrease was observed when only little chlorophyll was left (Figure 5; Figure S3g available as Supplementary data at *Tree Physiology* Online). A peculiar peak in ΦNO, observed after the two cold days (21–22 Sept), may be related to low PSII activity during that time (Figures 4 and 5).

**Figure 6:**
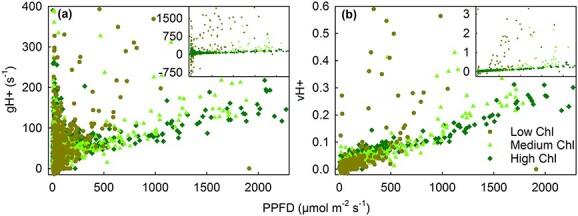
The effect of ambient light PPFD on thylakoid membrane proton conductivity (gH+; a) and steady-state proton flux (vH+; b), measured in vivo from maple leaves in their natural environment, in leaf areas with low (less than 10 μg cm^−2^; olive circles), medium (10–25 μg cm^−2^, light green upward triangles) or high (over 25 μg cm^−2^; green diamonds) chlorophyll (Chl) content. Measurements conducted on leaf areas with very low chlorophyll content (<0.5 μg cm^−2^) have been excluded. The insets show the complete data (excluding fewer than 10 outliers). Each symbol represents an individual measurement.

In line with the high qL values, senescing leaves were able to form a proton gradient over the thylakoid membrane; values for the parameters reflecting proton conductivity (gH+) and steady state proton flux (vH+) were in senescing leaves close to those measured from green leaves, though slightly smaller at low light intensities (Figure 6). Linear mixed effect models for gH+ and vH+ showed that both parameters were positively affected by PPFD (effect size 0.50, *P* = 4.8 × 10^−12^, χ^2^ = 47.76 for gH+ and effect size 1.80, *P* < 2.2 × 10^−16^, χ^2^ = 1516.2 for vH+). Proton conductivity decreased with the number of days after 2 September 2021 (effect size −3.41, *P* = 0.0044, χ^2^ = 8.11). As expected, vH+ depended on gH+ (effect size 0.53, *P* < 2.2 × 10^−16^, χ^2^ = 622.6) but the effects of interaction between gH+ and PPFD as well as the interaction between gH+ and chlorophyll content were negative (effect sizes −0.91, *P* < 2.2 × 10^–16^, χ^2^ = 183.8 and − 0.49, *P* = 8.4 × 10^−7^, χ^2^ = 24.3, respectively). vH+ also depended on chlorophyll content (effect size 0.77, *P* < 2.2 × 10^−16^, χ^2^ = 112.3). Redness had a major positive effect on gH+ (effect size 6.75), but the effect was only barely significant (*P* = 0.047, χ^2^ = 3.96), whereas the effect of redness on vH+ was negative (effect size −0.52, *P* = 7.98 × 10^−11^, χ^2^ = 46.5).

Lastly, the difference between ambient temperature and leaf temperature, reflecting functionality of vasculature and stomata, was found to be comparable in green and senescing leaves (Figure S3h available as Supplementary data at *Tree Physiology* Online).

### In maple leaves, high 530-nm absorbance coincided with low PSII efficiency

**Figure 7:**
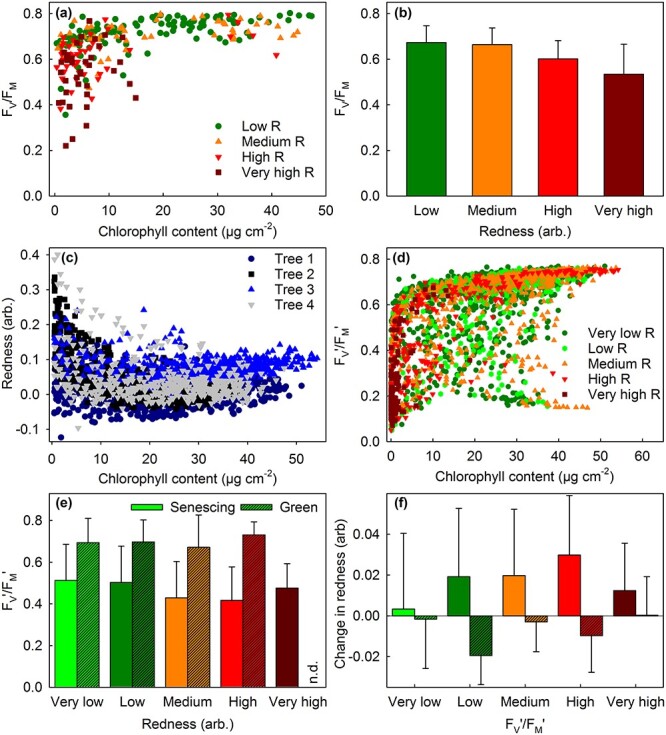
PSII activity in maple leaves with different amounts of red pigments and chlorophylls. (a) *F*_V_/*F*_M_ values of leaf pieces with different (low, medium, high or very high) redness indexes (*R*; estimated based on 530-nm absorbance) plotted against their chlorophyll contents. (b) Average *F*_V_/*F*_M_ values of senescing leaf pieces (chlorophyll (*a* + *b*) content < 10 μg cm^−2^) with different redness indexes. The data (a–b) are from Figure 2. (c) Redness index and chlorophyll content in leaves of four different maple trees. (d) *F*_V_′/*F*_M_′ values of leaf sections with different (very low, low, medium, high or very high) redness indexes plotted against their chlorophyll contents. (e) Average *F*_V_′/*F*_M_′ values of senescing leaf sections (non-hatched bars; chlorophyll (*a* + *b*) content 3–10 μg cm^−2^) and non-senescing leaf sections (hatched bars; chlorophyll (*a* + *b*) content > 30 μg cm^−2^) with different redness indexes. (f) Average changes in redness index (between two subsequent measurement days) in senescing leaf sections (non-hatched bars; chlorophyll (*a* + *b*) content 5–20 μg cm^−2^) and non-senescing leaf sections (hatched bars; chlorophyll (*a* + *b*) content > 30 μg cm^−2^) with different *F*_V_′/*F*_M_′ values (on the first measurement day). The data (c–f) are from Figures S4–S7 and S10–S13 available as Supplementary data at *Tree Physiology* Online. Error bars show SD. In (a), (c) and (d), each symbol represents an individual measurement. See Table S4 available as Supplementary data at *Tree Physiology* Online for the chlorophyll contents.

Next, the amounts of red pigments, chlorophyll contents and PSII values of green and senescing maple leaves were compared in more detail. Leaf sections with high redness indexes tended to have low *F*_V_/*F*_M_ values (measured after 30-min dark-acclimation from detached non-stressed leaves; Figure 7a). As PSII activity decreases with decreasing chlorophyll content of the leaf, average *F*_V_/*F*_M_ values of leaves with different amounts of red pigments were calculated for senescing leaves only (Figure 7b); in this comparison, the average chlorophyll contents of the groups were close to each other (Table S4 available as Supplementary data at *Tree Physiology* Online). A decreasing trend in *F*_V_/*F*_M_ as a function of 530-nm absorbance was found. A linear model of *F*_V_/*F*_M_ was highly significant (*F*(3252) = 113.8; *P* < 2.2 × 10^−16^) and showed that redness lowered the Yeo–Johnson-transformed *F*_V_/*F*_M_ value strongly (coefficient 7.36 ± 0.92), whereas chlorophyll content had a smaller positive effect (5.2 ± 0.69). See the Summary of statistics available as Supplementary data at *Tree Physiology* Online for the full results of the model.

We also investigated the relationship of chlorophyll content and 530-nm absorbance with *F*_V_′/*F*_M_′ values, measured from attached maple leaves during ambient light conditions (Figures 3 and 4; Figures S4–S7 and S10–S13 available as Supplementary data at *Tree Physiology* Online). In maple leaves, as noted already above, red pigments preferentially appeared only during late senescence; in general, when the chlorophyll content had decreased to ~ 10 μg cm^−2^, a clear increase in red pigments was observed (Figure 7c; Figure S1.4 in the Summary of statistics available as Supplementary data at *Tree Physiology* Online). The few exceptions, where a leaf section with high chlorophyll content showed also a high redness index (Figure 7c; Figures S4a,S6b andS7b available as Supplementary data at *Tree Physiology* Online), seemed to coincide with low *F*_V_′/*F*_M_′ values (Figures S10a,S12b and S13b available as Supplementary data at *Tree Physiology* Online).

Expectedly, the *F*_V_′/*F*_M_′ values were slightly lower than the *F*_V_/*F*_M_ values; however, the responses of both parameters to chlorophyll content were very similar (Figure 7a and d). Furthermore, when average *F*_V_′/*F*_M_′ values were calculated for leaf sections with different amounts of red pigments, red senescing leaf sections had lower *F*_V_′/*F*_M_′ values than yellow (low 530-nm absorbance) senescing leaf sections (Figure 7e). In contrast, *F*_V_′/*F*_M_′ values of non-senescing (green) leaves were high regardless the amount of red pigments (Figure 7e). The beta regression model for factors affecting the *F*_V_′/*F*_M_′ value showed that redness index strongly lowered *F*_V_′/*F*_M_′ (coefficient −1.21 ± 0.24). The effect of PPFD was smaller (coefficient − 0.52 ± 0.02), whereas chlorophyll content strongly increased *F*_V_′/*F*_M_′ (coefficient 3.23 ± 0.14), thus confirming the findings based on descriptive statistics (Figure 7). The effect of the lowest temperature of the previous night was large (coefficient 4.05 ± 2.4) but not statistically significant (*P* = 0.09). Furthermore, the number of days passed after 2 September 2021 had a strong negative effect (−2.433 ± 0.20), and weak but significant effects by leaf thickness, humidity, number of open PSI centers, and by interaction between PPFD and chlorophyll content, between leaf temperature and time after 2 September 2021 and between total radiation and the lowest temperature of the previous night were also observed (see Summary of statistics available as Supplementary data at *Tree Physiology* Online).

Next, to test if low PSII efficiency could induce synthesis of red pigments, changes in the amounts of red pigments between two subsequent measurement days were calculated, separately for leaves with high or low *F*_V_′/*F*_M_′ (on the first day). Leaf sections with low *F*_V_′/*F*_M_′ values, regardless of whether they were senescing or not, did not, on the average, synthetize more red pigments than leaf sections with high *F*_V_′/*F*_M_′ values (Figure 7f). To conclude, a causal relationship between low PSII efficiency and red pigments could not be observed. Curiously, leaf sections with high chlorophyll content showed negative values for the change in red pigments (Figure 7c and f; see also Figure S4c available as Supplementary data at *Tree Physiology* Online).

### Red leaves were thicker than yellow ones, and red aspen leaves contained more protein than yellow ones

**Figure 8:**
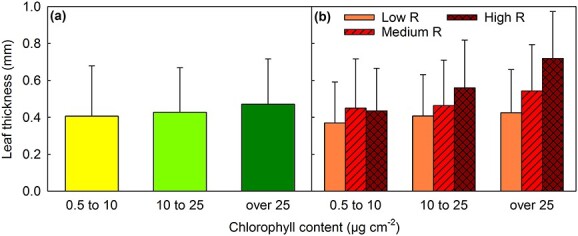
Thickness of green and senescing maple leaves. (a) Average thickness of leaf sections with low (0.5–10 μg cm^−2^), medium (10–25 μg cm^−2^) or high (over 25 μg cm^−2^) chlorophyll (*a* + *b*) contents. (b) Average thickness of leaf sections with low, medium or high chlorophyll content, and low (open bars), medium (hatched bars) or high (cross-hatched bars) redness index (*R*; estimated from 530-nm absorbance). See Table S4 available as Supplementary data at *Tree Physiology* Online for the average chlorophyll values. The error bars show the SD.

Senescing maple leaf sections were found to be thinner than leaf sections with high chlorophyll content (Figure 8a). In addition, leaf sections with low amounts of red pigments were thinner than red sections (Figure 8b; see also Figures S6.4 and S6.5 in Summary of statistics available as Supplementary data at *Tree Physiology* Online). The correlation between leaf thickness and redness was unclear in leaf sections with very low chlorophyll content (≤ 10 μg cm^−2^) but pronounced in sections with high chlorophyll content (Figure 8b). The red senescing leaf sections contained slightly less chlorophyll than yellow sections, and red sections with high chlorophyll content contained more chlorophyll than their non-red counterparts (Table S4 available as Supplementary data at *Tree Physiology* Online). Thus, correlation with chlorophyll content (Figure 8a) may have flattened and enhanced the correlation between redness and thickness in senescing and green leaves, respectively. A linear mixed effect model of leaf thickness revealed that both chlorophyll content and redness index increased thickness but the effect of redness was much stronger (effect size 1.08 ± 0.06 on the transformed thickness variable) than that of chlorophyll content (effect size 0.70 ± 0.08). Leaf temperature had a very small effect (effect size −0.013 ± 0.002). See the Summary of statistics available as Supplementary data at *Tree Physiology* Online for a full summary of the results of the model.

**Figure 9:**
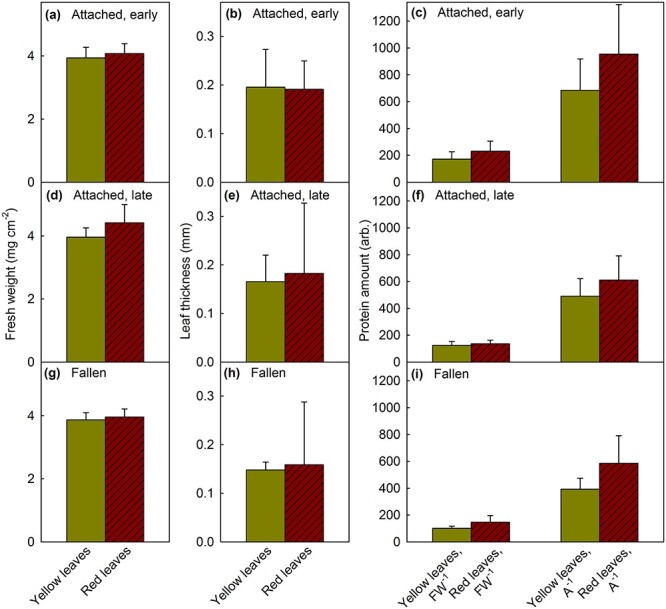
Fresh weight, leaf thickness and protein content of senescent yellow and red aspen leaves. The protein amount was calculated on fresh weight (FW) and leaf area (A) basis. Leaves were collected when they were still weakly attached to a tree (a–f), when the tree was still mostly green (a–c) or when the tree was mostly yellow/red (d–f), or from the ground (g–i). The mean 530-nm absorbance (chlorophyll effect was not corrected for) values of yellow aspen leaves were 0.58–0.62 and those of red leaves 0.82–1.04. Average chlorophyll (SPAD) values of yellow leaves were between −2.55 and −1.21, and those of red leaves were between −0.83 and −0.45. Visually, no chlorophyll could be detected, except for very low amounts in some red leaves. Each bar represents an average calculated from nine leaves, collected from at least three individual trees, and the error bars show the SD. The differences between red and yellow senescing leaves in fresh weight and protein amounts were significant when all the measurements (a–c) were pooled (*P* < 0.026; *t*-test).

Finally, to estimate the effect of red pigments on nutrient resorption, recently fallen aspen leaves, and leaves that were about to fall, were collected. As in maple, red aspen leaves were on the average thicker than yellow aspen leaves (Figure 9), even though the difference was not statistically significant. Red senescing aspen leaves were also heavier than yellow senescing leaves (Figure 9). Accordingly, red leaves contained more proteins than yellow leaves, both on an area and a fresh weight basis (Figure 9).

## Discussion

Based on the previous literature, it seems clear that anthocyanins (or red pigments, as the actual chemical compositions causing the red colors have rarely been determined) can protect young or mature leaves from photoinhibition, at least in some species and in harsh environmental conditions (Gould et al. 2018, Agati et al. 2021, Hughes et al. 2022). The question, whether photoprotection is the (primary) role of anthocyanins also in senescing leaves, has recently been a source for active discussion (Renner and Zohner 2019, 2020, Agati et al. 2021, Pena-Novas and Archetti, 2021*a*, Hughes et al. 2022). A reason for the debate is that results regarding a photoprotective function of red pigments in senescing leaves are contradictory and scarce. To our knowledge, only one study has shown a clear protective effect by anthocyanins in senescing leaves (Hoch et al. 2003; though, the site(s) of the mutation causing the anthocyanin deficiencies and flavonoid compositions of the mutants are not known to us).

A reason for contrasting results (Feild et al. 2001, Hoch et al. 2003, Duan et al. 2014, Pena-Novas and Archetti, 2021*b*) may be that comparisons between anthocyanic and non-anthocyanic leaves are not simple. For example, if the compared leaves belong to different species or varieties or have grown under different light conditions (e.g., Feild et al. 2001, Lee et al. 2003, Duan et al. 2014), it is probable that the anthocyanin content is not the sole difference between the compared leaves. In addition, leaves or species that do not synthesize anthocyanins may have compensatory protection mechanisms; lack of anthocyanins can coincide with a large xanthophyll pool (Logan et al. 2015), high amount of antioxidant enzymes (Landi et al. 2013) or different gene regulation during senescence (Vangelisti et al. 2020). It has been hypothesized that different senescence strategies or environmental conditions would favor different protection mechanisms (Moy et al. 2015, Hughes et al. 2022). A protective effect by anthocyanins may thus remain unnoticed. On the other hand, a growth condition inducing anthocyanin synthesis (e.g., high light) may also induce other protective mechanisms and an observed (protective) effect may not be related to anthocyanins. In the present study, we have tried to minimize the above-mentioned difficulties by comparing yellow and red sections within one and the same leaf (Figure 2), or yellow and red leaves collected from one and the same tree (Figure 9).

Another important factor to consider is chlorophyll content; leaves with low chlorophyll content (such as senescing leaves) are more vulnerable to PSII damage than leaves with high chlorophyll content (Pätsikkä et al. 2002, Manetas and Buschmann 2011, Havurinne et al. 2022, Mattila et al. 2021, Serôdio and Campbell 2021) because the amount of light absorbed per chloroplast decreases with increasing chlorophyll content. This is especially important in the case of senescing leaves, as anthocyanins often gradually increase while chlorophyll is degraded (e.g., Hoch et al. 2003; Figure 7). In addition, the chlorophyll content of a (senescing) leaf is a strong determinant of also other parameters, such as PSII activity, PSI amount, NPQ induction, carotenoid amounts, leaf thickness and even absorption of green (530 nm) light (Figures 7 and 8; Figures S1,S3 and S18 available as Supplementary data at *Tree Physiology* Online; for statistical evidence, see Summary of statistics available as Supplementary data at *Tree Physiology* Online). Therefore, chlorophyll content should be taken into account when yellow and red senescing leaves are compared. Here, chlorophyll content was always measured and taken into account in statistical modeling; for descriptive statistics we aimed at comparing only leaves with similar chlorophyll contents (Tables S1–S4 available as Supplementary data at *Tree Physiology* Online).

### Can anthocyanins enhance carbohydrate disposal in senescing maple leaves?

White light caused more photoinhibition than red or green light, in both green and senescing leaves (Figure 2). The result agrees with previously measured action spectra of photoinhibition, obtained with several laboratory-grown plant species (for a review, see Zavafer et al. 2015), considering that the white light source used here (a sunlight simulator) emits blue light and also some UV-A radiation (Havurinne et al. 2022). Green light caused very little photoinhibition in all leaf types, indicating that if anthocyanins are to protect PSII, then the crucial factor is their capacity to absorb blue light (Manetas, 2006, Agati et al. 2021). Here, absorptance of blue wavelengths (λ < 500 nm) was close to 100% in both green and senescing maple leaves (Figure 1) and, thus, the present data do not allow us to judge whether red pigments significantly absorbed blue light. Cyanidin-3-glucoside and cyanidin-3-rutinoside, two common anthocyanins in maple (Ji et al. 1992, Schmitzer et al. 2009), show the highest absorbance (in a mixture of water, formic acid and acetonitrile) at ~ 520 nm but the spectra extend to 350 nm (Vergara et al. 2009).

We did not find significant differences in photoinhibition between red and yellow senescing leaf sections of Norway maple (Figure 2; see also Summary of statistics available as Supplementary data at *Tree Physiology* Online). Furthermore, red senescing leaves showed lower *F*_V_/*F*_M_ and *F*_V_′/*F*_M_′ values (Figure 7), an observation reported also earlier (e.g., Kytridis et al. 2008, Nikiforou and Manetas 2010, Nikiforou et al. 2011). Indeed, in Norway maple, red pigments often appeared only when a large part of chlorophyll had already been degraded (Figure 7c). However, if the primary function of anthocyanins were to protect senescing leaves, one would expect that anthocyanins would start to accumulate at the beginning of chlorophyll degradation. Thus, if anthocyanins protect, they protect only at a late stage of senescence. Furthermore, the finding that the repair of PSII during and after a strong light treatment was equally inefficient in red, green and yellow sections of senescent maple leaves (Figure 2b; Figure S2, Table S2 available as Supplementary data at *Tree Physiology* Online; see also Summary of statistics available as Supplementary data at *Tree Physiology* Online) indicates that anthocyanins do not enhance repair of PSII. The results are in agreement with the previous observation that anthocyanin accumulation does not significantly lower light absorption by chlorophylls in sugar maple (van den Berg et al. 2009).

As discussed by Hughes et al. (2022) and above, a small (or non-existing) difference in photoinhibition susceptibility between red and yellow senescing leaves cannot be taken as an absolute proof for the inefficiency of anthocyanins to protect, as yellow leaves may have different, compensatory protection mechanisms. For example, senescing peduncles of common elderberry (*Sambucus canadensis*) rely either on NPQ or anthocyanins, depending on their growth light history (Cooney et al. 2018). Young leaves of *Castanopsis chinensis* and *Acmena acuminatissima* also induce either NPQ or anthocyanin synthesis depending on the season, but in a contrasting way (Yu et al. 2021). In addition, while mutant plants lacking anthocyanins were susceptible to high light stress, yellow senescing birch was as tolerant as the anthocyanin-containing wild types of *C. sericea*, *Vaccinium elliottii* Chapmn. and *Viburnum sargentii* Koehne (Hoch et al. 2003). We did not, however, observe an inverse relationship between NPQ induction and anthocyanin accumulation in maple but, instead, red leaf sections induced more NPQ than yellow sections (Figure S18a and b available as Supplementary data at *Tree Physiology* Online; see also Summary of statistics available as Supplementary data at *Tree Physiology* Online). Besides NPQ, other photoprotection mechanisms exist and, for example, the carotenoid to chlorophyll ratio commonly increases during senescence (e.g., Junker and Ensminger 2016). We also observed the increase, occurring because chlorophylls were degraded more rapidly than carotenoids (Figure S18c available as Supplementary data at *Tree Physiology* Online). Again, however, red leaves contained rather more carotenoids than yellow leaves, although the difference was not statistically significant (Figure S18c available as Supplementary data at *Tree Physiology* Online). To summarize, anthocyanin accumulation did not render other protective mechanisms unnecessary.

Regarding NPQ, Junker and Ensminger (2016) observed opposite results: a decrease in NPQ and an increase in NO in senescing sugar maple leaves. The discrepancy with our results may reflect a difference in the method of estimation of NPQ or the fact that the senescing leaves used by Junker and Ensminger (2016) had lost almost all chlorophyll.

Red senescing maple leaf sections also did not live longer than yellow sections (Figure S9c available as Supplementary data at *Tree Physiology* Online). In aspen, protein amount at abscission was higher in red than in yellow leaves (Figure 9), suggesting that red leaves had problems with nitrogen resorption. Therefore, in the present study, anthocyanin accumulation did not increase the fitness of the tree by increasing carbon gain or nutrient resorption. On the contrary, high NPQ and high amount of carotenoids suggest that red leaf sections were under stronger stress than yellow leaf sections. Indeed, red senescing leaf sections exhibited lower PSII activities than yellow sections (Figure 7; see also Summary of statistics available as Supplementary data at *Tree Physiology* Online). A higher proton conductivity of thylakoid membranes of red leaves, measured with the gH+ parameter, may suggest that redness is associated with membrane leakiness (see Summary of statistics available as Supplementary data at *Tree Physiology* Online). Red light caused a similar amount of PSII damage in red and yellow sections of senescing maple leaves, whereas in white light, yellow sections were, on average, damaged slightly faster than red sections (Figure 2). This finding may suggest that the PSII units in the red sections were intrinsically more vulnerable to PSII damage but anthocyanins offered some photoprotection so that the resulting rate of photodamage was similar when white light was used to induce photoinhibition.

We attempted to test whether low PSII activity induces anthocyanin synthesis. However, a low *F*_V_′/*F*_M_′ value did not predict high anthocyanin synthesis during the following day (Figure 7f) or 2 days (data not shown). The result suggests that the same conditions cause both low PSII activity and anthocyanin accumulation. The findings that anthocyanic leaf sections (Figure 8b) and leaves (Figure 9; see also Summary of statistics available as Supplementary data at *Tree Physiology* Online) are thicker and that anthocyanic leaves contain more protein than non-anthocyanic leaves (Figure 9) suggest that problems in translocation of photosynthates induces anthocyanin synthesis. Here, increased leaf thickness and leaf mass (per area), common responses to nitrogen deficiency, correlated with redness (Figures 8 and 9) and have earlier been found to correlate with high amounts of anthocyanins (Hughes et al. 2022). Problems in translocation would also induce NPQ and contribute to the stress symptoms found in red leaf sections (Figure 7; Figure S18 available as Supplementary data at *Tree Physiology* Online).

We suggest that anthocyanin accumulation is a stress reaction and propose that during senescence, when carbon skeletons are no longer required, nitrogen is actively transported from the leaves and chloroplasts are being degraded, anthocyanin synthesis may replace starch synthesis as a carbohydrate storage mechanism. As anthocyanins contain only carbon, oxygen and hydrogen, their synthesis would help, by disposing of carbohydrates, the light reactions to continue to produce energy required for nutrient resorption right before abscission. The carbohydrate disposal hypothesis is also supported by the fact that red leaves are often found in exposed parts of the Norway maple canopy (personal experience) or under high light (Duan et al. 2014), where carbohydrates would be produced in abundance. High light could also contribute to the observed stress symptoms in red senescing leaves, independently of the proposed translocation problems. However, disruption of phloem export by stem girdling or by cooling induces anthocyanin synthesis (Murakami et al. 2008, Schaberg et al. 2017), indicating that high sugar content may induce anthocyanin synthesis even in the absence of high light. If carbohydrate disposal is the main function of anthocyanin synthesis, then leaf-to-leaf and within-leaf variation in anthocyanin synthesis would be caused by variation in sink source relationships or in differences in the functionality of vasculature in a senescing leaf.

Our suggestion about the role of anthocyanin synthesis in senescence is related to an earlier suggestion of anthocyanin synthesis functioning as an energy escape valve that would also protect the photosynthetic apparatus from light stress, by enabling fast electron transfer via prevention of over-reduction of the photosynthetic electron transport chain when sinks diminish (Lo Piccolo et al. 2018, Hernández and Van Breusegem 2010, Soubeyrand et al. 2018). Protection against light stress was not observed here, suggesting that photoprotection is not the primary function of anthocyanins in senescing maple leaves. It is also possible that anthocyanins serve multiple functions in senescing leaves. More research is needed with deciduous plants, especially at the molecular level, to fully understand the roles of anthocyanins in senescence. For example, possible within-leaf differences in nitrogen content of senescing leaf have not been, to our knowledge, investigated.

## Supplementary Material

Mattila2022_SI_v4_tpad010

## Data Availability

Data are at https://data.mendeley.com/datasets/thjgzhv928/1.
