# Supplementary material for: Red pigments in autumn leaves of Norway maple do not offer significant photoprotection but coincide with stress symptoms
Source: Tree Physiol. 2023 Jan 30;43(5):751–68. doi: 10.1093/treephys/tpad010 (PMC10177003; doi:10.1093/treephys/tpad010)
Supplement: Mattila2022_SI_v4_tpad010 [file mattila2022_si_v4_tpad010.zip › Mattila2022_SI_v4_tpad010.docx]

Supplementary Information

**Red pigments in autumn leaves of Norway maple do not offer significant photoprotection but coincide with stress symptoms**

Heta Mattila, Esa Tyystjärvi

Molecular Plant Biology/ Department of Life Technologies, University of Turku, 20014 Turku, Finland

Corresponding author contact information: esatyy@utu.fi, +358 29 450 4202

**Supplementary figures:**

Supplementary Fig. S1. Calibration of pigment measurements with maple leaves.

Supplementary Fig. S2. Photoinhibition in green, yellow and red maple leaf pieces.

Supplementary Fig. S3. Parameters of photosynthetic electron transfer chain from maple leaf areas with low, medium or high chlorophyll content.

Supplementary Fig. S4. Chlorophyll contents and the redness index in maple leaves during the autumn 2021, until abscission.

Supplementary Fig. S5. Chlorophyll contents and the redness index in maple leaves during the autumn 2021, until abscission.

Supplementary Fig. S6. Chlorophyll contents and the redness index in maple leaves during the autumn 2021, until abscission.

Supplementary Fig. S7. Chlorophyll contents and the redness index in maple leaves during the autumn 2021, until abscission.

Supplementary Fig. S8. Autumn weather in Turku region.

Supplementary Fig. S9. Synthesis of red pigments and degradation of chlorophyll in senescing maple leaves.

Supplementary Fig. S10. PSII and PSI parameters in maple leaves during the autumn 2021, until abscission.

Supplementary Fig. S11. PSII and PSI parameters in maple leaves during the autumn 2021, until abscission.

Supplementary Fig. S12. PSII and PSI parameters in maple leaves during the autumn 2021, until abscission.

Supplementary Fig. S13. PSII and PSI parameters in maple leaves during the autumn 2021, until abscission.

Supplementary Fig. S14. Photochemical and non-photochemical quenching of fluorescence in maple leaves during the autumn 2021, until abscission.

Supplementary Fig. S15. Photochemical and non-photochemical quenching of fluorescence in maple leaves during the autumn 2021, until abscission.

Supplementary Fig. S16. Photochemical and non-photochemical quenching of fluorescence in maple leaves during the autumn 2021, until abscission.

Supplementary Fig. S17. Photochemical and non-photochemical quenching of fluorescence in maple leaves during the autumn 2021, until abscission.

Supplementary Fig. S18. NPQ and carotenoids in maple leaves with different amounts of red pigments.

**Supplementary tables:**

Supplementary Table S1. Number of leaf sections (sites), leaves and individual trees and the N and statistical tests used in the analyses shown in the figures.

Supplementary Table S2. Quantification of photoinhibition and recovery in green and senescing maple leaves (Fig. 2).

Supplementary Table S3. Average chlorophyll contents and redness indexes, at the beginning of the experiments, of leaf pieces used in photoinhibition experiments (Fig. 2).

Supplementary Table S4. Average chlorophyll contents of leaves or leaf pieces, with different redness indexes or F_V_’/F_M_’ values, of the indicated figures.

**Summary of statistics**

**Supplementary figures**


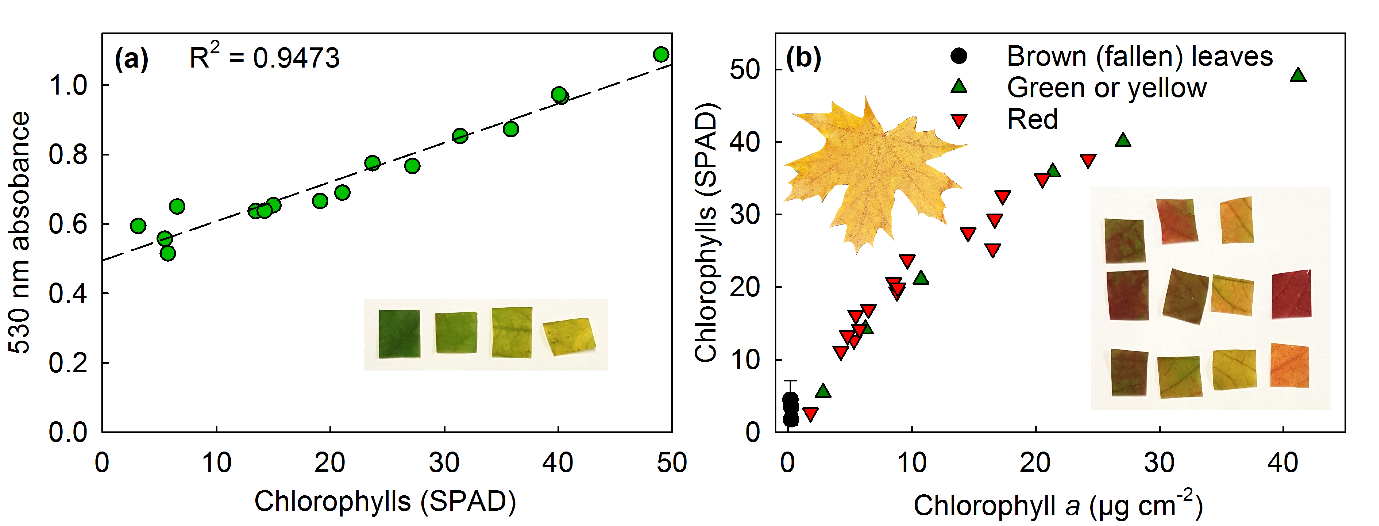


Supplementary Fig. S1. Calibration of pigment measurements with maple leaves. (a) The effect of leaf chlorophyll content (obtained with an optical SPAD method) on light absorbance at 530 nm. Measurements are from leaf pieces that did not contain red pigments (estimated by visual inspection). The dashed line (530 nm absorbance = 0.0113 + Chlorophyll (SPAD) + 0.495) was calculated with linear regression and used to calculate the redness index R. (b) The effect of leaf colour on chlorophyll measurements. Chlorophyll contents were measured both with an optical method (SPAD) and spectroscopically after extraction in dimethylformamide from pieces of maple leaves with low (green or yellow, R < 0.05; upward triangles) or medium to high (red, 0.14 < R < 0.68; downward triangles) redness index. Coloured symbols represent single measurements (leaves collected while attached to the tree). Solid circles represent measurements conducted on brown leaves fallen on the ground (error bars, shown when larger than the symbol, show standard deviations (SD) calculated based on three biological replicates). Chlorophyll *b* contents of brown leaves were too low for reliable quantification and therefore only amounts of chlorophyll *a* are shown. The photographs show examples of leaves and leaf pieces.


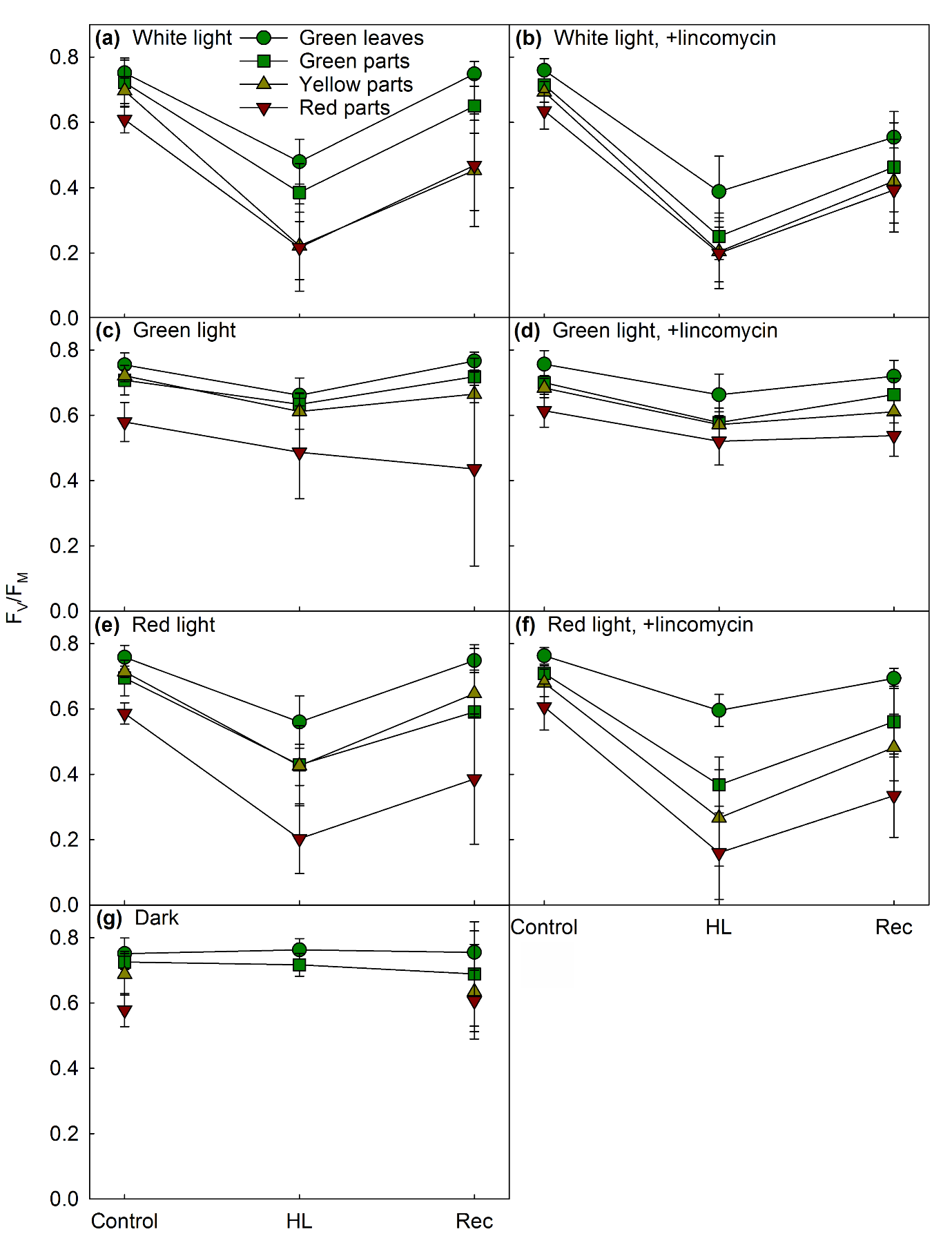


Supplementary Fig. S2. Photoinhibition of PSII in green and senescing maple leaves. Leaf pieces were illuminated (PPFD 2000 µmol m^-2^ s^-1^) at 20 °C with white light for 1 h (a) or 45 min (b), with green light (500‒600 nm) for 2 (c) or 1 h (d), with red light (λ > 600 nm) for 2 (e) or 1 h (f) or incubated in darkness (g), in the absence (a, c, e, g) or presence (b, d, f) of lincomycin. After the treatments, leaf pieces were let to recover over-night under low light. The fluorescence parameter F_V_/F_M_ was recorded before (Control) and after the illumination (HL), and after the over-night incubation (Rec), always after at least 30 min of dark-acclimation. Each symbol represents an average of at least four biological replicates and the error bars show SD. Leaf pieces were cut from fully green leaves (Green leaves), or from senescing leaves containing green (Green parts), yellow or pale green (Yellow parts) and red sections (Red parts). For the average chlorophyll contents, see Supplementary Table S3.


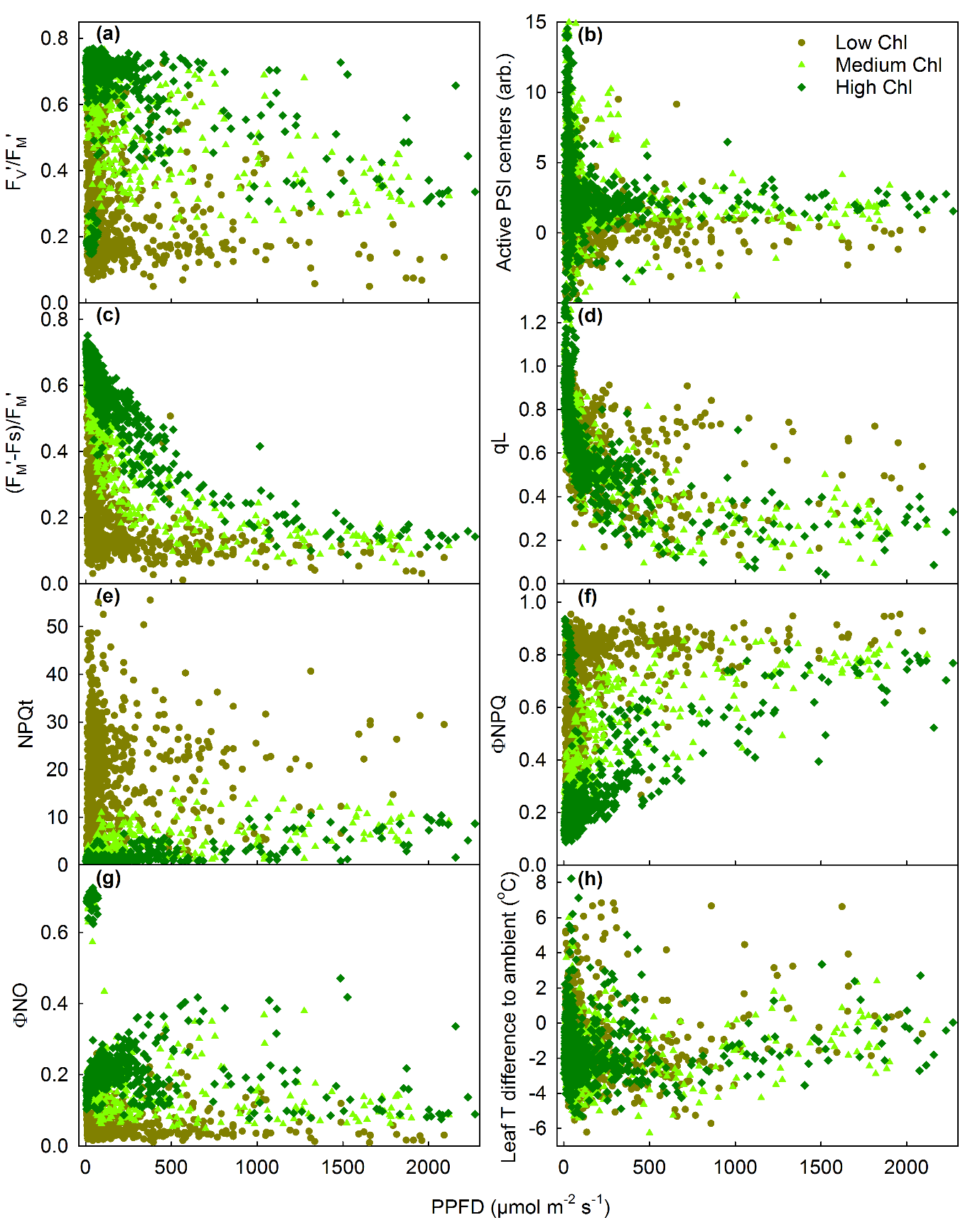


Supplementary Fig. S3. Parameters of photosynthetic electron transfer chain measured from maple leaf sections with low (less than 10 µg cm^-2^; olive circles), medium (10–25 µg cm^-2^; light green upward triangles) or high (over 25 µg cm^-2^; green diamonds) chlorophyll (Chl) content. The measurements were done *in vivo* with MultispeQ from 27 leaves (six sites/leaf), belonging to four trees, on 3 Sep 2021‒26 Oct 2021 under simulated ambient light and ambient temperature. (a) F_V_’/F_M_’, (b) the amount of active PSI centres (arbitrary units), (c) (F_M_’-F_S_)/F_M_’, where F_S_ is fluorescence level under simulated incident light, (d) photochemical quenching qL, calculated as (F_M_’-F_S_)/(F_M_’-F_0_′)*(F_0_′/F_S_), (e) non-photochemical quenching of fluorescence, NPQt, calculated as (4.88/((F_M_′/F_0_′)-1))-1, (f) yield of NPQ, (g) yield of non-regulated fluorescence quenching, ΦNO, calculated as 1/(NPQt+1+qL*4.88) and (h) differences between leaf temperature and ambient temperature were plotted against incident PPFD during the measurement. Each symbol represents an individual measurement. Outliers (< 10 % of the data) are in some cases left out of the figures for clarity.

**
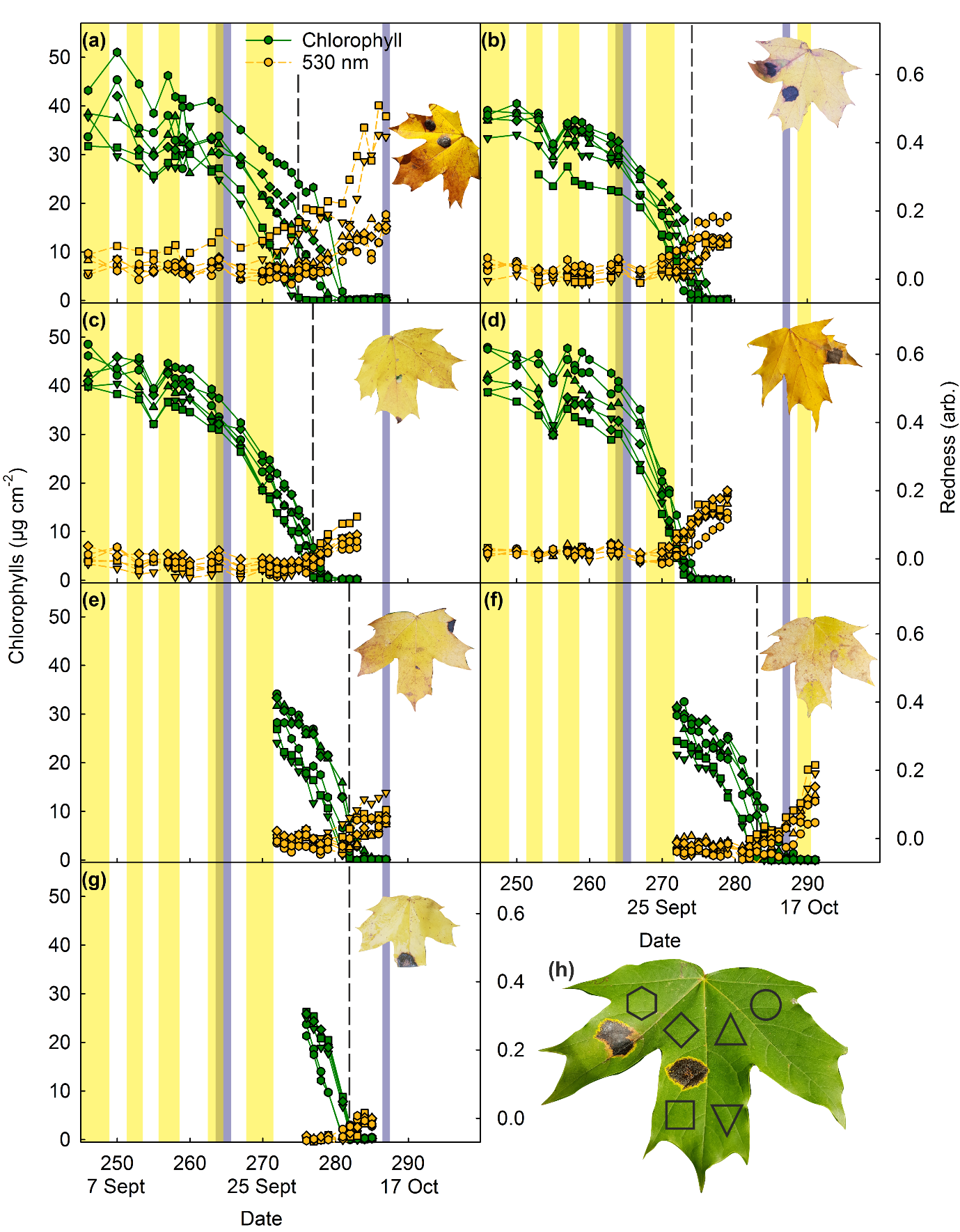
**

Supplementary Fig. S4. Chlorophyll contents (green symbols, solid line) and the redness index (orange symbols, dashed line), based on 530 nm absorbance, in seven leaves (a‒g) of a maple tree during autumn 2021, until abscission. Six measurement sites per leaf are marked with different symbols (h). Chlorophyll content was measured with an optical method and converted to µg cm^-2^ with a calibration curve. The vertical dashed line highlights the time point at which all chlorophyll was degraded in one of the measurement sites of the leaf. The vertical yellow bars indicate days with high irradiance (daily irradiance > 3000 Wm^-2^, except on 16‒17 Oct ~2000 Wm^-2^) and the blue bars indicate days when the previous night had been cold (temperature below 0 °C). Weather data were measured by Finnish meteorological institute. The photographs show the leaf after natural abscission.

**
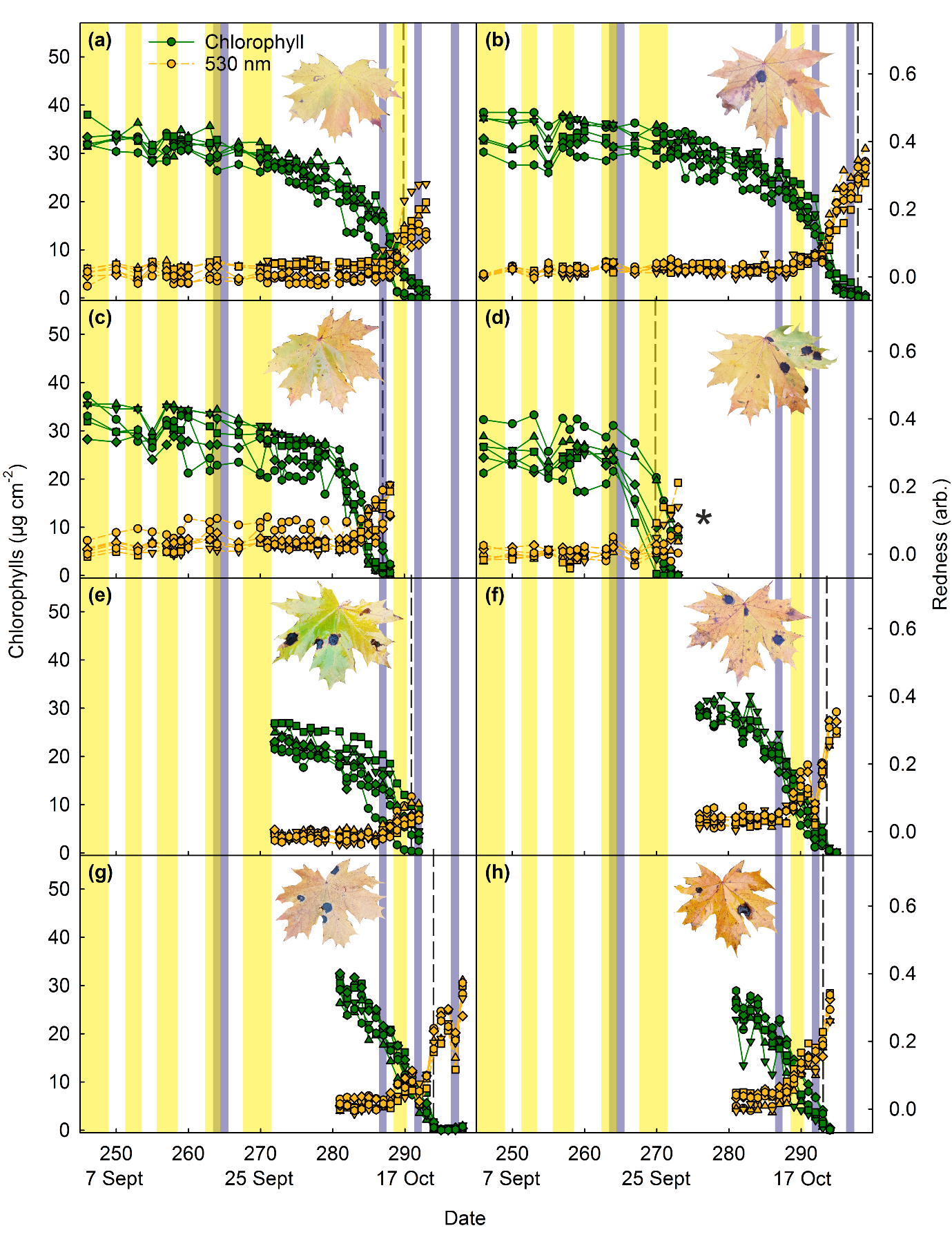
**

Supplementary Fig. S5. Chlorophyll contents (green symbols, solid line) and the redness index (orange symbols, dashed line), based on 530 nm absorbance, in eight leaves (a‒h) of a maple tree during autumn 2021, until abscission. Six measurement sites per leaf are marked with different symbols (see Supplementary Fig. S4h for the positions on the leaf blade). Chlorophyll content was measured with an optical method and converted to µg cm^-2^ with a calibration curve. The vertical dashed line highlights the time point at which all chlorophyll was degraded in one of the measurement sites of the leaf. The vertical yellow bars indicate days with high irradiance (daily irradiance > 3000 Wm^-2^, except on 16‒17 Oct ~2000 Wm^-2^) and the blue bars indicate days when the previous night had been cold (temperature below 0 °C). Weather data were measured by Finnish meteorological institute. The photographs show the leaf after natural abscission. The asterisk highlights a case where the leaf petiole was cut (due to an unknown reason) and the leaf dropped prematurely.

**
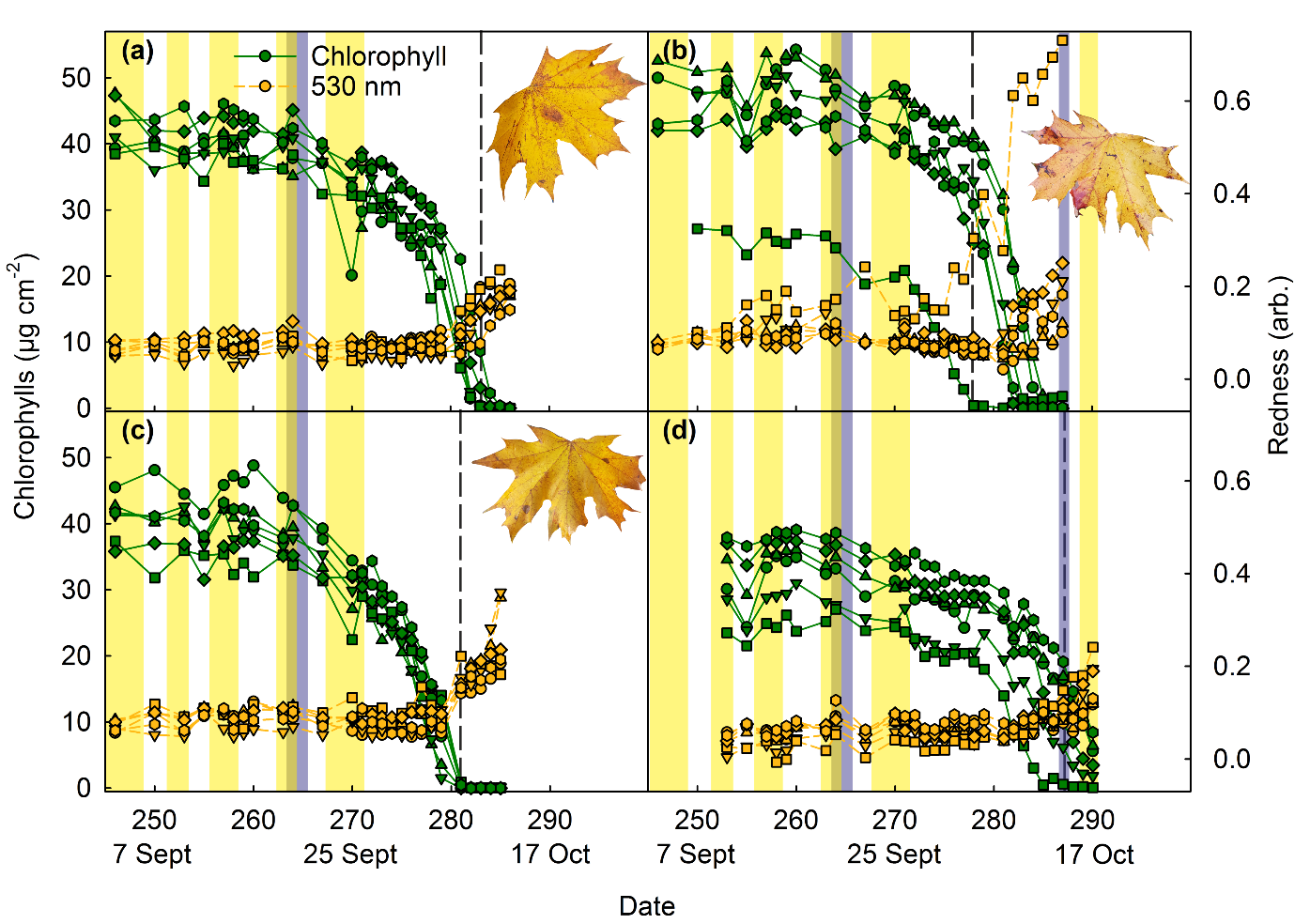
**

Supplementary Fig. S6. Chlorophyll contents (green symbols, solid line) and the redness index (orange symbols, dashed line), based on 530 nm absorbance, in four leaves (a‒d) of a maple tree during autumn 2021, until abscission. Six measurement sites per leaf are marked with different symbols (see Supplementary Fig. S4h for the positions on the leaf blade). Chlorophyll content was measured with an optical method and converted to µg cm^-2^ with a calibration curve. The vertical dashed line highlights the time point at which all chlorophyll was degraded in one of the measurement sites of the leaf. The vertical yellow bars indicate days with high irradiance (daily irradiance > 3000 Wm^-2^, except on 16‒17 Oct ~2000 Wm^-2^) and the blue bars indicate days when the previous night had been cold (temperature below 0 °C). Weather data were measured by Finnish meteorological institute. The photographs show the leaf after natural abscission.

**
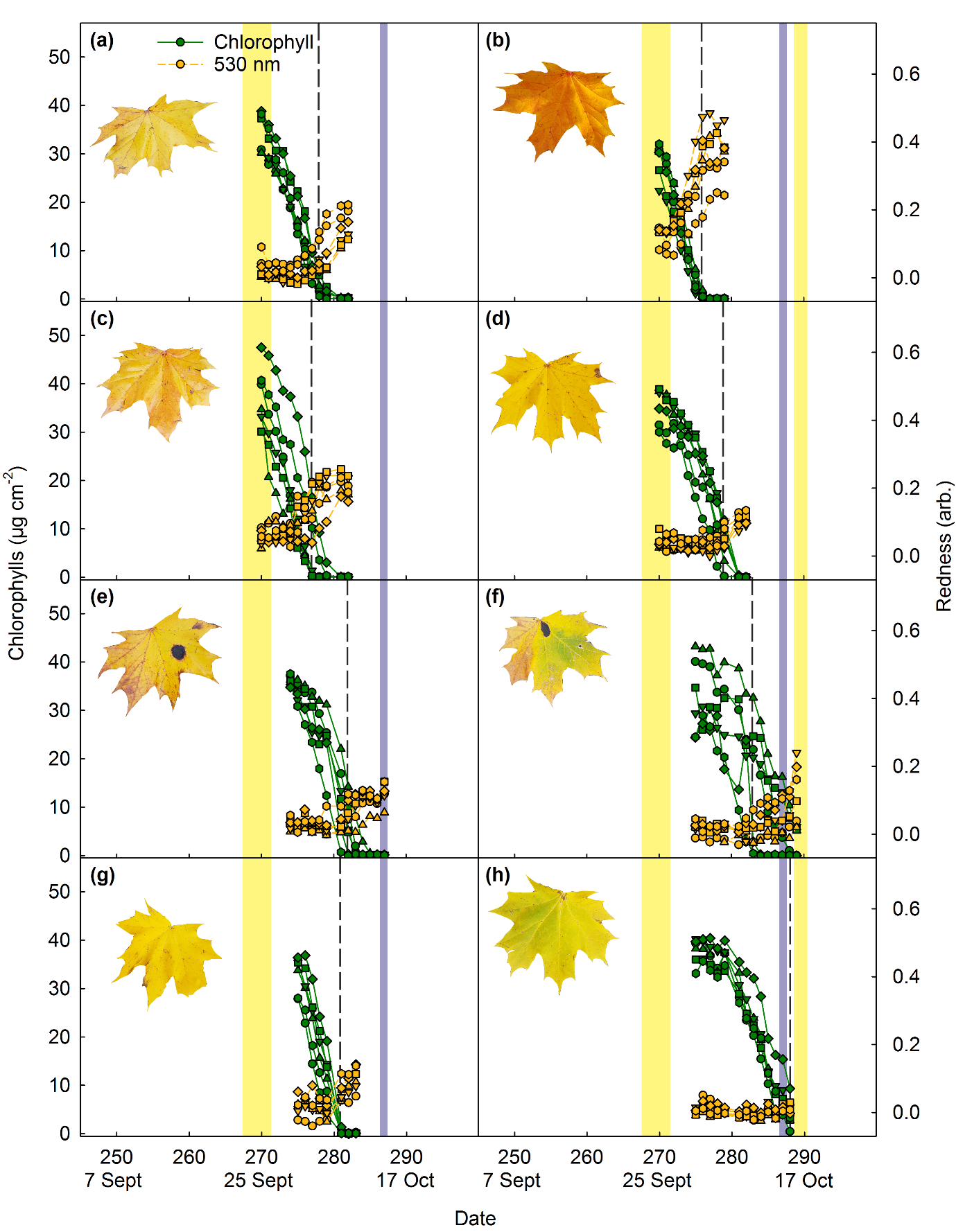
**

Supplementary Fig. S7. Chlorophyll contents (green symbols, solid line) and the redness index (orange symbols, dashed line), based on 530 nm absorbance, in eight leaves (a‒h) of a maple tree during autumn 2021, until abscission. Six measurement sites per leaf are marked with different symbols (see Supplementary Fig. S4h for the positions on the leaf blade). Chlorophyll content was measured with an optical method and converted to µg cm^-2^ with a calibration curve. The vertical dashed line highlights the time point at which all chlorophyll was degraded in one of the measurement sites of the leaf. The vertical yellow bars indicate days with high irradiance (daily irradiance > 3000 Wm^-2^, except on 16‒17 Oct ~2000 Wm^-2^) and the blue bars indicate days when the previous night had been cold (temperature below 0 °C). Weather data were measured by Finnish meteorological institute. The photographs show the leaf after natural abscission.


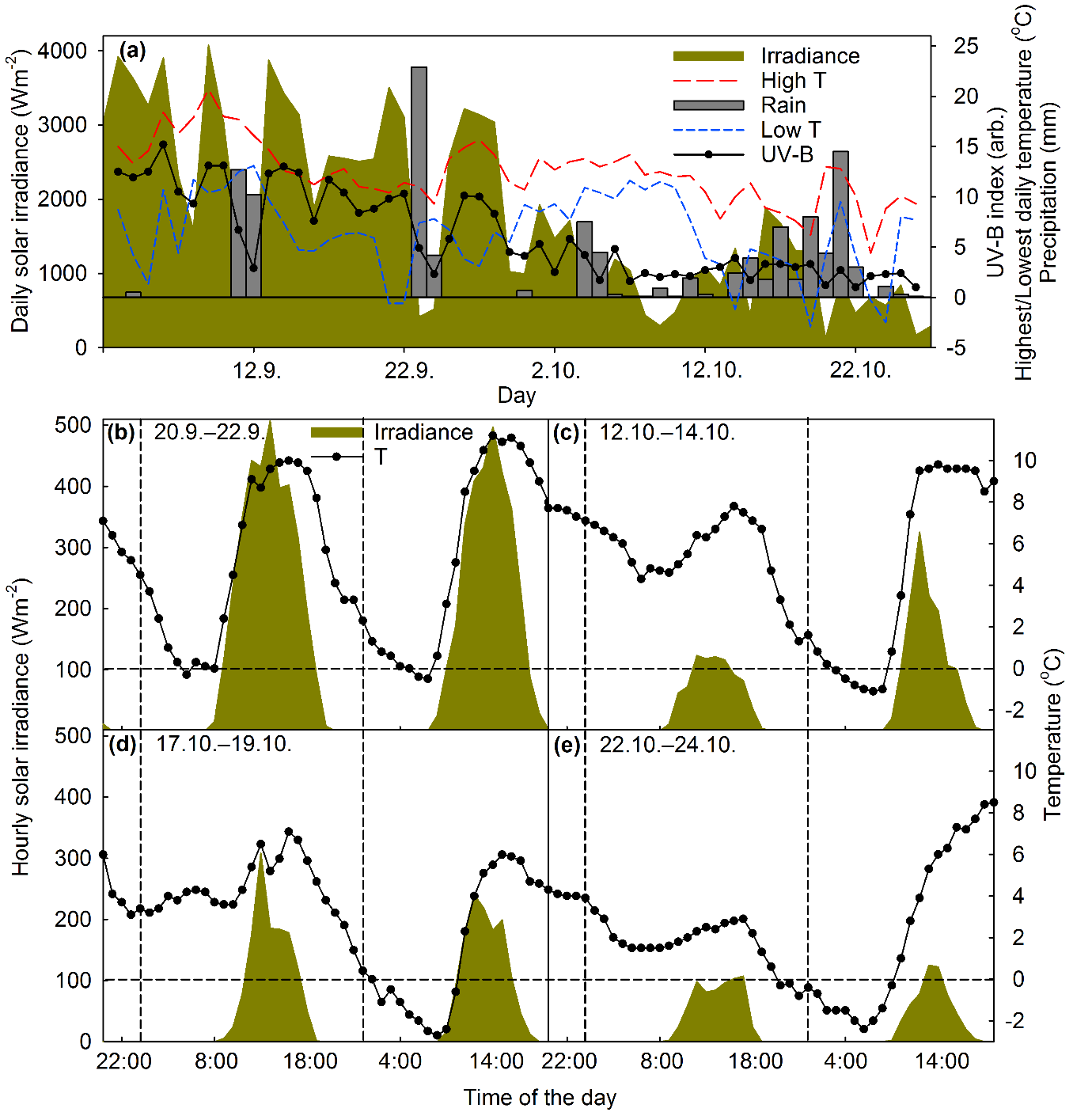


Supplementary Fig. S8. Weather during autumn 2021 in Turku region. (a) The sum of daily solar irradiance (Wm^-2^), highest (High T) and lowest (Low T) daily air temperature and daily precipitation (rain) on 2 Sept 2021‒27 Oct 2021. (b‒e) Hourly values for solar irradiance (Wm^-2^) and air temperature (T) for the indicated days during which temperature decreased below zero (highlighted by the horizontal dashed lines). The vertical dashed lines mark midnight. The parameters were measured by Finnish meteorological institute, in Artukainen (8 km from the measurement sites), except the UV-B index (arbitrary units) which was measured in Jokioinen (85 km from the measurement sites).


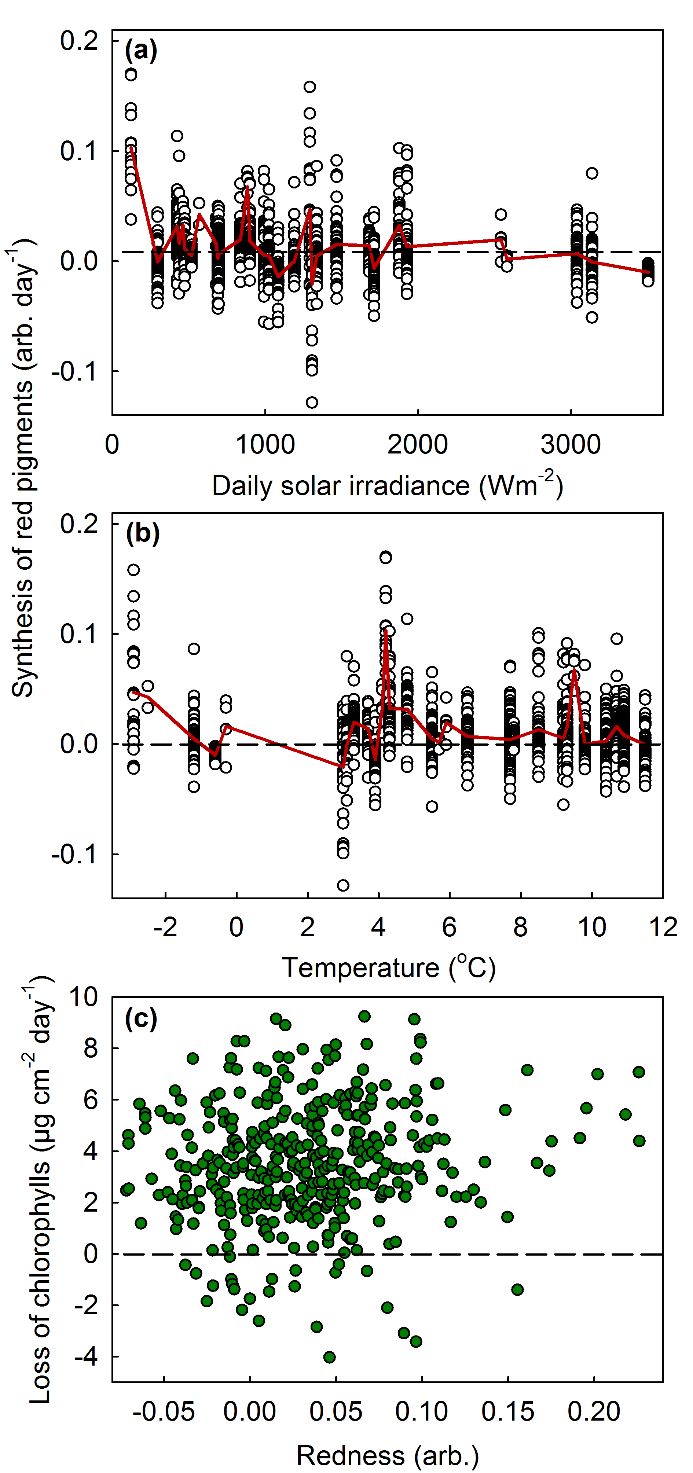


Supplementary Fig. S9. Synthesis of red pigments and degradation of chlorophyll in senescing maple leaves. Effects of the sum of daily irradiance (Wm^-2^; a) and lowest temperature of the day (b) on the increase of the redness index (synthesis of red pigments; based on 530 nm absorbance) in 24 h. The red continuous lines show averages, calculated if more than 4 observations with the same light or temperature value existed. Measurements from only those days when leaves were degrading chlorophyll were considered. (c) Effects of the redness index on the rate of chlorophyll degradation (µg cm^-2^ chlorophyll (*a* + *b*) day^-1^). Only leaves with chlorophyll contents of 10‒20 µg cm^-2^ were considered. See Supplementary Table S3 for the average chlorophyll values. The measurements are from 27 leaves, belonging to four trees. Pigment data are from Supplementary Figs. S4‒7. Weather data is from Supplementary Fig. S8.


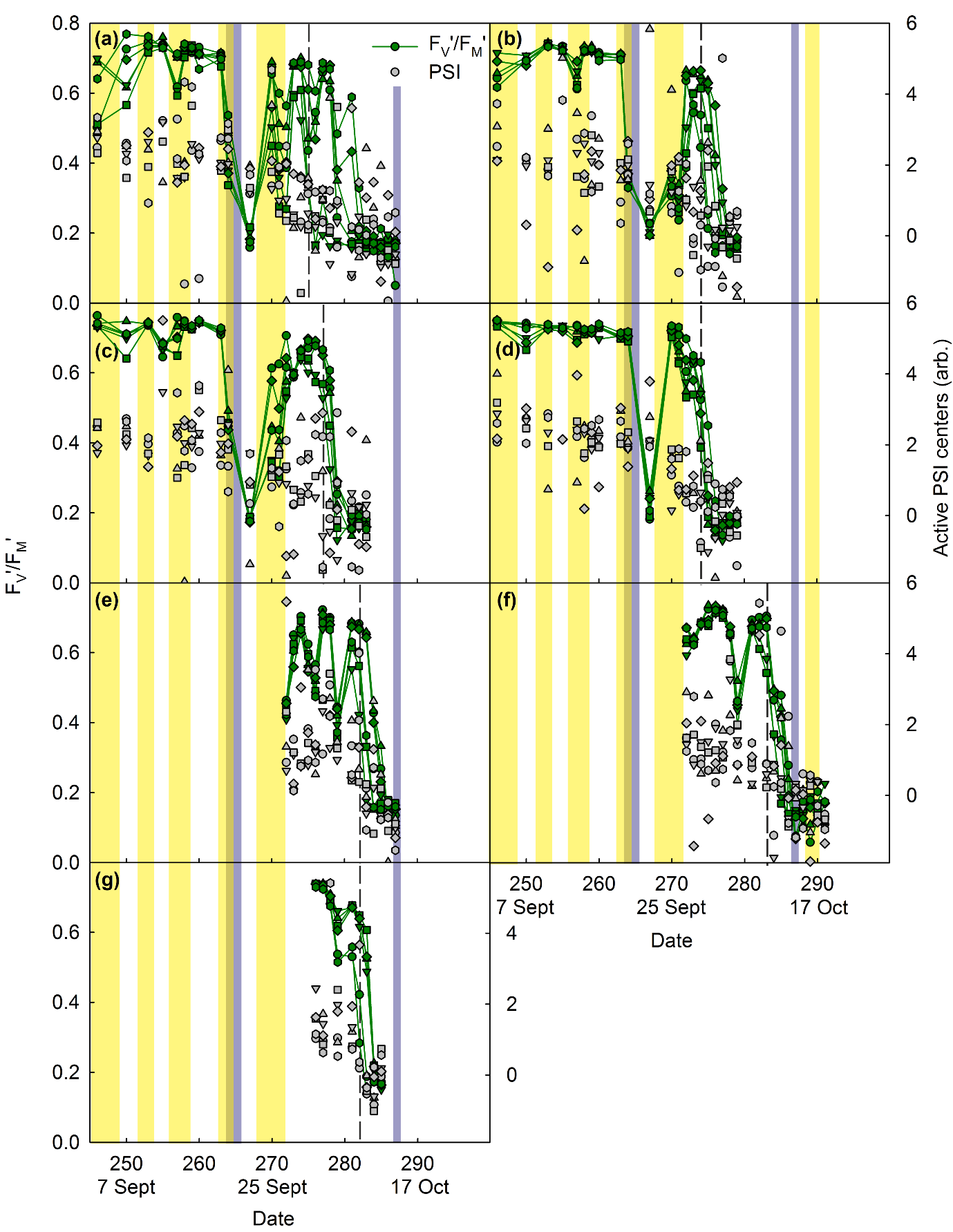


Supplementary Fig. S10. The fluorescence parameter (F_M_’-F_0_’)/F_M_’ = F_V_’/F_M_’ (green symbols, solid line), reflecting functional PSII units, and oxidizable P700, during a saturating pulse, reflecting active PSI centres (grey symbols; arbitrary units), in seven leaves (a‒g) of a maple tree during autumn 2021, until abscission. Six measurement sites per leaf are marked with different symbols (see Supplementary Fig. S3 for the positions on the leaf blade). The vertical dashed line highlights the time point at which all chlorophyll was degraded in one of the measurement sites of the leaf. The vertical dashed line highlights the time point at which all chlorophyll was degraded in one of the measurement sites of the leaf. The vertical yellow bars indicate days with high irradiance (daily irradiance > 3000 Wm^-2^, except on 16‒17 Oct ~2000 Wm^-2^) and the blue bars indicate days when the previous night had been cold (temperature below 0 °C). Weather data were measured by Finnish meteorological institute.


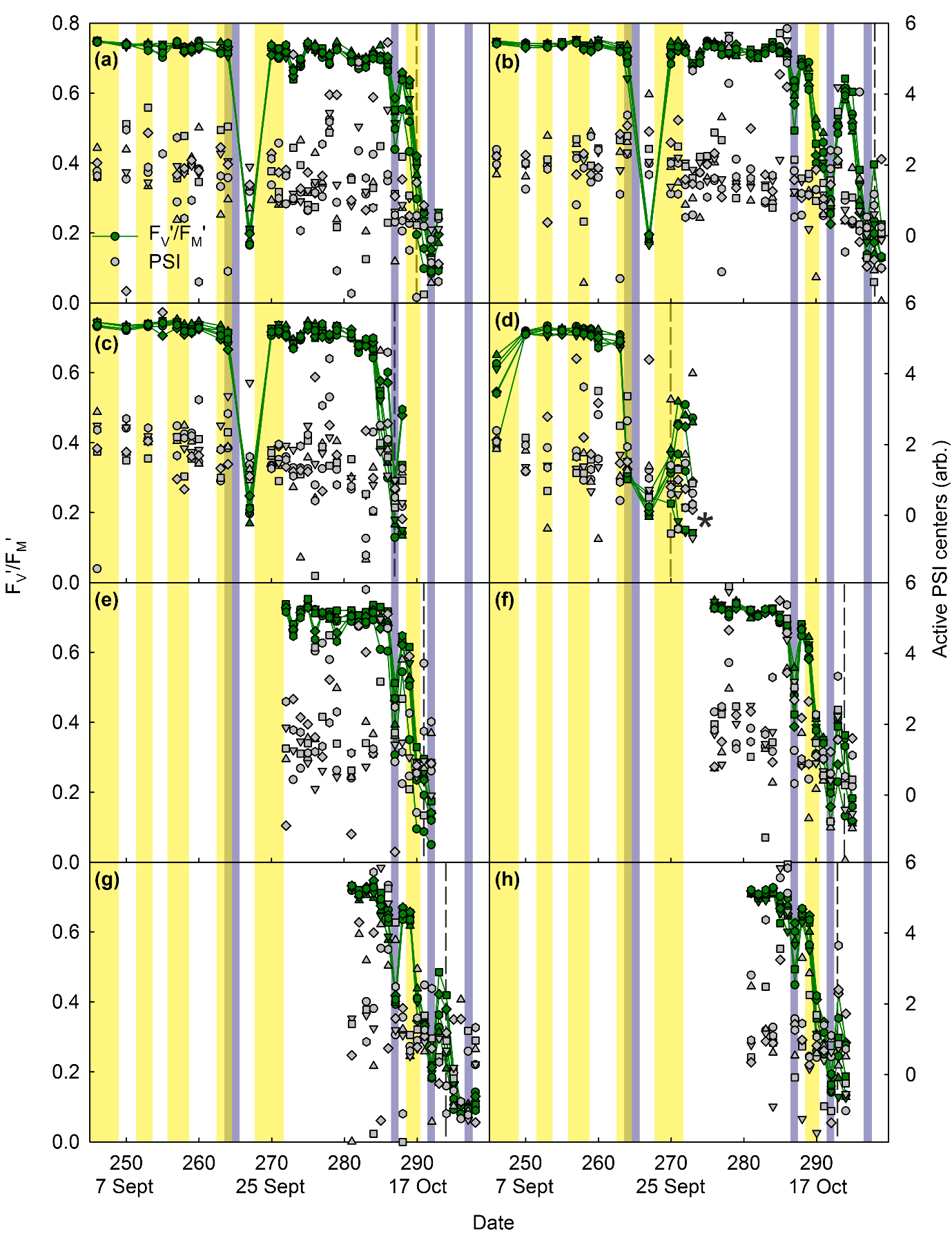


Supplementary Fig. S11. The fluorescence parameter (F_M_’-F_0_’)/F_M_’ = F_V_’/F_M_’ (green symbols, solid line), reflecting functional PSII units, and oxidizable P700, during a saturating pulse, reflecting active PSI centres (grey symbols; arbitrary units), in eight leaves (a‒h) of a maple tree during autumn 2021, until abscission. Six measurement sites per leaf are marked with different symbols (see Supplementary Fig. S4 for the positions on the leaf blade). The vertical dashed line highlights the time point at which all chlorophyll was degraded in one of the measurement sites of the leaf. The vertical dashed line highlights the time point at which all chlorophyll was degraded in one of the measurement sites of the leaf. The vertical yellow bars indicate days with high irradiance (daily irradiance > 3000 Wm^-2^, except on 16‒17 Oct ~2000 Wm^-2^) and the blue bars indicate days when the previous night had been cold (temperature below 0 °C). Weather data were measured by Finnish meteorological institute.


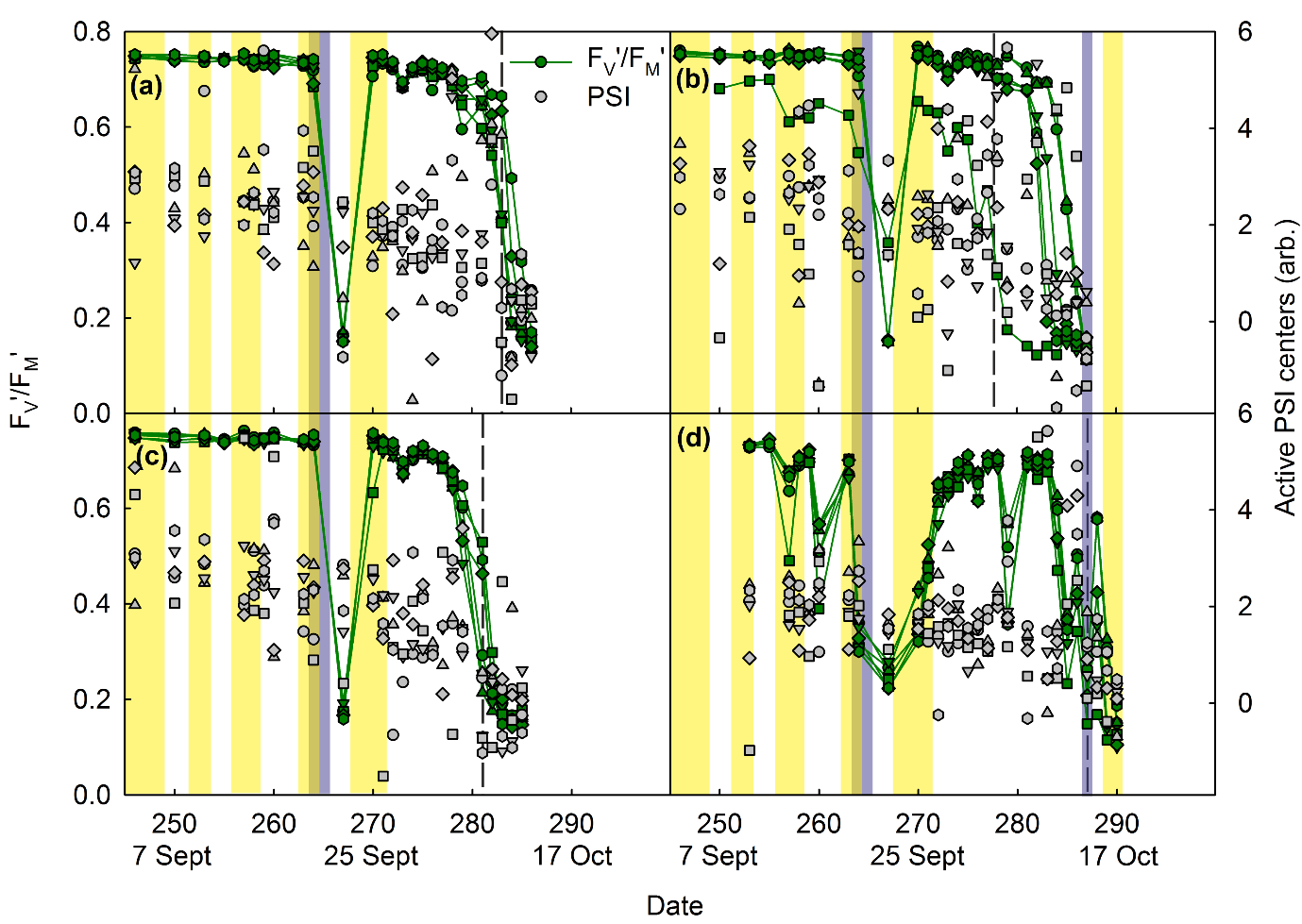


Supplementary Fig. S12. The fluorescence parameter (F_M_’-F_0_’)/F_M_’ = F_V_’/F_M_’ (green symbols, solid line), reflecting functional PSII units, and oxidizable P700, during a saturating pulse, reflecting active PSI centres (grey symbols; arbitrary units), in four leaves (a‒d) of a maple tree during autumn 2021, until abscission. Six measurement sites per leaf are marked with different symbols (see Supplementary Fig. S4 for the positions on the leaf blade). The vertical dashed line highlights the time point at which all chlorophyll was degraded in one of the measurement sites of the leaf. The vertical dashed line highlights the time point at which all chlorophyll was degraded in one of the measurement sites of the leaf. The vertical yellow bars indicate days with high irradiance (daily irradiance > 3000 Wm^-2^, except on 16‒17 Oct ~2000 Wm^-2^) and the blue bars indicate days when the previous night had been cold (temperature below 0 °C). Weather data were measured by Finnish meteorological institute.


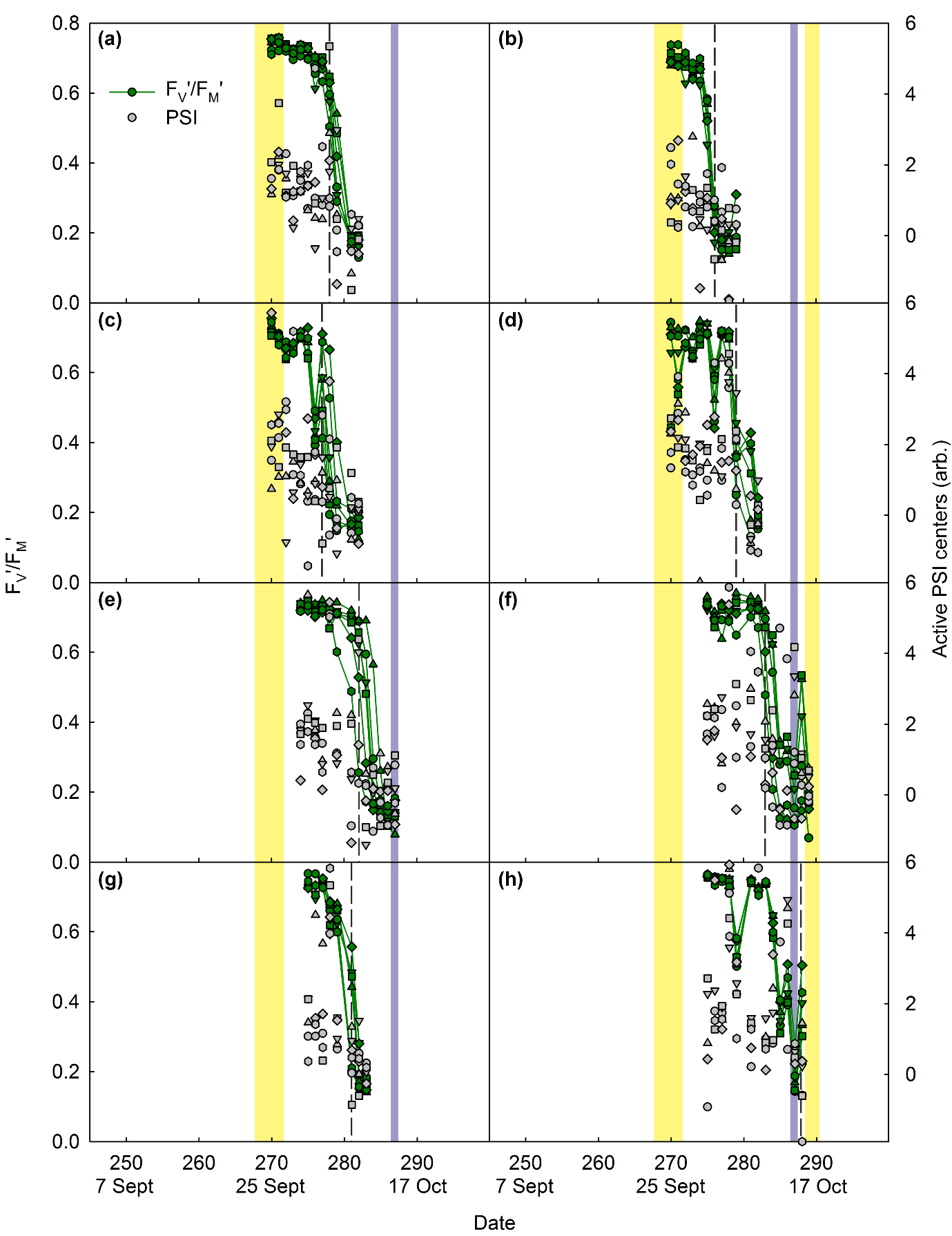


Supplementary Fig. S13. The fluorescence parameter (F_M_’-F_0_’)/F_M_’ = F_V_’/F_M_’ (green symbols, solid line), reflecting functional PSII units, and oxidizable P700, during a saturating pulse, reflecting active PSI centres (grey symbols; arbitrary units), in eight leaves (a‒h) of a maple tree during autumn 2021, until abscission. Six measurement sites per leaf are marked with different symbols (see Supplementary Fig. S4 for the positions on the leaf blade). The vertical dashed line highlights the time point at which all chlorophyll was degraded in one of the measurement sites of the leaf. The vertical dashed line highlights the time point at which all chlorophyll was degraded in one of the measurement sites of the leaf. The vertical yellow bars indicate days with high irradiance (daily irradiance >3000 Wm^-2^, except on 16‒17 Oct ~2000 Wm^-2^) and the blue bars indicate days when the previous night had been cold (temperature below 0 °C). Weather data were measured by Finnish meteorological institute.


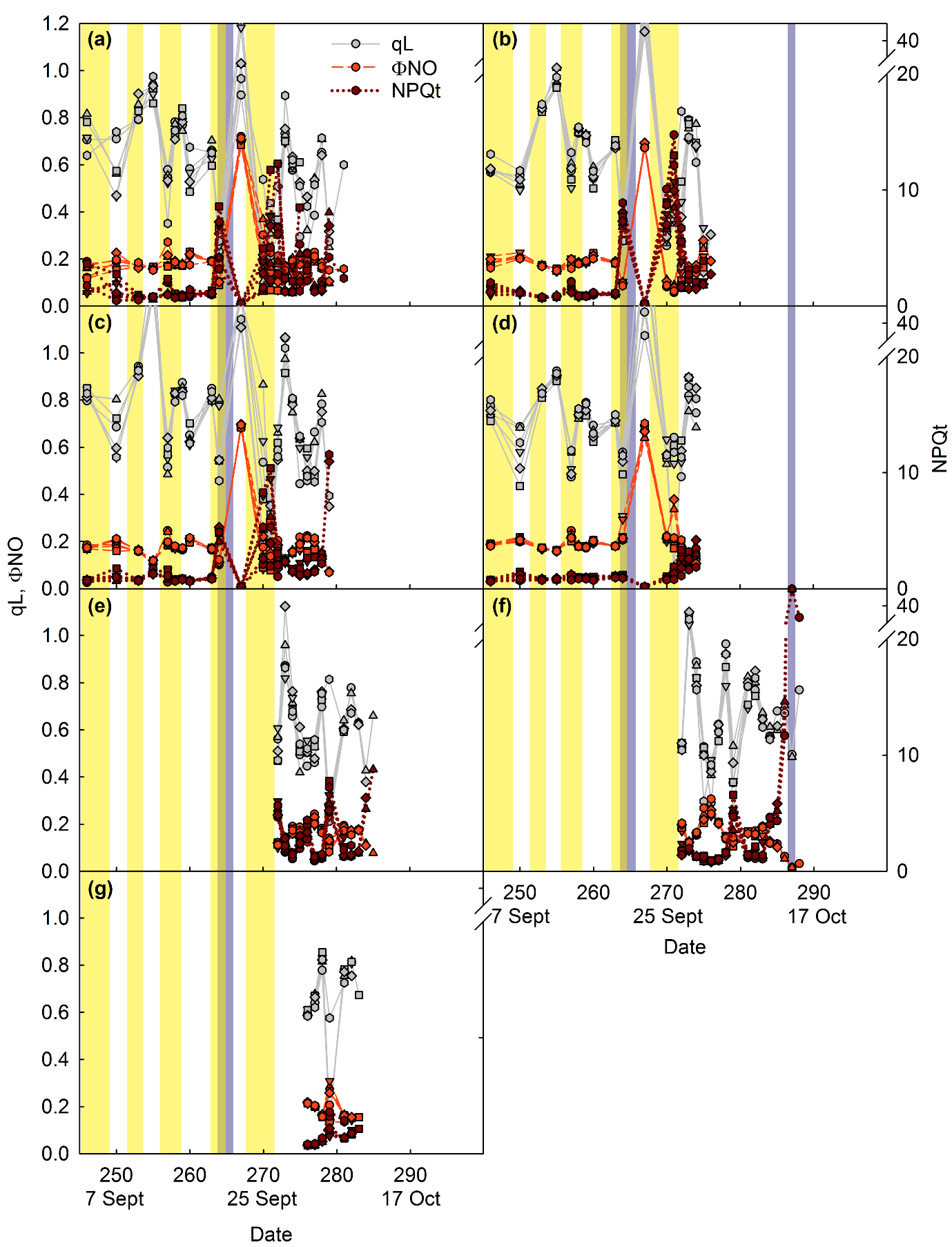


Supplementary Fig. S14. Photochemical quenching (qL; grey symbols, solid line), the yield of unregulated non-photochemical quenching (ΦNO; red symbols, dashed line) and regulated non-photochemical quenching (NPQt; dark red symbols, dotted line) of chlorophyll fluorescence in seven leaves (a‒g) of a maple tree during autumn 2021. qL was calculated as (F_M_’-F_S_)/(F_M_’-F_0_′)*(F_0_′/F_S_), NPQt as (4.88/((F_M_′/F_0_′)-1))-1 and ΦNO as 1/(NPQt+1+qL*4.88). Six measurements per leaf were conducted, marked with different symbols (see Supplementary Fig. S4 for the positions on the leaf blade). The vertical yellow bars indicate days with high irradiance (daily irradiance > 3000 Wm^-2^, except on 16‒17 Oct ~2000 Wm^-2^) and the blue bars indicate days when the previous night had been cold (temperature below 0 °C). Weather data were measured by Finnish meteorological institute. Measurements conducted on leaf areas with no chlorophyll (chlorophyll content less than 0.5 µg cm^-2^) have been excluded.


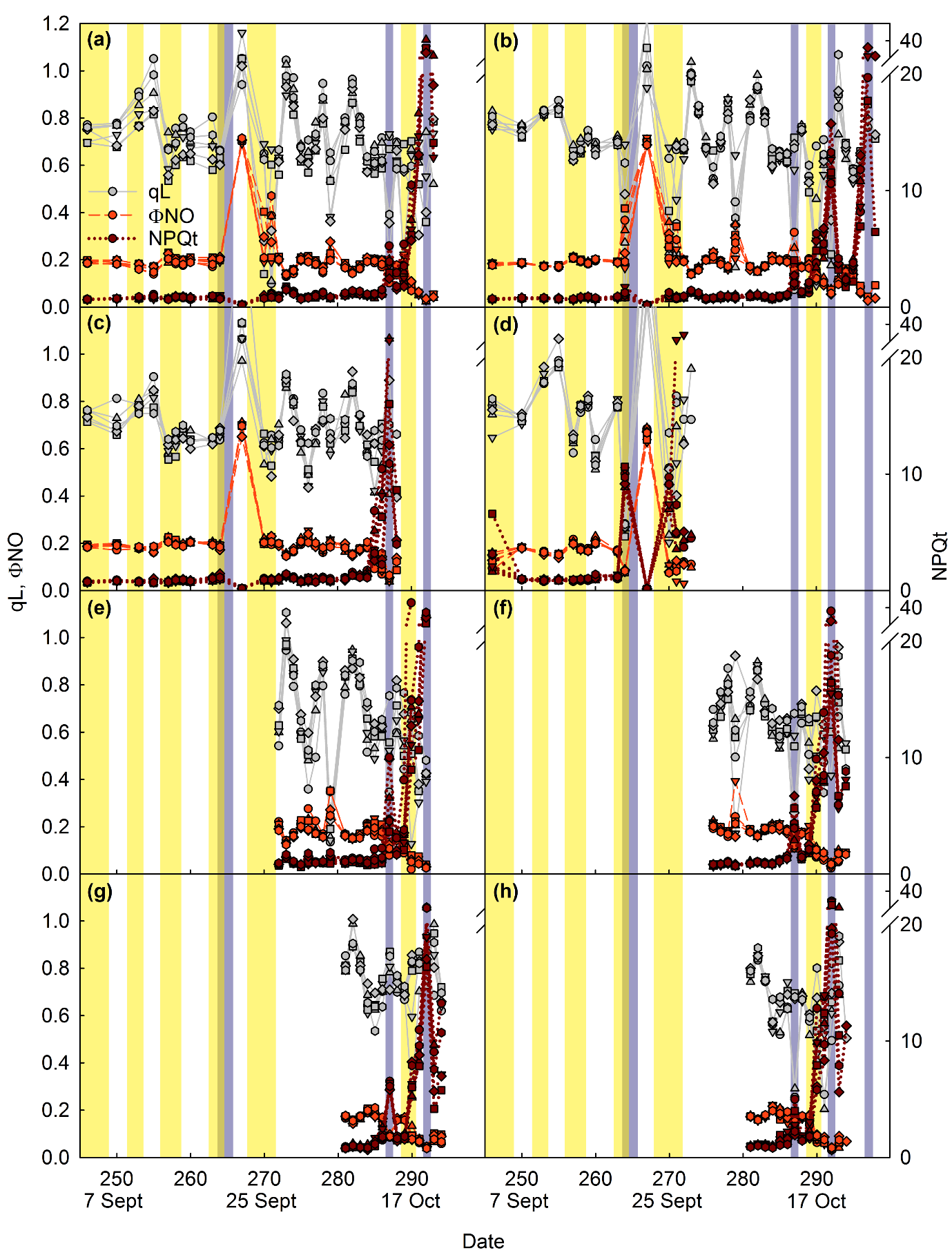


Supplementary Fig. S15. Photochemical quenching (qL; grey symbols, solid line), the yield of unregulated non-photochemical quenching (ΦNO; red symbols, dashed line) and regulated non-photochemical quenching (NPQt; dark red symbols, dotted line) of chlorophyll fluorescence in eight leaves (a‒h) of a maple tree during autumn 2021. qL was calculated as (F_M_’-F_S_)/(F_M_’-F_0_′)*(F_0_′/F_S_), NPQt as (4.88/((F_M_′/F_0_′)-1))-1 and ΦNO as 1/(NPQt+1+qL*4.88). Six measurements per leaf were conducted, marked with different symbols (see Supplementary Fig. S4 for the positions on the leaf blade). The vertical yellow bars indicate days with high irradiance (daily irradiance > 3000 Wm^-2^, except on 16‒17 Oct ~2000 Wm^-2^) and the blue bars indicate days when the previous night had been cold (temperature below 0 °C). Weather data were measured by Finnish meteorological institute. Measurements conducted on leaf areas with no chlorophyll (chlorophyll content less than 0.5 µg cm^-2^) have been excluded.


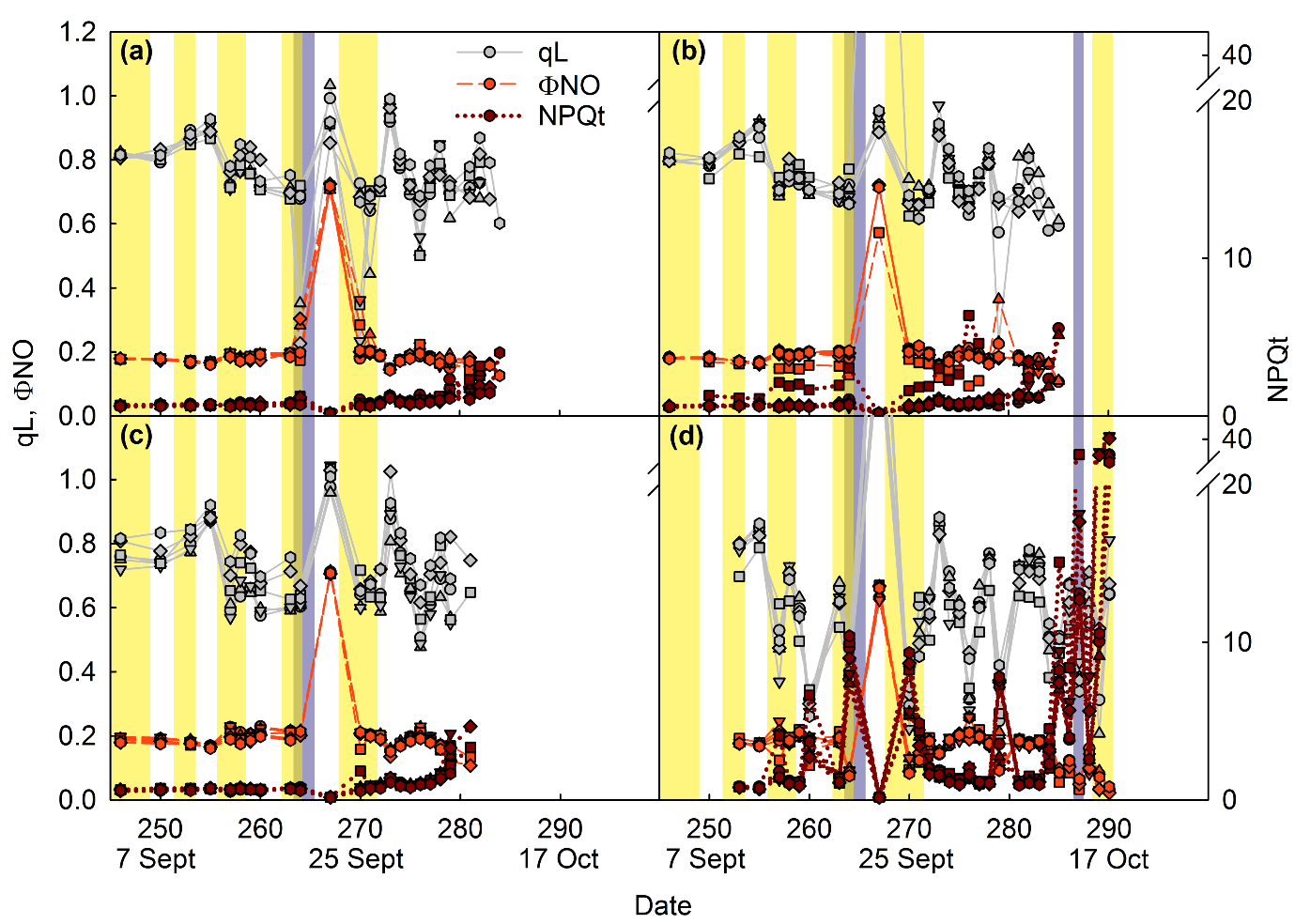


Supplementary Fig. S16. Photochemical quenching (qL; grey symbols, solid line), the yield of unregulated non-photochemical quenching (ΦNO; red symbols, dashed line) and regulated non-photochemical quenching (NPQt; dark red symbols, dotted line) of chlorophyll fluorescence in four leaves (a‒d) of a maple tree during autumn 2021. qL was calculated as (F_M_’-F_S_)/(F_M_’-F_0_′)*(F_0_′/F_S_), NPQt as (4.88/((F_M_′/F_0_′)-1))-1 and ΦNO as 1/(NPQt+1+qL*4.88). Six measurements per leaf were conducted, marked with different symbols (see Supplementary Fig. S4 for the positions on the leaf blade). The vertical yellow bars indicate days with high irradiance (daily irradiance > 3000 Wm^-2^, except on 16‒17 Oct ~2000 Wm^-2^) and the blue bars indicate days when the previous night had been cold (temperature below 0 °C). Weather data were measured by Finnish meteorological institute. Measurements conducted on leaf areas with no chlorophyll (chlorophyll content less than 0.5 µg cm^‑2^) have been excluded.


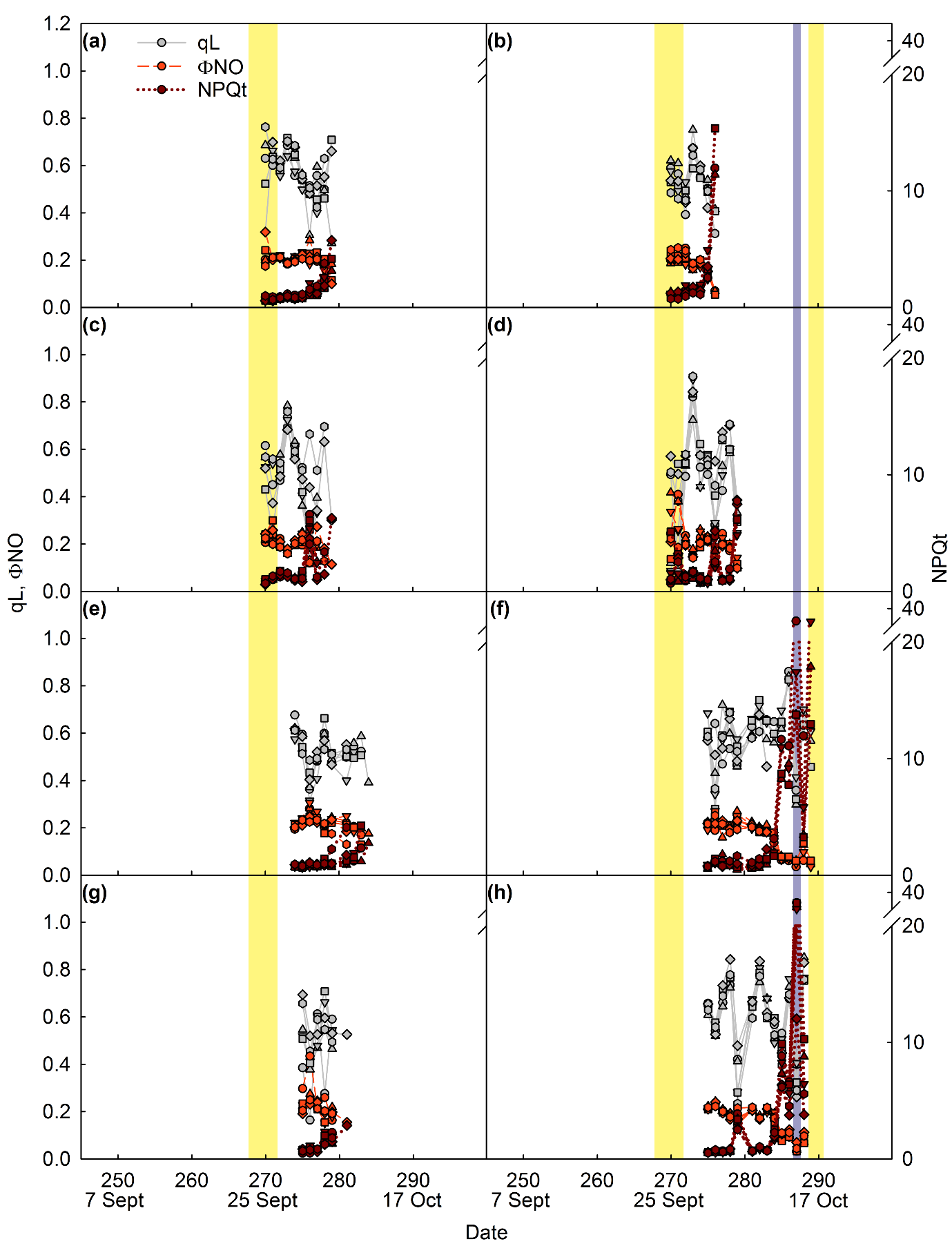


Supplementary Fig. S17. Photochemical quenching (qL; grey symbols, solid line), the yield of unregulated non-photochemical quenching (ΦNO; red symbols, dashed line) and regulated non-photochemical quenching (NPQt; dark red symbols, dotted line) of chlorophyll fluorescence in eight leaves (a‒h) of a maple tree during autumn 2021. qL was calculated as (F_M_’-F_S_)/(F_M_’-F_0_′)*(F_0_′/F_S_), NPQt as (4.88/((F_M_′/F_0_′)-1))-1 and ΦNO as 1/(NPQt+1+qL*4.88). Six measurements per leaf were conducted, marked with different symbols (see Supplementary Fig. S4 for the positions on the leaf blade). The vertical yellow bars indicate days with high irradiance (daily irradiance > 3000 Wm^-2^, except on 16‒17 Oct ~2000 Wm^-2^) and the blue bars indicate days when the previous night had been cold (temperature below 0 °C). Weather data were measured by Finnish meteorological institute. Measurements conducted on leaf areas with no chlorophyll (chlorophyll content less than 0.5 µg cm^-2^) have been excluded.


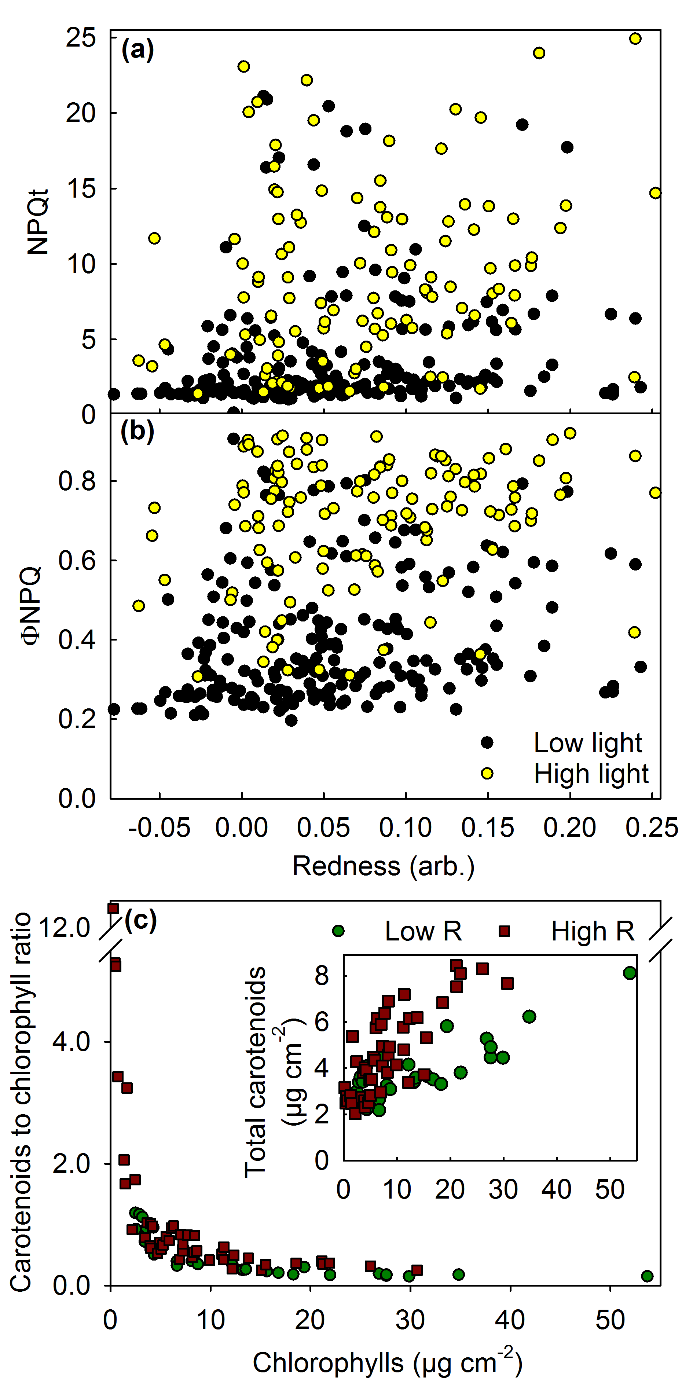


Supplementary Fig. S18. NPQ and carotenoids in maple leaves with different amounts of red pigments. Redness index (R) was calculated based on light absorbance at 530 nm. NPQt (a) and NPQ yield (ΦNPQ; b) were measured *in vivo* from senescing maple leaves in their natural environment. Measurements were taken from leaves with chlorophyll (*a* + *b*) content of 5–20 µg cm^-2^. Solid symbols show measurements taken during conditions with low ambient light (PPFD < 100 µmol m^-2^ s^-1^) and yellow symbols during conditions with higher ambient light (PPFD > 100 µmol m^-2^ s^-1^). The data is from Figs. 3 and 5 and Supplementary Figs. S4–7 and S14–17. See Supplementary Table S3 for the average chlorophyll values. (c) Carotenoid to chlorophyll ratio and the total amount of carotenoids (the inset) in maple leaf pieces with different contents of chlorophyll and red pigments. After the measurement of 530 nm absorbance, pigments were extracted in dimethylformamide and quantified spectroscopically. Each symbol represents an individual measurement.

**Supplementary tables**

Supplementary Table S1. Number of leaf sections (sites), leaves and individual trees and the N and statistical tests used in the analyses shown in the figures.

| **Figure** | **Groups** | **Sites /leaf** | **Leaves** | **Trees** | **N** | **Statistical tests** | **Notes** |
| --- | --- | --- | --- | --- | --- | --- | --- |
| **Fig. 1** | **Green** | 3 | 4 | 4 | 4 | Average, SD |  |
|  | **Yellow** | 2–3 | 4 | 4 | 4 |  |  |
|  | **Red** | 3–5 | 4 | 4 | 4 |  |  |
| **Fig. 2a** | **see Fig. S2** |  |  |  |  | Linear model for k_PI_ | Pairs of yellow and red parts always from the same leaf |
| **Fig. 2b** | **see Fig. S2** |  |  |  |  | Linear model for recovery from photo-inhibition |  |
| **Figs. 3–6, 7c,d, S3–S7, S10–S17** | **Tree 1 (a)** | 6 | 7 | 1 | 3174 | - | 40 time points. Values have been excluded in some Figures |
|  | **Tree 2 (b)** | 6 | 8 | 1 |  |  |  |
|  | **Tree 3 (c)** | 6 | 4 | 1 |  |  |  |
|  | **Tree 4 (d)** | 6 | 8 | 1 |  |  |  |
| **Fig. 7a** |  | 1–12 | 40 | 20 | 256 | - |  |
| **Fig. 7b** | **Low R** | 1–12 | 20 | 20 | 47 | Average, SD | Includes all leaf pieces of Fig. 2 |
|  | **Medium R** |  |  |  | 21 |  |  |
|  | **High R** |  |  |  | 40 |  |  |
|  | **Very high R** |  |  |  | 32 |  |  |
| **Fig. 7e (Green)** | **Very low R** | 1–6 | 27 | 4 | 366 | Average, SD | 40 time points |
|  | **Low R** |  |  |  | 189 |  |  |
|  | **Medium R** |  |  |  | 292 |  |  |
|  | **High R** |  |  |  | 53 |  |  |
| **Fig. 7e (Senes-cing)** | **Very low R** |  |  |  | 106 |  |  |
|  | **Low R** |  |  |  | 46 |  |  |
|  | **Medium R** |  |  |  | 106 |  |  |
|  | **High R** |  |  |  | 78 |  |  |
|  | **Very high R** |  |  |  | 11 |  |  |
| **Fig. 7f (Green)** | **Very low F_V_’/F_M_’** | 1–6 | 27 | 4 | 50 | Average, SD | 40 time points |
|  | **Low F_V_’/F_M_’** |  |  |  | 15 |  |  |
|  | **Medium F_V_’/F_M_’** |  |  |  | 18 |  |  |
|  | **High F_V_’/F_M_’** |  |  |  | 24 |  |  |
|  | **Very high F_V_’/F_M_’** |  |  |  | 792 |  |  |
| **Fig. 7f (Senes-cing)** | **Very low F_V_’/F_M_’** |  |  |  | 123 |  |  |
|  | **Low F_V_’/F_M_’** |  |  |  | 61 |  |  |
|  | **Medium F_V_’/F_M_’** |  |  |  | 69 |  |  |
|  | **High F_V_’/F_M_’** |  |  |  | 93 |  |  |
|  | **Very high F_V_’/F_M_’** |  |  |  | 268 |  |  |
| **Fig. 8 (0.5 to 10)** | **Low R** | 1–6 | 27 | 4 | 182 | Average, SD | 40 time points |
|  | **Medium R** |  |  |  | 89 |  |  |
|  | **High R** |  |  |  | 179 |  |  |
| **Fig. 8 (10 to 25)** | **Low R** |  |  |  | 946 |  |  |
|  | **Medium R** |  |  |  | 157 |  |  |
|  | **High R** |  |  |  | 51 |  |  |
| **Fig. 8 (over 25)** | **Low R** |  |  |  | 638 |  |  |
|  | **Medium R** |  |  |  | 371 |  |  |
|  | **High R** |  |  |  | 68 |  |  |
| **Fig. 9** | **Early** | 1 | 9 | 3 | 9 | Average, SD, t-test (hetero-scedastic) | Aspen, early and late from the same trees |
|  | **Late** | 1 | 9 | 3 | 9 |  |  |
|  | **Fallen** | 1 | 10 | 4(?) | 10 |  |  |
| **Fig. S1** | **a** | 1–2 | 11 | 3 | 16 | Linear regression |  |
|  | **b, green and red** | 1 | 7 | 3 | 23 | - |  |
|  | **b, brown** | 5–6 | 3 | 3 | 3 | Average, SD |  |
| **Fig. S2a** | **Green leaves** | 1 | 16 | 16 | 16 | Average, SD | Pairs of yellow and red parts always from the same leaf (in each treatment), green parts were also cut from those leaves (but not always included) |
|  | **Green parts** | 1–3 | 10 | 10 | 10 |  |  |
|  | **Yellow and red parts** |  | 8 | 8 | 8 |  |  |
| **Fig. S2b** | **Green leaves** | 1 | 16 | 16 | 16 |  |  |
|  | **Green parts** | 1–3 | 6 | 6 | 6 |  |  |
|  | **Yellow and red parts** |  | 10 | 10 | 10 |  |  |
| **Fig. S2c** | **Green leaves** | 1 | 12 | 12 | 12 |  |  |
|  | **Green parts** | 1–3 | 6 | 6 | 6 |  |  |
|  | **Yellow and red parts** |  | 7 | 7 | 7 |  |  |
| **Fig. S2d** | **Green leaves** | 1 | 12 | 12 | 12 |  |  |
|  | **Green parts** | 1–3 | 5 | 5 | 5 |  |  |
|  | **Yellow and red parts** |  | 9 | 9 | 9 |  |  |
| **Fig. S2e** | **Green leaves** | 1 | 12 | 12 | 12 |  |  |
|  | **Green parts** | 1–3 | 7 | 7 | 7 |  |  |
|  | **Yellow and red parts** |  | 6 | 6 | 6 |  |  |
| **Fig. S2f** | **Green leaves** | 1 | 12 | 12 | 12 |  |  |
|  | **Green parts** | 1–3 | 5 | 5 | 5 |  |  |
|  | **Yellow and red parts** |  | 6 | 6 | 6 |  |  |
| **Fig. S2g** | **Green leaves** | 1 | 16 | 16 | 16 |  |  |
|  | **Green parts** | 1–3 | 7 | 7 | 7 |  |  |
|  | **Yellow and red parts** |  | 9 | 9 | 9 |  |  |
| **Fig S9** | **a,b** | 1–6 | 27 | 4 | 1289 | Average | 40 time points |
|  | **c** |  |  |  | 389 | - |  |
| **Fig. S18a,b** | **Low light** | 1–6 | 27 | 4 | 195 | - | 40 time points |
|  | **High light** |  |  |  | 122 |  |  |
| **Fig. S18c** | **Low R** | 1–3 | 10 | 5 | 28 | - |  |
|  | **High R** |  |  |  | 47 |  |  |

Supplementary Table S2. Quantification of photoinhibition and recovery in green and senescing maple leaves (Fig. 2). Remaining PSII activity (%) after illumination or dark-incubation (after HL) and over-night recovery (Recovery) as well as the recovery amount (Recovery - After HL) were calculated based on the F_V_/F_M_ values shown in Fig. S2. Each number represents an average of at least four biological replicates ± SD.

|  |  | **No lincomycin** | | | **Lincomycin** | | |  |
| --- | --- | --- | --- | --- | --- | --- | --- | --- |
|  |  | **After HL (%)** | **Recovery (%)** | **Recovery amount** | **After HL (%)** | **Recovery (%)** | **Recovery amount** | |
| **White light** | **Green leaves** | 64 ± 8 | 100 ± 6 | 0.27 ± 0.05 | 51 ± 13 | 73 ± 9 | 0.17 ± 0.07 | |
|  | **Green parts** | 54 ± 12 | 91 ± 13 | 0.26 ± 0.04 | 35 ± 10 | 65 ± 19 | 0.21 ± 0.08 | |
|  | **Yellow parts** | 32 ± 15 | 65 ± 25 | 0.23 ± 0.18 | 29 ± 13 | 60 ± 18 | 0.22 ± 0.07 | |
|  | **Red parts** | 35 ± 20 | 77 ± 21 | 0.25 ± 0.14 | 31 ± 16 | 62 ± 18 | 0.19 ± 0.06 | |
| **Green light** | **Green leaves** | 88 ± 4 | 102 ± 4 | 0.10 ± 0.04 | 87 ± 5 | 95 ± 2 | 0.06 ± 0.03 | |
|  | **Green parts** | 90 ± 4 | 101 ± 4 | 0.08 ± 0.04 | 82 ± 5 | 95 ± 5 | 0.09 ± 0.01 | |
|  | **Yellow parts** | 85 ± 8 | 92 ± 3 | 0.05 ± 0.06 | 84 ± 5 | 89 ± 4 | 0.04 ± 0.03 | |
|  | **Red parts** | 83 ± 23 | 76 ± 54 | -0.05 ± 0.24 | 85 ± 9 | 87 ± 7 | 0.02 ± 0.02 | |
| **Red light** | **Green leaves** | 74 ± 10 | 99 ± 4 | 0.19 ± 0.06 | 78 ± 6 | 91 ± 4 | 0.10 ± 0.05 | |
|  | **Green parts** | 59 ± 9 | 84 ± 25 | 0.18 ± 0.16 | 52 ± 10 | 79 ± 13 | 0.19 ± 0.07 | |
|  | **Yellow parts** | 52 ± 19 | 87 ± 7 | 0.26 ± 0.11 | 39 ± 21 | 71 ± 15 | 0.22 ± 0.06 | |
|  | **Red parts** | 35 ± 19 | 66 ± 35 | 0.18 ± 0.13 | 26 ± 24 | 56 ± 23 | 0.18 ± 0.08 | |
| **Dark** | **Green leaves** | 99 ± 1 | 101 ± 8 | n.d. | n.d. | | | |
|  | **Green parts** | 98 ± 1 | 94 ± 20 | n.d. |  |  |  |  |
|  | **Yellow parts** | n.d. | 91 ± 16 | n.d. |  |  |  |  |
|  | **Red parts** | n.d. | 105 ± 14 | n.d. |  |  |  |  |

Supplementary Table S3. Average chlorophyll contents and redness indexes (estimated based on 530 nm absorbance, arbitrary units), at the beginning of the experiments, of leaf pieces used in photoinhibition experiments (Fig. 2). Leaf chlorophyll contents were measured with an optical method, and then converted to µg cm^-2^ using a calibration curve. Numbers represent averages of at least four biological replicates ± SD. n.d. = not determined.

|  |  | **No lincomycin** | | **Lincomycin** | |
| --- | --- | --- | --- | --- | --- |
|  |  | **Chlorophyll (µg cm^-2^)** | **530 nm absorbance (arb.)** | **Chlorophyll (µg cm^-2^)** | **530 nm absorbance (arb.)** |
| **White light** | **Green leaves** | 30.6 ± 8.1 | 0.018 ± 0.041 | 29.1 ± 12.4 | 0.009 ± 0.042 |
|  | **Green parts** | 20.6 ± 6.4 | 0.039 ± 0.029 | 19.8 ± 5.6 | 0.047 ± 0.045 |
|  | **Yellow parts** | 5.1 ± 3.6 | 0.040 ± 0.026 | 7.4 ± 3.6 | 0.051 ± 0.032 |
|  | **Red parts** | 5.0 ± 3.6 | 0.219 ± 0.106 | 8.2 ± 4.3 | 0.210 ± 0.061 |
| **Green light** | **Green leaves** | 35.5 ± 7.6 | 0.031 ± 0.039 | 30.5 ± 12.4 | 0.016 ± 0.031 |
|  | **Green parts** | 24.5 ± 7.0 | 0.042 ± 0.028 | 18.1 ± 4.8 | 0.047 ± 0.031 |
|  | **Yellow parts** | 7.5 ± 3.6 | 0.038 ± 0.031 | 7.4 ± 3.6 | 0.059 ± 0.033 |
|  | **Red parts** | 4.2 ± 1.8 | 0.219 ± 0.069 | 6.2 ± 2.6 | 0.246 ± 0.056 |
| **Red light** | **Green leaves** | 32.8 ± 8.7 | 0.016 ± 0.043 | 32.2 ± 7.9 | 0.001 ± 0.028 |
|  | **Green parts** | 18.6 ± 7.9 | 0.052 ± 0.022 | 14.7 ± 1.4 | 0.030 ± 0.032 |
|  | **Yellow parts** | 12.0 ± 11.9 | 0.010 ± 0.035 | 7.4 ± 4.6 | 0.064 ± 0.037 |
|  | **Red parts** | 6.2 ± 3.4 | 0.215 ± 0.085 | 5.6 ± 3.7 | 0.226 ± 0.078 |
| **Dark** | **Green leaves** | 30.5 ± 8.4 | 0.026 ± 0.041 | n.d. | |
|  | **Green parts** | 21.6 ± 5.0 | 0.042 ± 0.009 |  |  |
|  | **Yellow parts** | 6.3 ± 3.1 | 0.048 ± 0.069 |  |  |
|  | **Red parts** | 5.0 ± 2.2 | 0.150 ± 0.053 |  |  |

Supplementary Table S4. Average chlorophyll contents ± SD of leaves or leaf pieces, with very low, low, medium, high or very high redness indexes (estimated based on 530 nm absorbance), or with very low, low, medium, high or very high F_V_’/F_M_’ values, of the indicated figures. Green indicates non-senescing leaves or leaf pieces, as specified in the respective figure legend.

|  |  | **Chlorophyll (*a* + *b*), µg cm^-2^ (SD)** | | | | |
| --- | --- | --- | --- | --- | --- | --- |
| **Figure** | **Leaf type** | **Redness index** | | | | |
|  |  | **Very low**  **<0.025** | **Low**  **0.025–0.05** | **Medium**  **0.05–0.1** | **High**  **0.1–0.2** | **Very high**  **>0.2** |
| **Fig. 1** | **Green** | - | | 31.6 ± 9.1 | - | - |
|  | **Yellow** | - | | - | 8.1 ± 5.0 | - |
|  | **Red** | - | | - | - | 2.3 ± 1.6 |
| **Fig. 7b** | **Senescing** | 4.99 ± 2.44 | | 5.21 ± 3.08 | 4.18 ± 2.66 | 4.12 ± 2.24 |
| **Fig. 7e** | **Senescing** | 7.24 ± 2.30 | 7.25 ± 2.36 | 7.18 ± 2.45 | 6.36 ± 0.42 | 6.35 ± 2.44 |
|  | **Green** | 35.0 ± 4.23 | 35.0 ± 3.72 | 38.4 ± 5.07 | 41.8 ± 7.15 | - |
| **Fig. 8b** | **Senescing** | 5.71 ± 2.91 | | 4.88 ± 2.91 | 3.52 ± 2.74 | |
|  | **Senescing** | 19.5 ± 3.92 | | 17.6 ± 4.50 | 17.1± 4.56 | |
|  | **Green** | 31.9 ± 4.89 | | 36.1 ± 6.30 | 38.5 ± 8.89 | |
| **Fig. S9c** | **Senescing** | 15.9 ± 2.72 | | 14.5 ± 2.76 | 14.0 ± 2.99 | |
| **Fig. S18a,b** | **Senescing** | 7.24 ± 2.31 | | 7.18 ± 2.45 | 6.36 ± 2.36 | |
|  |  | **F_V_’/F_M_’ values** | | | | |
|  |  | **Very low**  **<0.4** | **Low**  **0.4–0.5** | **Medium**  **0.5–0.6** | **High**  **0.6–0.65** | **Very high**  **>0.65** |
| **Fig. 7f** | **Senescing** | 11.4 ± 4.43 | 12.6 ± 4.47 | 12.9 ± 4.31 | 11.2 ± 4.33 | 14.8 ± 3.88 |
|  | **Green** | 34.7 ± 4.14 | 33.1 ± 2.36 | 35.3 ± 2.87 | 35.4 ± 3.53 | 36.8 ± 5.19 |

**Summary of statistics**

**1. Boxplots**

Fig. S1.1. The PPFD of the measurement, μmol m^-2^s^-1^, as a function of Julian day.

Fig. S1.2. Leaf temperature, °C, as a function of Julian day.

Fig. S1.3. Leaf chlorophyll content, μg cm^-2^, as a function of Julian day.

Fig. S1.4. Redness index as a function of Julian day

Fig. S1.5. F_V_'/F_M_' as a function of Julian day

Fig. S1.6. The gH+ parameter as a function of Julian day.

Fig. S1.7. Non-photochemical quenching (NPQt) as a function of Julian day.

Fig. S1.8. Relative number of active PSI centres as a function of Julian day.

Fig. S1.9. Leaf thickness, in mm, as a function of Julian day.

**2. A Linear Mixed Effect Model for gH^+^**

**Justification of model variables**

PPFD may regulate gH^+^. Chlorophyll content and the number of days after 2 Sep 2021 describe the advancement of autumn senescence, and redness is the main topic of the study. Random effects specific to the sample, Julian day or the tree individual were considered possible.

**Call of the R function lmer**

lmer(yeo.johnson(gH, 0.6) ~ PPFDdiv100 + LogCHL + REDNESS + LogDays + (1 | ID) + (1 | LogDays) + (1 | Tree)

**Variable conversions and data filtering applied in the model of gH+**

The response variable (gH+), was Yeo-Johnson transformed, λ = 0.6; PPFD, expressed in µmol m^-2^s^-1^, was divided by 100 (PPFDdiv100); Chlorophyll concentration, expressed as µg cm^-2^, was log transformed (LogCHL); The redness index was used as such (REDNESS); The number of days after 2 Sep 2 2021 was log transformed (LogDays) and used as both a fixed and a random effect.

The data were filtered by removing all rows with chlorophyll content lower or equal than 0.5 µg cm^-2^.

gH+ values outside of the range 2 <= gH+ < 160 were removed from the data, and also lines with PPFD values below 1 µmol m^-2^s^-1^ were removed.

**Summary of the model for gH+**

Linear mixed model fit by REML ['lmerMod']

REML criterion at convergence: 570.8

Table S2.1. Scaled residuals of the model for gH+

| Min | 1Q | Median | 3Q | Max |
| --- | --- | --- | --- | --- |
| -3.3641 | -0.5716 | -0.0408 | 0.5832 | 3.6924 |

Table S2.2. Random effects of the model for gH+

| Groups | Name | Variance | Std. dev. |
| --- | --- | --- | --- |
| ID | (Intercept) | 1.1311 | 1.0635 |
| LogDays | (Intercept) | 4.2203 | 2.0543 |
| Tree | (Intercept) | 0.6803 | 0.8248 |
| Residual |  | 22.9295 | 4.7885 |

Number of observations: 1983, groups: ID, 162, DaysDiv100, 38, Tree, 4.

Table S2.3. Fixed effects of the model for gH+

|  | Estimate | Standard error | t value |
| --- | --- | --- | --- |
| (Intercept) | 21.03641 | 2.07031 | 10.161 |
| PPFDdiv100 | 0.49708 | 0.07115 | 6.987 |
| LogCHL | 0.19642 | 0.64113 | 0.306 |
| REDNESS | 6.74576 | 3.39366 | 1.988 |
| LogDays | -3.41436 | 1.14480 | -2.982 |

Table S2.4. Correlation of fixed effects of the model for gH+

|  | (Intercept) | PPFDdiv100 | LogCHL | REDNESS |
| --- | --- | --- | --- | --- |
| PPFDdiv100 | -0.024 |  |  |  |
| LogCHL | 0.623 | 0.013 |  |  |
| REDNESS | -0.238 | -0.006 | 0.364 |  |
| LogDays | -0.859 | -0.027 | 0.244 | 0.017 |

**Anova tests for the significance of the fixed effects of the model for gH+**

The tests were run on model results produced with the REML criterion relaxed.

Reduced model for the testing of the significance of the effect of PPFDdiv100

Red (PPFDdiv100): yeo.johnson(gH, 0.6) ~ LogCHL + REDNESS + LogDays + (1 | ID) + (1 | LogDays) + (1 | Tree)

Reduced model for the testing of the significance of the effect of LogCHL

Red (LogCHL): yeo.johnson(gH, 0.6) ~ PPFDdiv100 + REDNESS + LogDays + (1 | ID) + (1 | LogDays) + (1 | Tree)

Reduced model for the testing of the significance of REDNESS

Red (REDNESS): yeo.johnson(gH, 0.6) ~ PPFDdiv100 + LogCHL + LogDays + (1 | ID) + (1 | LogDays) + (1 | Tree)

Reduced model for the testing of the significance of the effect of LogDays

Red (LogDays): yeo.johnson(gH, 0.6) ~ PPFDdiv100 + LogCHL + REDNESS + (1 | ID) + (1 | LogDays) + (1 | Tree)

Full model: yeo.johnson(gH, 0.6) ~ PPFDdiv100 + LogCHL + REDNESS + LogDays + (1 | ID) + (1 | LogDays) + (1 | Tree)

Table S2.5. Results from Anova tests of the significance of the fixed effects of the model for gH+

|  | npar | AIC | BIC | logLik | deviance | χ^2^ | Df | Pr(>χ^2^) | Signif. |
| --- | --- | --- | --- | --- | --- | --- | --- | --- | --- |
| Red (PPFDdiv100) | 8 | 12012 | 12063 | -5997.2 | 12042 | 47.758 | 1 | 4.821 x 10^-12^ | *** |
| Red (LogCHL) | 8 | 12010 | 12055 | -5997.2 | 11994 | 0.1069 | 1 | 0.7436 |  |
| Red (REDNESS) | 8 | 12014 | 12059 | -5999.2 | 11998 | 3.9582 | 1 | 0.04664 | * |
| Red (LogDays) | 8 | 12018 | 12063 | -6001.2 | 12002 | 8.1105 | 1 | 0.004401 | ** |
| Full model | 9 | 12012 | 12063 | -5997.2 | 11994 |  |  |  |  |

**Characterization of the residuals of the model for gH+**

Fig. S2.1. A histogram of the residuals of the model for gH+

Fig. S2.2. Quantiles of the residuals of the model for gH+ as a function of the quantiles of normal distribution.

Fig. S2.3. Residuals of the model for gH+ as a function of the fitted values.

**3. A Linear Mixed Effect Model for vH^+^**

**Justification of model variables**

The flux of protons through the thylakoid membrane, vH^+^, depends on the conductivity of the membrane, gH^+^, and PPFD is included because light causes the flux of protons toward the lumen. Chlorophyll content and the number of days after 2 Sep 2021 describe the advancement of autumn senescence, and redness is the main topic of the study. Possibility of a leaf temperature effect is obvious for a flux parameter. Interactions between gH+ and chlorophyll content, leaf temperature and redness index were additionally tested. Random effects of specific sample, tree, leaf and date were considered possible.

**Call of the R function lmer**

L_vh ~ PPFDdiv1000 + gHdiv100 + gHdiv100 * PPFDdiv1000 + CHLdiv100 + gHdiv100 * CHLdiv100 + gHdiv100 * LfTempdiv100 + LfTempdiv100 + LogDays + REDNESS + gHdiv100 * REDNESS + (1 | ID) + (1 |Tree) + (1 | Leaf) + (1 | Date)

**Variable conversions and data filtering applied in the model for vH+**

The response variable vH+ vas logit transformed after removing data points lower or equal than 0.005 or higher or equal than 0.2. Chlorophyll concentration was expressed as (µg cm^-2^)/100 (CHLdiv100), PPFD was expressed in mmol m^-2^s^-1^ (PPFDdiv1000), the gH value reported by PhotosynQ was divided by 100 (gHdiv100), chlorophyll concentration was expressed as (µg cm^-2^)/100 (CHLdiv100), leaf temperature was expressed as °C/100 (LfTempdiv100), days after 2 Sep 2021 was log transformed (LogDays).

All data were filtered by removing all rows with chlorophyll content lower or equal than 0.5 µg cm^-2^.

vH+ values outside of the range 0.005 < vH+ < 0.2 were removed.

**Summary of the model for vH+**

Linear mixed model fit by REML ['lmerMod']

REML criterion at convergence: -2216.5

Table S3.1. Scaled residuals of the model for vH+

| Min | 1Q | Median | 3Q | Max |
| --- | --- | --- | --- | --- |
| -4.12 | -0.6033 | 0.0664 | 0.6361 | 3.3890 |

Table S3.2. Random effects of the model for vH+

| Groups | Name | Variance | Std. dev. |
| --- | --- | --- | --- |
| ID | (Intercept) | 3.156 x 10^-3^ | 0.056176 |
| Date | (Intercept) | 1.292 x 10^-2^ | 0.113676 |
| Leaf | (Intercept) | 4.033 x 10^-5^ | 0.006351 |
| Tree | (Intercept) | 2.544 x 10^-3^ | 0.13614 |
| Residual |  |  |  |

Number of observations: 2189, groups: ID, 162; Date, 38; Leaf, 8; Tree, 4.

Table S3.3. Fixed effects of the model for vH+

|  | Estimate | Standard error | t value |
| --- | --- | --- | --- |
| (Intercept) | -2.34613 | 0.11134 | -21.072 |
| PPFDdiv100 | 1.76959 | 0.06425 | 27.541 |
| gHdiv100 | 0.53434 | 0.07132 | 7.492 |
| CHLdiv100 | 0.76549 | 0.07721 | 9.914 |
| LfTempdiv100 | 0.30384 | 0.47064 | 0.646 |
| LogDays | 0.06528 | 0.05924 | 1.102 |
| REDNESS | -0.52220 | 0.18132 | -2.880 |
| PPFDdiv1000:gHdiv100 | -0.90791 | 0.06562 | -13.835 |
| PPFDdiv1000:CHLdiv100 | -0.49293 | 0.09966 | -4.946 |
| gHdiv100:LfTempdiv100 | -0.43737 | 0.53125 | -0.823 |
| gHdiv100:REDNESS | -0.31506 | 0.21978 | -1.434 |

Table S3.4. Correlation of fixed effects of the model for vH+

|  | (Intercept) | PPFD |  |  |  |  |  |  |  |  |
| --- | --- | --- | --- | --- | --- | --- | --- | --- | --- | --- |
| PPFDdiv100 | -0.003 |  |  |  |  |  |  |  |  |  |
| gHdiv100 | -0.383 | 0.071 |  |  |  |  |  |  |  |  |
| CHLdiv100 | -0.195 | 0.042 | 0.169 |  |  |  |  |  |  |  |
| LfTempdiv100 | -0.557 | -0.052 | 0,683 | -0.093 |  |  |  |  |  |  |
| LogDays | -0.796 | -0.032 | -0.030 | 0.094 | 0.082 |  |  |  |  |  |
| REDNESS | -0.126 | -0.075 | 0.191 | 0.132 | 0.086 | 0.004 |  |  |  |  |
| PPFDdiv1000:gHdiv100 | 0.018 | -0.947 | -0.092 | -0.046 | 0.032 | 0.025 | 0.071 |  |  |  |
| gHdiv100:CHLdiv100 | 0.067 | -0.081 | -0.253 | -0.779 | 0.074 | 0.038 | -0.033 | 0.093 |  |  |
| gHdiv100:LeafTempdiv100 | 0.362 | 0.02 | -0.913 | 0.110 | -0.759 | 0.023 | -0.105 | -0.016 | -0.102 |  |
| gHdiv100:REDNESS | 0.137 | 0.070 | -0.277 | -0.077 | -0.124 | -0.032 | -0.808 | -0.067 | 0.076 | 0.154 |

**Anova tests for the significance of the fixed effects of the model for vH+**

The REML criterion was relaxed when fitting the models for anova tests.

Reduced model for the testing of the significance of the effect of PPFD

Reduced(PPFD): L_vH ~ gHdiv100 + CHLdiv100 + gHdiv100 * CHLdiv100 + gHdiv100 * LfTempdiv100 + LfTempdiv100 + LogDays + REDNESS + gHdiv100 * REDNESS + (1 | ID) + (1 | Tree) + (1 | Leaf) + (1 | Date)

Reduced model for the testing of the significance of gH+

Reduced(gH): L_vH ~ PPFDdiv1000 + CHLdiv100 + LfTempdiv100 + LogDays + REDNESS + (1 | ID) + (1 | Tree) + (1 | Leaf) + (1 | Date)

Note: Due to differences in the amounts of missing or discarded data in different columns of the data table, all records with missing data were omitted from the significance calculations.

Reduced model for the testing of the significance of the effect of chlorophyll content

Reduced(CHL): L_vH ~ PPFDdiv1000 + gHdiv100 + gHdiv100 * PPFDdiv1000 + gHdiv100 * LfTempdiv100 + LfTempdiv100 + LogDays + REDNESS + gHdiv100 * REDNESS + (1 | ID) + (1 | Tree) + (1 | Leaf) + (1 | Date)

Reduced model for the testing of the significance of leaf temperature

Reduced(LfTemp): L_vH ~ PPFDdiv1000 + gHdiv100 + gHdiv100 * PPFDdiv1000 + CHLdiv100 + gHdiv100 * CHLdiv100 + LogDays + REDNESS + gHdiv100 * REDNESS + (1 | ID) + (1 | Tree) + (1 | Leaf) + (1 | Date)

Reduced model for the testing of the significance of REDNESS

Reduced(REDNESS): L_vH ~ PPFDdiv1000 + gHdiv100 + gHdiv100 * PPFDdiv1000 + CHLdiv100 + gHdiv100 * CHLdiv100 + gHdiv100 * LfTempdiv100 + LfTempdiv100 + LogDays + (1 | ID) + (1 | Tree) + (1 | Leaf) + (1 | Date)

Reduced model for the testing of the significance of the interaction between gH+ and PPFD

Reduced(gH*PPFD): L_vH ~ PPFDdiv1000 + gHdiv100 + CHLdiv100 + gHdiv100 * CHLdiv100 + gHdiv100 * LfTempdiv100 + LfTempdiv100 + LogDays + REDNESS + gHdiv100 * REDNESS + (1 | ID) + (1 | Tree) + (1 | Leaf) + (1 | Date)

Reduced model for the testing of the significance of the interaction between gH+ and chlorophyll content

Reduced(gH*CHL): L_vH ~ PPFDdiv1000 + gHdiv100 + gHdiv100 * PPFDdiv1000 + CHLdiv100 + gHdiv100 * LfTempdiv100 + LfTempdiv100 + LogDays + REDNESS + gHdiv100 * REDNESS + (1 | ID) + (1 | Tree) + (1 | Leaf) + (1 | Date)

Reduced model for the testing of the significance of the interaction between gH+ and leaf temperature

Reduced(gH*LfTemp): L_vH ~ PPFDdiv1000 + gHdiv100 + gHdiv100 * PPFDdiv1000 + CHLdiv100 + gHdiv100 * CHLdiv100 + LfTempdiv100 + LogDays + REDNESS + gHdiv100 * REDNESS + (1 | ID) + (1 | Tree) + (1 | Leaf) + (1 | Date)

Reduced model for the testing of the significance of the interaction between gH+ and redness

Reduced(gH*REDNESS): L_vH ~ PPFDdiv1000 + gHdiv100 + gHdiv100 * PPFDdiv1000 + CHLdiv100 + gHdiv100 * CHLdiv100 + gHdiv100 * LfTempdiv100 + LfTempdiv100 + LogDays + REDNESS + (1 | ID) + (1 | Tree) + (1 | Leaf) + (1 | Date)

Full model: L_vH ~ PPFDdiv1000 + gHdiv100 + gHdiv100 * PPFDdiv1000 + CHLdiv100 + gHdiv100 * CHLdiv100 + gHdiv100 * LfTempdiv100 + LfTempdiv100 + LogDays + REDNESS + gHdiv100 * REDNESS + (1 | ID) + (1 | Tree) + (1 | Leaf) + (1 | Date)

Table S3.5. Anova tests for the significance of the fixed effects of the model for vH+

|  | npar | AIC | BIC | logLik | deviance | χ^2^ | Df | Pr(>χ^2^) | Signif. |
| --- | --- | --- | --- | --- | --- | --- | --- | --- | --- |
| Reduced (PPFD) | 14 | -708.93 | -629.26 | 368.47 | -736.93 | 1516.2 | 2 | <2.2 x 10^‑16^ | *** |
| Reduced (gH) | 11 | -1608.6 | -1546.0 | 815.28 | -1630.6 | 622.56 | 5 | <2.2 x 10^‑16^ | *** |
| Reduced(CHLdiv100) | 14 | -2112.8 | -2033.1 | 1070.4 | 2140.8 | 112.34 | 2 | <2.2 x 10^‑16^ | *** |
| Reduced(LfTemp) | 14 | -2224.4 | -2144.8 | 1126.2 | -2252.4 | 0.6745 | 2 | 0.7137 |  |
| Reduced(REDNESS) | 14 | -2178.6 | -2098.9 | 1103.3 | 2206.6 | 46.503 | 2 | 7.98 x 10^‑11^ | *** |
| Reduced(gH*PPFD) | 15 | -2039.3 | -1954.0 | 1034.7 | -2069.3 | 183.79 | 1 | <2.2 x 10^‑16^ | *** |
| Reduced(gH*CHL) | 15 | -2198.8 | -2113.5 | 1114.4 | 2228.8 | 24.272 | 1 | 8.364 x 10^-7^ | *** |
| Reduced(gH*LfTEmp) | 15 | -2222.4 | -2137.1 | 1126.2 | -2252.4 | 0.6721 | 1 | 0.4123 |  |
| Reduced(gH*REDNES) | 15 | -2221.1 | -2135.7 | 1125.5 | -2251.1 | 2.0693 | 1 | 0.1503 |  |
| Full model | 16 | -2221.1 | -130.06 | 1126.6 | -2253.12 |  |  |  |  |

**Characterization of the residuals of the model for vH+**

Fig. S3.1. Histogram of the residuals of the model for vH+

Fig. S3.2. Quantiles of the residuals of the model for vH+ as a function of the quantiles of normal distribution.

Fig. S3.3. Residuals of the model for vH+ as a function of the fitted values.

**4. A Linear Model for F_V_/F_M_**

**Justification of model variables**

F_V_/F_M_ values were measured from a separate batch of leaf samples, from which also the redness index and chlorophyll content were measured.

**Variable conversions and data filtering applied in the model for F_V_/F_M_**

The response variable FvFm was Yeo-Johnson transformed with λ = 6.5, and chlorophyll content was transformed by dividing the original μg/cm2 value by 100.

**Call of the R function lm**

lm(formula = yeo.johnson(fvfmdata[, 1], 6.5) ~ CHLdiv100 + REDNESS + CHLdiv100 * REDNESS, data = fvfmdata)

Table S4.1. Residuals of the model for F_V_/F_M_

| Min | 1Q | Median | 3Q | Max |
| --- | --- | --- | --- | --- |
| -3.1776 | -0.6083 | 0.1303 | 0.5819 | 3.2340 |

Table S4.2. Coefficients of the model for F_V_/F_M_

|  | Estimate | Standard error | t value | Pr(<\|t\|) | Significance |
| --- | --- | --- | --- | --- | --- |
| (Intercept) | 4.3356 | 0.1358 | 31.918 | <2 x 10^-16^ | *** |
| CHLdiv100 | 5.2059 | 0.6914 | 7.529 | 9.09 x 10^-13^ | *** |
| REDNESS | -7.3567 | 0.9225 | -7.975 | 5.34 x 10^-14^ | *** |
| CHLdiv100:REDNESS | 4.1148 | 7.9212 | 0.519 | 0.604 |  |

**Significance of the model for F_V_/F_M_**

F(3,252) =113.8, p <2.2 x 10^‑16^.

**Characterization of the residuals of the model for F_V_/F_M_**

Fig. S4.1. A histogram of the residuals of the model for F_V_/F_M_.


Fig. S4.2. Quantiles of the residuals of the model for F_V_/F_M_ as a function of the quantiles of normal distribution.

Fig. S4.3. A plot of the residuals of the F_V_/F_M_ model as a function of the fitted values.

Fig. S4.4. F_V_/F_M_ as a function of the chlorophyll content of the leaf.

Fig. S4.5. F_V_/F_M_ as a function of the redness index.

**5. Beta Regression Model for F_V_'/F_M_'**

**Justification of model variables**

F_V_’/F_M_’ is known to depend on PPFD whereas chlorophyll content and Julian day (LogDays variable) describe the advancement of autumn senescence, and redness is the main topic of the study. Sunny days are expected to cause photoinhibition that lowers F_V_’/F_M_’, and therefore the total radiation parameter of the day (SateiWm2) was included. Inspection of the data suggested that a low temperature of the previous night may strongly affect F_V_’/F_M_’, and a variable describing the lowest temperature of previous night (PrvDLTmpDv100) was therefore included. However, cold night may be associated with a cold day, and therefore also leaf temperature (LfTemp) was included, as well as its interaction with PrvDLTempDv100. Photoinhibition may be associated with low temperature, and therefore the interaction term SateiWm2*PrvTLTmpDv100 was included. PSI may be important for repair of PSII after photoinhibition, and therefore the relative number of open PSI centres (PSIOpenCtrs) was included. Low temperature might specifically affect during late autumn, and therefore the interaction LfTemp*LogDays was included. In addition to the obvious terms, leaf thickness and relative humidity were included.

**Call of the R function betareg**

betareg(formula = yeo.johnson(vax[, 21], 1.8) ~ PPFDdiv100 + CHLdiv100 + PPFDdiv100 * CHLdiv100 + REDNESS + LfTemp + PrvDLTmpDv100 + LfTemp * LogDays + LfTemp * PrvDLTmpDv100 + SateiWm2 + SateiWm2 * PrvDLTmpDv100 + Thickness + Humidity + PS1OpenCtrs, data = vax)

**Variables, their conversions and data filtering applied in the model for F_V_’/F_M_’**

The data were filtered by removing all samples with chlorophyll content less than 5 µg cm^-2^.

Values of F_V_’/F_M_’ were Yeo-Johnson transformed with λ = 1.8; PPFD was expressed in µmol m^‑2^s^‑1^/100 (PPFDdiv100); Chlorophyll concentration was expressed as (µg cm^-2^)/100 (CHLdiv100), Leaf temperature in °C (LfTemp); Lowest temperature of previous night in °C/10 (PrvDLTmpDv100); Number of days after 2 Sep 2021 was log transformed (LogDays); Total radiation during the day was expressed in Wm^-2^ (SateiWm2); Leaf thickness was expressed in mm (Thickness); Relative humidity was expressed in per cent (Humidity); The number of open PSI centres per leaf area is a relative value (PS1OpenCtrs).

**Summary of the model for F_V_’/F_M_’**

Table S5.1. Standardized weighted residuals of the model for F_V_’/F_M_’

| Min | 1Q | Median | 3Q | Max |
| --- | --- | --- | --- | --- |
| -3.5923 | -0.4436 | 0.1288 | 0.6092 | 5.2363 |

Table S5.2. Coefficients (mean model with logit link) of the model for F_V_’/F_M_’

|  | Estimate | Std. Error | z value. | Pr(>\|z\|) |  |
| --- | --- | --- | --- | --- | --- |
| (Intercept) | 2.465 | 0.3868 | 6.372 | 1.86 x 10^-10^ | *** |
| PPFDdiv100 | -0.5182 | 0.02095 | -24.739 | <2 x 10^-16^ | *** |
| CHLdiv100 | 3.232 | 0.1353 | 23.894 | <2 x 10^-16^ | *** |
| REDNESS | -1.219 | 0.2376 | -5.130 | 2.90 x 10^-7^ | *** |
| LfTemp | -0.1314 | 0.02824 | -4.651 | 3.30 x 10^-6^ | *** |
| PrvDLTmpDv100 | 4.049 | 2.417 | 1.675 | 0.093884 | . |
| LogDays | -2.433 | 0.2009 | -12.109 | <2 x 10^‑16^ | *** |
| SateiWm2 | 5.208 x 10^-5^ | 2.594 x 10^-5^ | -2.008 | 0.044667 | * |
| Thickness | 0.1298 | 0.03782 | 3.432 | 0.000599 | *** |
| Humidity | 7.926 x 10^-3^ | 1.372 x 10^-3^ | 5.777 | 7.62 x 10^-9^ | *** |
| PS1OpenCtrs | 0.01786 | 7.945 x 10^-3^ | -2.248 | 0.024591 | * |
| PPFDdiv100:CHLdiv100 | 0.5150 | 0.06973 | 7.385 | 1.52 x 10^-13^ | *** |
| LfTemp:LogDays | 0.1425 | 0.01438 | 9.908 | <2 x 10^‑16^ | *** |
| LfTemp:PrvDLTmpDv100 | -0.09022 | 0.1913 | -0.472 | 0.637192 |  |
| SateiWm2:PrvDLTmpDv100 | -5.996 x 10^-4^ | 3.013 x 10^-4^ | -1.990 | 0.046561 | * |

ɸ coefficients (precision model with identity link):

|  | Estimate | Std. Error | z value | Pr(>\|z\|) |  |
| --- | --- | --- | --- | --- | --- |
| (ɸ) | 45.714 | 1.628 | 28.08 | <2 x 10^-16^ | *** |

**Characterization of the residuals of the model for F_V_’/F_M_’**

Fig. S5.1. Histogram of the residuals of the model for F_V_'/F_M_'

Fig. S5.2. Quantiles of the residuals of the model for F_V_'/F_M_' as a function of the quantiles of normal distribution.

Fig. S5.3. The residuals of the model for F_V_'/F_M_' as a function of the fitted values.

**6. Linear Mixed Effect Model for Leaf Thickness**

**Justification of model variables**

Leaf thickness might change during the autumn senescence, and therefore chlorophyll content (CHLdiv100) and Julian day (LogDays) were included. Redness is the main topic of the study. Leaf temperature and humidity were included to test for physical effects of temperature expansion and moisture content. Random variation due to leaf, site on leaf and tree was also considered.

**Call of the R function lmer**

## Thickness^0.85 ~ log(CHLdiv100) + REDNESS + LogDays + Humidity + LfTemp + (1 | Site) + (1 | Leaf) +

## (1 | Tree)

**Variable conversions and data filtering applied in the model of Thickness**

Leaf thickness, expressed in mm, was raised to the power 0.85;; Chlorophyll concentration, expressed as (µg cm^-2^)/100, was log transformed (log(CHLdiv100)); The redness index was used as such (REDNESS); The number of days after Sep. 2, 2021 was log transformed (LogDays); Relative humidity was expressed as per cent (Humidity); Leaf temperature was expressed as °C.

The data were filtered by removing all rows with chlorophyll content lower or equal than 0.5 µg cm^‑2^.

**Summary of the model for leaf thickness**

Linear mixed model fit by REML ['lmerMod']

REML criterion at convergence: -2298.1

Table S6.1. Scaled residuals of the model for leaf thickness

| Min | 1Q | Median | 3Q | Max |
| --- | --- | --- | --- | --- |
| -3.8670 | -0.6016 | -0.0411 | 0.5592 | 4.9433 |

Table S6.2. Random effects of the model for leaf thickness

| Groups | Name | Variance | Std. dev. |
| --- | --- | --- | --- |
| Leaf | (Intercept) | 0.001547 | 0.03933 |
| Site | (Intercept) | 0.026862 | 0.16390 |
| Tree | (Intercept) | 0.001418 | 0.03765 |
| Residual |  | 0.023870 | 0.15450 |

Number of observations: 2686, groups: ID, 162.

Table S6.3. Fixed effects of the model for leaf thickness

|  | Estimate | Standard error | t value |
| --- | --- | --- | --- |
| (Intercept) | 0.7029229 | 0.0773666 | 9.086 |
| log(CHLdiv100) | 0.0568365 | 0.0044645 | 12.731 |
| REDNESS | 1.0762424 | 0.0648000 | 16.609 |
| LogDays | -0.0184175 | 0.0129179 | -1.426 |
| Humidity | 0.0005014 | 0.0003995 | 1.255 |
| LfTemp | -0.0130176 | 0.0016029 | -8.121 |

Table S6.4. Correlation of fixed effects of the model for leaf thickness

|  | (Intercept) | log(CHLdiv100) | REDNESS | LogDays | Humidity |
| --- | --- | --- | --- | --- | --- |
| log(CHLdiv100) | -0.012 |  |  |  |  |
| REDNESS | -0.037 | 0.530 |  |  |  |
| LogDays | -0.213 | 0.336 | 0.031 |  |  |
| Humidity | -0.156 | 0.066 | 0.173 | -0.272 |  |
| LfTemp | -0.230 | -0.028 | -0.023 | 0.363 | -0.363 |

**Anova tests for the significance of the fixed effects of the model for leaf thickness**

The full and reduced models were fitted with the REML criterion relaxed.

Reduced model for the testing of the significance of the effect of log(CHLdiv100)

Thickness^0.85 ~ REDNESS + LogDays + Humidity + LfTemp + (1 | Site) + (1 | Leaf) + (1 | Tree)

Reduced model for the testing of the significance of the effect of REDNESS

Thickness^0.85 ~ log(CHLdiv100) + LogDays + Humidity + LfTemp + (1 | Site) + (1 | Leaf) + (1 | Tree)

Reduced model for the testing of the significance of the effect of LogDays

Thickness^0.85 ~ log(CHLdiv100) + REDNESS + Humidity + LfTemp + (1 | Site) + (1 | Leaf) + (1 | Tree)

Reduced model for the testing of the significance of the effect of Humidity

Thickness^0.85 ~ log(CHLdiv100) + REDNESS + LogDays + LfTemp + (1 | Site) + (1 | Leaf) + (1 | Tree)

Reduced model for the testing of the significance of the effect of LfTemp

Thickness^0.85 ~ log(CHLdiv100) + REDNESS + LogDays + Humidity + (1 | Site) + (1 | Leaf) + (1 | Tree)

Full model: Thickness^0.85 ~ log(CHLdiv100) + REDNESS + LogDays + Humidity + LfTemp + (1 | Site) + (1 | Leaf) + (1 | Tree)

Table S6.5. Results of anova tests for the significance of the fixed effects of the model for leaf thickness

|  | npar | AIC | BIC | logLik | deviance | χ^2^ | Df | Pr(>χ^2^) | Signif. |
| --- | --- | --- | --- | --- | --- | --- | --- | --- | --- |
| Red (log(CHLdiv100)) | 9 | -2172.3 | -2119.2 | 1095.1 | -2190.3 | 156.56 | 1 | <2.2 x 10^‑16^ | *** |
| Red (REDNESS) | 9 | -2066.5 | -2013.5 | 1042.3 | -2084.5 | 262.31 | 1 | <2.2 x 10^‑16^ | *** |
| Red (LogDays) | 9 | -2326.8 | -2273.7 | 1172.4 | -2344.8 | 2.0371 | 1 | 0.1535 |  |
| Red (Humidity) | 9 | -2327.3 | -2274.2 | 1172.7 | -2345.3 | 1.5473 | 1 | 0.2135 |  |
| Red (LfTemp) | 9 | -2264.5 | -2211.4 | 1141.2 | -2282.5 | 64.339 | 1 | 1.048 x 10^‑15^ | *** |
| Full model | 10 | -2326.8 | -2267.9 | 1173.4 | -2346.8 |  |  |  |  |

**Characterization of the residuals of the model for leaf thickness**

Fig. S6.1. Histogram of the residuals of the model for leaf thickness

Fig. S6.2. Quantiles of the residuals of the model for leaf thickness as a function of the quantiles of normal distribution.

Fig. S6.3. Residuals of the model for leaf thickness as a function of the fitted values.

Fig. S6.4. Leaf thickness, in mm, as a function of the redness index.

Fig. S6.5. Leaf thickness, in mm, as a function of the chlorophyll content of the leaf, in μg cm^-2^.

**7. Linear Mixed Effect Model for NPQt**

**Justification of model variables**

Chlorophyll content (LogCHL) and Julian day (LogDays) describe the advancement of the autumn senescence. Redness is the main topic of the study. PPFD is the well-known inducer of NPQ. The lowest temperature of the previous night (PrvDLTmpDv100) was included because preliminary inspection of the data suggested that it affects another fluorescence parameter, F_V_’/F_M_’. Leaf temperature might have an effect on the induction of NPQ because NPQ depends on lumen acidity, which, in turn, depends on the function of the carbon reduction cycle. Humidity and leaf thickness were included as physical factors that might affect the measurement rather than physiology, and random variation due to tree, leaf and date as included.

**Call of the R function lmer**

yeo.johnson(NPQt, -2.9) ~ LogCHL + REDNESS + PPFDdiv100 + LogDays + PrvDLTmpDv100 + Humidity + Thickness + LfTemp + (1 | Tree) + (1 | Leaf) + (1 | Date)

**Variable conversions and data filtering applied in the model for NPQt**

The response variable NPQt was Yeo-Johnson transformed with λ=-2.9: Chlorophyll concentration was Log transformed (LogCHL); The redness index was used as such (REDNESS); PPFD, expressed in µmol m^-2^s^-1^, was divided by 100 (PPFDdiv100); Number of days after 2 Sep 2021 was log transformed (LogDays); The lowest temperature of previous night, measured in °C, was divided by 100 (PrvDLTmpDv100); Relative humidity was expressed in per cent (Humidity); Leaf temperature was expressed in °C.

The data were filtered by accepting only records with chlorophyll content at least 5 µg cm^-2^, F_M_’ value between 500 and 4000, and 25.0 was considered as the highest acceptable NPQt value.

**Summary of the model for NPQt**

Linear mixed model fit by REML ['lmerMod']

REML criterion at convergence: -8763.4

Table S7.1. Scaled residuals of the model for NPQt

| Min | 1Q | Median | 3Q | Max |
| --- | --- | --- | --- | --- |
| -3.4697 | -0.6623 | -0.0180 | 0.5668 | 3.5743 |

Table S7.2. Random effects of the model for NPQt

| Groups | Name | Variance | Std. dev. |
| --- | --- | --- | --- |
| Date | (Intercept) | 7.909 x 10^-4^ | 0.028123 |
| Leaf | (Intercept) | 2.010 x 10^-5^ | 0.004483 |
| Tree | (Intercept) | 1.868 x 10^-5^ | 0.004322 |
| Residual |  | 1.705 x 10^-4^ | 0.013059 |

Number of observations: 630, groups: ID, 162; Date, 30.

Table S7.3. Fixed effects of the model for NPQt

|  | Estimate | Standard error | t value |
| --- | --- | --- | --- |
| (Intercept) | 0.3616616 | 0.0271588 | 13.317 |
| LogCHL | -0.0624026 | 0.0027113 | -23.016 |
| REDNESS | 0.0532980 | 0.0130353 | 4.089 |
| PPFDdiv100 | 0.0043697 | 0.0002199 | 19.869 |
| LogDays | 0.0215713 | 0.0146872 | 1.469 |
| PrvDLTmpDv100 | -0.1810122 | 0.1405137 | -1.288 |
| Humidity | -0.0002476 | 0.0001462 | -1.694 |
| Thickness | 0.0019730 | 0.0015714 | 1.256 |
| LfTemp | 0.0012146 | 0.0003752 | 3.237 |

Table S7.4. Correlation of fixed effects of the model for NPQt

|  | (Intercept) | LogCHL | REDNESS | PPFDdiv100 | LogDays | PrvDLTmpDv100 | Humidity | Thickness |
| --- | --- | --- | --- | --- | --- | --- | --- | --- |
| LogCHL | -0.164 |  |  |  |  |  |  |  |
| REDNESS | -0.051 | 0.349 |  |  |  |  |  |  |
| PPFDdiv100 | 0.018 | 0.055 | 0.015 |  |  |  |  |  |
| LogDays | -0.848 | 0.069 | 0.000 | -0.016 |  |  |  |  |
| PrvDLTmpDv100 | -0.517 | -0.002 | 0.019 | 0.022 | 0.275 |  |  |  |
| Humidity | -0.254 | 0.044 | -0.011 | 0.021 | -0.056 | -0.098 |  |  |
| Thickness | -0.061 | -0.138 | -0.294 | -0.065 | 0.015 | -0.033 | 0.017 |  |
| LfTemp | -0.201 | -0.090 | -0.098 | -0.220 | 0.041 | -0.090 | 0.139 | 0.332 |

**Anova tests for the significance of the fixed effects of the model for F_V_’/F_M_’**

The REML criterion was relaxed for the fitting of the models.

Reduced model for testing the significance of the effect of LogCHL

Red(LogCHL): yeo.johnson(NPQt, -2.9) ~ REDNESS + PPFDdiv100 + LogDays + PrvDLTmpDv100 + Humidity + Thickness + LfTemp + (1 | Tree) + (1 | Leaf) + (1 | Date)

Reduced model for testing the significance of effect of REDNESS

Red(REDNESS): yeo.johnson(NPQt, -2.9) ~ LogCHL + PPFDdiv100 + LogDays + PrvDLTmpDv100 + Humidity + Thickness + LfTemp + (1 | Tree) + (1 | Leaf) + (1 | Date)

Reduced model for the testing of the significance of the effect of PPFDdiv100

Red(PPFDdiv100): yeo.johnson(NPQt, -2.9) ~ LogCHL + REDNESS + LogDays + PrvDLTmpDv100 + Humidity + Thickness + LfTemp + (1 | Tree) + (1 | Leaf) + (1 | Date)

Reduced model for the testing of the significance of the effect of LogDays

Red(LogDays): yeo.johnson(NPQt, -2.9) ~ LogCHL + REDNESS + PPFDdiv100 + PrvDLTmpDv100 + Humidity + Thickness + LfTemp + (1 | Tree) + (1 | Leaf) + (1 | Date)

Reduced model for the testing of the significance of PrvDLTmpDv100

Red(PrvDLTmpDv100): yeo.johnson(NPQt, -2.9) ~ LogCHL + REDNESS + PPFDdiv100 + LogDays + Humidity + Thickness + LfTemp + (1 | Tree) + (1 | Leaf) + (1 | Date)

Reduced model for the testing of the significance of the effect of Humidity

Red(Humidity): yeo.johnson(NPQt, -2.9) ~ LogCHL + REDNESS + PPFDdiv100 + LogDays + PrvDLTmpDv100 + Thickness + LfTemp + (1 | Tree) + (1 | Leaf) + (1 | Date)

Reduced model for the testing of the significance of the effect of Thickness

Red(Thickness): yeo.johnson(NPQt, -2.9) ~ LogCHL + REDNESS + PPFDdiv100 + LogDays + PrvDLTmpDv100 + Humidity + LfTemp + (1 | Tree) + (1 | Leaf) + (1 | Date)

Reduced model for the testing of the significance of the effect of LfTemp

Red(LfTemp): yeo.johnson(NPQt, -2.9) ~ LogCHL + REDNESS + PPFDdiv100 + LogDays + PrvDLTmpDv100 + Humidity + Thickness + (1 | Tree) + (1 | Leaf) + (1 | Date)

Full model: full: yeo.johnson(NPQt, -2.9) ~ LogCHL + REDNESS + PPFDdiv100 + LogDays + PrvDLTmpDv100 + Humidity + Thickness + LfTemp + (1 | Tree) + (1 | Leaf) + (1 | Date)

Table S7.5. Results of Anova tests for the significance of the fixed effects of the model for NPQt

|  | npar | AIC | BIC | logLik | deviance | χ^2^ | Df | Pr(>χ^2^) | Signif. |
| --- | --- | --- | --- | --- | --- | --- | --- | --- | --- |
| LogCHL | 12 | -8375.1 | -8310.9 | 4199.5 | -8399. | 454.93 | 1 | <2.2 x 10^‑16^ | *** |
| REDNESS | 12 | -8813.7 | -8749.5 | 4418.8 | -8837.7 | 16.339 | 1 | 5.296 x 10^-5^ | *** |
| PPFDdiv100 | 12 | -8478.1 | -8413.9 | 4251 | -8502.1 | 351.95 | 1 | <2.2 x 10^‑16^ | *** |
| LogDays | 12 | -8827.7 | -8763.6 | 4425.9 | -8851.7 | 2.271 | 1 | 0.1318 |  |
| PrvDLTmpDv100 | 12 | -8828.3 | -8764.1 | 4426.1 | 8852.3 | 1.7494 | 1 | 0.186 |  |
| Humidity | 12 | -8827.4 | -8763.2 | 4425.7 | -8851.4 | 2.6518 | 1 | 0.1034 |  |
| Thickness | 12 | -8828.4 | -8764.3 | 4426.2 | -8852.4 | 1.5862 | 1 | 0.2079 |  |
| LfTemp | 12 | -8819.5 | -8755.4 | 4421.8 | -8843.5 | 10.5 | 1 | 0.001194 | ** |
| Full model | 13 | -8828.0 | -8758.5 | 4427.0 | -8854.0 |  |  |  |  |

**Characterization of the residuals of the model for NPQt**

Fig. S7.1. Histogram of residuals of the model for NPQt.

Fig. S7.2. Quantiles of the residuals of the model for NPQt as a function of quantiles of normal distribution.

Fig. S7.3. Residuals of the model for NPQt as a function of the fitted values.

**8. A Linear Mixed Effect Model for Relative Number of Active PSI Centres**

**Justification of model variables**

Chlorophyll content (LogCHL) and Julian day (LogDays) describe the advancement of the autumn senescence and redness is the main topic of the study. The number of PSI centres can obviously be associated with both chlorophyll content and leaf thickness, as the number of PSI units per leaf area may obviously depend on the number and size of cells in a vertical cross section. Leaf temperature was included as it might affect the activity of PSI, as PSI photoinhibition is known to occur in the cold. Fandom variation due to measurement date was also considered.

**Call of the R function lmer**

## lmer(yeo.johnson(vaa2[, 34], 8.3) ~ LogDays + LogCHL + REDNESS + Thickness + LfTemp + (1 | Date))

**Variable conversions and data filtering applied in the model for Relative Number of Active PSI Centres**

The response variable PSIAct was raised to the power of 0.1 and Yeo-Johnson transformed with λ = 8.3; Number of days after 2 Sep 2021 was log transformed (LogDays); Chlorophyll concentration, expressed as (µg cm^-2^), was log transformed (LogCHL); The redness index was used as such (REDNESS); Leaf thickness was measured in mm (Thickness); Leaf temperature was measured in °C (LfTemp).

The data were filtered by removing all rows with chlorophyll content lower or equal than 0.5 µg cm^-2^. Relative values of active centres were limited to 0.0<PSIActCntrs<5.0.

**Summary of the model for Relative Number of Active PSI Centres**

Linear mixed model fit by REML ['lmerMod']

REML criterion at convergence: 10066.4.

Table S8.1. Scaled residuals of the model for Relative Number of Active PSI Centres

| Min | 1Q | Median | 3Q | Max |
| --- | --- | --- | --- | --- |
| -4.1895 | -0.5128 | 0.0171 | 0.5171 | 3.5757 |

Table S8.2. Random effects of the model for Relative Number of Active PSI Centres

| Groups | Name | Variance | Std. dev. |
| --- | --- | --- | --- |
| Date | (Intercept) | 43.48 | 6.594 |
| Residual |  | 87.44 | 9.351 |

Number of observations: 1367, groups: Date, 36.

Table S8.3. Fixed effects of the model for Relative Number of Active PSI Centres

|  | Estimate | Standard error | t value |
| --- | --- | --- | --- |
| (Intercept) | 37.8673 | 7.0870 | 5.343 |
| LogDays | -4.4001 | 3.5775 | -1.230 |
| LogCHL | 15.1288 | 1.7742 | 8.527 |
| REDNESS | -0.4224 | 7.3132 | -0.058 |
| Thickness | -0.4793 | 1.1165 | -0.429 |
| LfTemp | -0.3042 | 0.2175 | -1.399 |

Table S8.4. Correlation of fixed effects of the model for Relative Number of Active PSI Centres

|  | (Intr) | LogDays | LogCHL | REDNESS | Thickness |
| --- | --- | --- | --- | --- | --- |
| LogDays | -0.836 |  |  |  |  |
| LogCHL | -0.498 | 0.182 |  |  |  |
| REDNESS | -0.140 | 0.015 | 0.188 |  |  |
| Thickness | -0.161 | 0.058 | -0.049 | -0.233 |  |
| LfTemp | -0.548 | 0.203 | 0.056 | 0.079 | 0.211 |

**Anova tests for the significance of the fixed effects of the model for Relative Number of Active PSI Centres**

The full and reduced models were fitted with the REML criterion relaxed.

Reduced model for testing the significance of the effect of LogDays

Red (LogDays): yeo.johnson(PS1ActCtrs, 8.3) ~ LogCHL + REDNESS + Thickness + LfTemp + (1 | Date)

Reduced model for testing the significance of the effect of LogCHL

Red (LogCHL): yeo.johnson(PS1ActCtrs, 8.3) ~ LogDays + REDNESS + Thickness + LfTemp + (1 | Date)

Reduced model for testing the significance of the effect of REDNESS

Red(REDNESS): yeo.johnson(PS1ActCtrs, 8.3) ~ LogDays + LogCHL + Thickness + LfTemp + (1 | Date)

Reduced model for testing the significance of Thickness

Red(Thickness): yeo.johnson(PS1ActCtrs, 8.3) ~ LogDays + LogCHL + REDNESS + LfTemp + (1 | Date)

Reduced model for testing the significance of LfTemp

Red(LfTemp): yeo.johnson(PS1ActCtrs, 8.3) ~ LogDays + LogCHL + REDNESS + Thickness + (1 | Date)

Full model: yeo.johnson(PSIActCtrs, 8.3) ~ LogCHL yeo.johnson(vaa2[, 34], 8.3) ~ LogDays + LogCHL + REDNESS + Thickness + LfTemp + (1 | Date)

|  | npar | AIC | BIC | logLik | deviance | χ^2^ | Df | Pr(>χ^2^) | Signif. |
| --- | --- | --- | --- | --- | --- | --- | --- | --- | --- |
| Red(LogDays) | 7 | 10098 | 10134 | -5041.9 | 10084 | 1.5915 | 1 | 0.2071 |  |
| Red(LogCHL) | 7 | 10167 | 10204 | -5076.7 | 10153 | 71.088 | 1 | <2.2 x 10^‑16^ | *** |
| Red(REDNESS) | 7 | 10096 | 10133 | -5041.1 | 10081 | 0.0054 | 1 | 0.9417 |  |
| Red(Thickness) | 7 | 10096 | 10133 | -5041.2 | 10082 | 0.1759 | 1 | 0.6749 |  |
| Red(LfTemp) | 7 | 10098 | 10135 | -5042.1 | 10082 | 1.9884 | 1 | 0.1585 |  |
| Full model | 8 | 10098 | 10140 | -5041.1 | 10082 |  |  |  |  |

**Characterization of the residuals of the model for Relative Number of Active PSI Centres**

Fig. S8.1. A histogram of the residuals of the model for the relative number of active PSI centres.

Fig. S8.2. Quantiles of the residuals of the model for active PSI centres as a function of the quantiles of normal distribution.

Fig. S8.3. Residuals of the model for the relative number of active PSI centres as a function of the fitted values of the model.

**9. Linear model for the rate constant of photoinhibition (k_PI_)**

**Justification of model variables**

The rate constant of photoinhibition (k_PI_) can be calculated from *in vivo* treatments in the presence of lincomycin. In addition to the characteristics of the leaves (chlorophyll content and redness index and their interaction), the color of the illumination treatment, and interaction of the light color with both chlorophyll content and redness index were included, as well as the ternary interaction between light color, chlorophyll content and redness. Descriptive statistics suggested that the leaf type (Green, Green senescent, Yellow or Red) may affect, and this variable was included.

**Call of the R function lm**

lm(formula = kpi_data[, 9]^0.4 ~ LeafType+REDNESS +Chlorophyll + Light + Light * REDNESS + Light * Chlorophyll + Chlorophyll * REDNESS + Chlorophyll * REDNESS * Light, data = kpi_data)

**Variables, their conversions and data filtering applied in the model for k_PI_**

The dependent variable, k_PI_, was raised to the power 0.4. The color of the photoinhibition light was coded as 2=green, 3=red, 4=white light and the leaf types were 1 = Green, 2 = Green senescent, 3 = Yellow, 4 = Red. Chlorophyll content was expressed as μg cm^-2^.

**Summary of the model for k_PI_**

Table S9.1. Standardized weighted residuals of the model for k_PI_

| Min | 1Q | Median | 3Q | Max |
| --- | --- | --- | --- | --- |
| -0.058038 | -0.017377 | -0.003632 | 0.014262 | 0.091604 |

Table S9.2. Coefficients (mean model with logit link) of the model for k_PI_

|  | Estimate | Std. Error | t value. | Pr(>\|z\|) |  |
| --- | --- | --- | --- | --- | --- |
| (Intercept) | -0.0605441 | 0.0363320 | -1.666 | 0.0989 | . |
| LeafType | 0.0155858 | 0.0068703 | 2.269 | 0.0256 | * |
| REDNESS | -0.3746745 | 0.2526831 | -1.483 | 0.1414 |  |
| Chlorophyll | 0.0001061 | 0.0014954 | 0.071 | 0.9436 |  |
| Light | 0.0695743 | 0.0094610 | 7.354 | 6.75 x 10^-11^ | *** |
| REDNESS:Light | 0.0558661 | 0.0810937 | 0.689 | 0.4926 |  |
| Chlorophyll:Light | -0.0004459 | 0.0004445 | -1.003 | 0.3183 |  |
| REDNESS:Chlorophyll | 0.0244042 | 0.0228073 | 1.070 | 0.2873 |  |
| REDNESS:Chlorophyll:Light | -0.0046846 | 0.0068992 | -0.679 | 0.4988 |  |

**Significance of the model for k_PI_**

F(8,95)=50.3, p < 2.2 x 10-^16^.

**Characterization of the residuals of the model for k_PI_**

Fig. S9.1. A histogram of the residuals of the model for k_PI_.

Fig. S9.2. Quantiles of the residuals of the model for k_PI_ as a function of quantiles of normal distribution.

Fig. S9.3. Residuals of the model for k_PI_ as a function of the fitted values.

**10. A Linear Model for Recovery from Photoinhibition in the Presence or Absence of Lincomycin**

**Justification of model variables**

The chlorophyll content of the leaf, describing the degree of senescence, was included, as well as the redness index that was the main topic of the study, and the interaction between the two. The color of the light used in the photoinhibition treatment preceding the recovery is not expected to affect recovery, which was done in the same weak white light for all samples, but the variable was included to rule out an unexpected effect. The aim was to equalize the percent decrease in of F_V_/F_M_ during the preceding photoinhibition treatment but as some differences remained, the Severity variable, calculated as F_V_/F_M__inhibited / F_V_/F_M__control, was included, as it may affect the recovery. The presence or absence of lincomycin was naturally included.

The response variable was defined as (FvFm after rec - FvFm after inh)/(FvFm control - FvFm after inh).

The color of photoinhibition light was coded as 2=green, 3=red, 4=white, and the presence of lincomycin was coded as 1=present, 2=not present.

**Variable conversions and data filtering applied in the model for recovery from photoinhibition in the presence or absence or lincomycin**

Six samples with |recovery|>2.0 were removed.

Normal distribution achieved by raising to the power 0.9.

**Call of the R function lm**

## lm(formula = pi_rec3_nolm_data[, 9]^0.9 ~ Light + Chlorophyll + REDNESS + Severity + LM+ Chlorophyll * REDNESS, data = pi_rec3_nolm_data)

Table S10.1. Residuals of the model for recovery from photoinhibition in the presence or absence of lincomycin

| Min | 1Q | Median | 3Q | Max |
| --- | --- | --- | --- | --- |
| -0.5090 | -0.1280 | -0.0020 | 0.1325 | 0.7134 |

Table S10.2. Coefficients of the model for recovery from photoinhibition in the presence or absence of lincomycin

|  | Estimate | Standard error | t value | Pr(<\|t\|) | Significance |
| --- | --- | --- | --- | --- | --- |
| (Intercept) | 0.671903 | 0.054391 | 12.353 | < 2 x 10^-16^ | *** |
| Light | 0.01850 | 0.017548 | 1.054 | 0.29308 |  |
| Chlorophyll | 0.003692 | 0.001658 | 2.226 | 0.02720 | ** |
| REDNESS | -0.7241412 | 0.304982 | -2.365 | 0.01902 | * |
| Severity | 0.119037 | 0.068575 | 1.736 | 0.08421 | * |
| LM | -0.316404 | 0.028220 | -11.212 | < 2 x 10^-16^ | *** |
| Chlorophyll:REDNESS | 0.062780 | 0.022549 | 2.784 | 0.00591 | ** |

**Significance of the model for recovery from photoinhibition in the presence or absence of lincomycin**

F(6,190) =36.62, p < 2.2 x 10^-16^.

**Characterization of the residuals of the model for recovery from photoinhibition in the presence or absence of lincomycin**

Fig. S10.1. A histogram of the residuals of the model for the recovery from photoinhibition in the presence or absence of lincomycin.

Fig. S10.2. Quantiles of the residuals of the model for the recovery of photoinhibition in the presence or absence of lincomycin as a function of quantiles of normal distribution.

Fig. S10.3. A plot of the residuals of the model for the recovery from photoinhibition in the presence or absence of lincomycin as a function of the fitted values.

**11. A Linear Model for Recovery from Photoinhibition in the Absence of Lincomycin**

**Justification of model variables**

The leaf type (green, green senescent, yellow, red) and chlorophyll content of the leaf, describing the degree of senescence, was included, as well as the redness index that was the main topic of the study, and the interaction between the two. The color of the light used in the photoinhibition treatment preceding the recovery is not expected to affect recovery, which was done in the same weak white light for all samples, but the variable was included to rule out an unexpected effect. The aim was to equalize the percent decrease in of F_V_/F_M_ during the preceding photoinhibition treatment but as some differences remained, the Severity variable, calculated as F_V_/F_M__inhibited / F_V_/F_M__control, was included, as it may affect the recovery.

The response variable was defined as (F_V_/F_M_ _after recovery - F_V_F_M__after illumination) / (F_V_F_M__control - F_V_F_M_ _after illumination).

**Variable conversions and data filtering applied in the model for recovery from photoinhibition**

Six samples with |recovery|>2.0 were removed.

Normal distribution achieved by raising to the power 0.8.

**Call of the R function lm**

lm(formula = pi_rec3_nolm_data[, 9]^0.8 ~ Type + Light + Chlorophyll + REDNESS + Severity + Chlorophyll * REDNESS, data = pi_rec3_nolm_data)

Table S11.1. Residuals of the model for recovery from photoinhibition in the absence of lincomycin

| Min | 1Q | Median | 3Q | Max |
| --- | --- | --- | --- | --- |
| -0.40153 | -0.09969 | -0.01905 | 0.10311 | 0.58261 |

Table S11.2. Coefficients of the model for recovery from photoinhibition in the absence of lincomycin

|  | Estimate | Standard error | t value | Pr(<\|t\|) | Significance |
| --- | --- | --- | --- | --- | --- |
| (Intercept) | 0.8996756 | 0.1637796 | 5.493 | 3.78 x 10^-7^ | *** |
| Leaf_type | -0.1063886 | 0.0491954 | -2.163 | 0.0333 | * |
| Light | -0.0001415 | 0.0241926 | -0.006 | 0.9953 |  |
| Chlorophyll | 0.0006799 | 0.0035430 | 0.192 | 0.8483 |  |
| REDNESS | 0.2301414 | 0.5231987 | 0.440 | 0.6611 |  |
| Severity | 0.2279863 | 0.1125989 | 2.025 | 0.0459 | * |
| Chlorophyll:REDNESS | 0.0339570 | 0.0287805 | 1.180 | 0.2412 |  |

**Significance of the model for recovery from photoinhibition in the absence of lincomycin**

F(6,88) =10.54, p =8.65 X 10^-9^.

**Characterization of the residuals of the model for recovery from photoinhibition in the absence of lincomycin**

Fig. S11.1. A histogram of the residuals of the model for the recovery from photoinhibition in the absence of lincomycin.

Fig. S11.2. Quantiles of the residuals of the model for the recovery of photoinhibition in the absence of lincomycin as a function of quantiles of normal distribution.

Fig. S11.3. A plot of the residuals of the model for the recovery from photoinhibition in the absence of lincomycin as a function of the fitted values.
